# Supplementary material for: Food additive mixtures and type 2 diabetes incidence: Results from the NutriNet-Santé prospective cohort
Source: PLoS Med. 2025 Apr 8;22(4):e1004570. doi: 10.1371/journal.pmed.1004570 (PMC11977966; doi:10.1371/journal.pmed.1004570)
Supplement: S1 Checklist — (DOC) [file pmed.1004570.s001.doc]

STROBE Statement—Checklist of items that should be included in reports of ***cohort studies***

|  | Item No | Recommendation | Relevant text from manuscript^a^ |
| --- | --- | --- | --- |
| **Title and abstract** | 1 | (*a*) Indicate the study’s design with a commonly used term in the title or the abstract | Title  Abstract section: Paragraph “Methods and Findings” |
|  |  | (*b*) Provide in the abstract an informative and balanced summary of what was done and what was found |  |
| Introduction | | |  |
| Background/rationale | 2 | Explain the scientific background and rationale for the investigation being reported | Introduction section: Paragraphs 1-4 |
| Objectives | 3 | State specific objectives, including any prespecified hypotheses | Introduction section: Paragraph 4-5 |
| Methods | | |  |
| Study design | 4 | Present key elements of study design early in the paper | Methods section: Study population paragraph |
| Setting | 5 | Describe the setting, locations, and relevant dates, including periods of recruitment, exposure, follow-up, and data collection | Following paragraphs in the Methods section: Study population, Dietary data collection, Food additive intakes, Type 2 diabetes ascertainment  S1 Appendix: eMethod1, eMethod2 and eMethod3 |
| Participants | 6 | (*a*) Give the eligibility criteria, and the sources and methods of selection of participants. Describe methods of follow-up | Methods section: Study population paragraph  S1 Appendix: eMethod 1 |
|  |  | (*b*) For matched studies, give matching criteria and number of exposed and unexposed | NA |
| Variables | 7 | Clearly define all outcomes, exposures, predictors, potential confounders, and effect modifiers. Give diagnostic criteria, if applicable | Method section: Dietary data collection, Food additive intakes, Type 2 diabetes ascertainment, Statistical analyses paragraph |
| Data sources/ measurement | 8* | For each variable of interest, give sources of data and details of methods of assessment (measurement). Describe comparability of assessment methods if there is more than one group | Method section: Dietary data collection, Food additive intakes, Type 2 diabetes ascertainment, Statistical analyses  Discussion section: Strengths and limitations |
| Bias | 9 | Describe any efforts to address potential sources of bias | Method section & Discussion section: Strengths and limitations paragraph |
| Study size | 10 | Explain how the study size was arrived at | S1 Appendix: eMethod 4 |
| Quantitative variables | 11 | Explain how quantitative variables were handled in the analyses. If applicable, describe which groupings were chosen and why | Methods section, Statistical analyses paragraph  S1 Appendix: eMethod 2 (food additive intakes estimation) |
| Statistical methods | 12 | (*a*) Describe all statistical methods, including those used to control for confounding | Methods section, Statistical analyses paragraph  Discussion section, Strengths and limitations paragraph  S1 Appendix: eMethod 4, Non-Negative matrix factorisation, Multiple imputation for missing values, sensitivity analyses |
|  |  | (*b*) Describe any methods used to examine subgroups and interactions | Methods section, Statistical analyses paragraph |
|  |  | (*c*) Explain how missing data were addressed | S1 Appendix: eMethod 4, Multiple imputation for missing values paragraph |
|  |  | (*d*) If applicable, explain how loss to follow-up was addressed | Results section: Descriptive characteristics paragraph |
|  |  | (*e*) Describe any sensitivity analyses | Methods section: Statistical analyses paragraph  S1 Appendix: eMethod4 section, Sensitivity analyses paragraph, Table C, Table G, Table H, Table J |
| Results | | |  |
| Participants | 13* | (a) Report numbers of individuals at each stage of study—eg numbers potentially eligible, examined for eligibility, confirmed eligible, included in the study, completing follow-up, and analysed | Methods section: Statistical analyses paragraph  S1 Appendix: Figure A  Results section: Descriptive characteristics paragraph |
|  |  | (b) Give reasons for non-participation at each stage |  |
|  |  | (c) Consider use of a flow diagram |  |
| Descriptive data | 14* | (a) Give characteristics of study participants (eg demographic, clinical, social) and information on exposures and potential confounders | Results section: Table 1  S1 Appendix: Multiple Imputation for missing values  Results section: Associations between food additive mixtures and type 2 diabetes incidence |
|  |  | (b) Indicate number of participants with missing data for each variable of interest |  |
|  |  | (c) Summarise follow-up time (eg, average and total amount) |  |
| Outcome data | 15* | Report numbers of outcome events or summary measures over time | Results section: Associations between food additive mixtures and type 2 diabetes incidence |
| Main results | 16 | (*a*) Give unadjusted estimates and, if applicable, confounder-adjusted estimates and their precision (eg, 95% confidence interval). Make clear which confounders were adjusted for and why they were included | Methods section, Statistical analyses paragraph  Results section: Associations between food additive mixtures and type 2 diabetes incidence  All covariates are indicated in the footnotes to each Table  S1 Appendix: Table H for exposures in categories |
|  |  | (*b*) Report category boundaries when continuous variables were categorized |  |
|  |  | (*c*) If relevant, consider translating estimates of relative risk into absolute risk for a meaningful time period |  |
| Other analyses | 17 | Report other analyses done—eg analyses of subgroups and interactions, and sensitivity analyses | Results section: Associations between food additive mixtures and type 2 diabetes risk, third paragraph  S1 Appendix: Table C, eTable G, eTable H, eTable I, eTable J, eTable K, eTable L |
| Discussion | | |  |
| Key results | 18 | Summarise key results with reference to study objectives | Discussion section: Main findings paragraph |
| Limitations | 19 | Discuss limitations of the study, taking into account sources of potential bias or imprecision. Discuss both direction and magnitude of any potential bias | Discussion section: Strengths and limitations paragraph |
| Interpretation | 20 | Give a cautious overall interpretation of results considering objectives, limitations, multiplicity of analyses, results from similar studies, and other relevant evidence | Discussion section: Comparison with epidemiological literature paragraph, Mechanistic plausibility paragraph, Strengths and limitations paragraph |
| Generalisability | 21 | Discuss the generalisability (external validity) of the study results | Discussion section: Comparison with epidemiological literature paragraph, Strengths and limitations paragraph |
| Other information | | |  |
| Funding | 22 | Give the source of funding and the role of the funders for the present study and, if applicable, for the original study on which the present article is based | Fundings section  Conflict of Interest section |

*Give information separately for exposed and unexposed groups.

*^a^all elements cited are found in the main manuscript, unless the S1 Appendix is expressly mentioned*

**Note:** An Explanation and Elaboration article discusses each checklist item and gives methodological background and published examples of transparent reporting. The STROBE checklist is best used in conjunction with this article (freely available on the Web sites of PLoS Medicine at http://www.plosmedicine.org/, Annals of Internal Medicine at http://www.annals.org/, and Epidemiology at http://www.epidem.com/). Information on the STROBE Initiative is available at http://www.strobe-statement.org.
