# Supplementary material for: Food additive mixtures and type 2 diabetes incidence: Results from the NutriNet-Santé prospective cohort
Source: PLoS Med. 2025 Apr 8;22(4):e1004570. doi: 10.1371/journal.pmed.1004570 (PMC11977966; doi:10.1371/journal.pmed.1004570)
Supplement: S1 Appendix — Fig A. Flowchart, NutriNet-Santé cohort, 2009-2023. Table A. Daily food additive intakes (mg/d) among study participants from the NutriNet-Santé cohort, 2009–2023 (N = 108,643)a,b. Table B. Food additive mixtures identified by nonnegative matrix factorizationa. Table C. Consensus map for rank number determination and sensitivity analyses using other decomposition algorithms in the NMF procedure. Table D. Spearman correlations between the five NMF food additive mixtures. Table E. Spearman correlations between NMF food additive mixtures and food group intakes. Table F. Food group consumption of participants according to sex-specific quintiles of mixtures 2 and 5a. Fig B. Correlations between Schoenfeld residuals and timescale (age, y) from multivariable Cox models between food additive mixtures and type 2 diabetes incidence, NutriNet-Santé cohort, 2009–2023 (n = 108,643). Fig C. Dose-response associations between food additive mixtures and type 2 diabetes incidence, restricted cubic spline plots, NutriNet-Santé cohort, 2009–2023 (n = 108,643 participants; 1,131 incident cases). Table G. Associations between food additive mixtures and type 2 diabetes incidence, NutriNet-Santé cohort, 2009–2023—Sensitivity analyses. Table H. Association between food additive mixtures coded as tertiles and incidence of type 2 diabetes, NutriNet-Santé cohort, 2009–2023—Sensitivity analyses. Table I. Associations between food additive mixtures and incidence of type 2 diabetes, stratified by the Programme National Nutrition Santé-Guidelines Score 2 (PNNS-GS2), NutriNet-Santé cohort, 2009-2023—Sensitivity analyses. Table J. Association between mixtures 2 and 5 and type 2 diabetes incidence adjusted for the key food additives contributing to each mixture (residual method), NutriNet-Santé cohort, 2009–2023 (n = 108,643 participants; 1,131 incident cases) —Sensitivity analyses. Table K. Interactions between the key food additives contributing to mixtures 2 and 5, NutriNet-Santé cohort, 2009–202 [file pmed.1004570.s002.docx]

**S1 Appendix**

[eMethods 3](#_Toc189839938)

[eMethod1. Method for dietary data collection and the identification of under-reporters of energy intake 3](#_Toc189839939)

[eMethod2. Detailed qualitative and quantitative assessment of food additives 3](#_Toc189839940)

[eMethod3. Methodology for type 2 diabetes ascertainment 5](#_Toc189839941)

[eMethod4. Additional information on statistical analyses methods 6](#_Toc189839942)

[*Power calculation* 6](#_Toc189839943)

[*Non-negative matrix factorisation* 6](#_Toc189839944)

[*Multiple Imputation for missing values* 7](#_Toc189839945)

[*Sensitivity analyses* 7](#_Toc189839946)

[eResults 9](#_Toc189839947)

[Fig A. Flowchart, NutriNet-Santé cohort, 2009-2023 9](#_Toc189839948)

[Table A. Daily food additive intakes (mg/d) among study participants from the NutriNet-Santé cohort, 2009-2023 (N=108,643)^a,b^ 10](#_Toc189839949)

[Table B. Food additive mixtures identified by non-negative matrix factorisation^a^ 16](#_Toc189839950)

[Table C. Consensus map for rank number determination and sensitivity analyses using other decomposition algorithms in the NMF procedure 19](#_Toc189839951)

[C. a. Rank selection in NMF (consensus map) 19](#_Toc189839952)

[C. b. Food additive mixtures identified by non-negative matrix factorisation - Brunet algorithm: loading values of main additive contributors, NutriNet-Santé cohort, 2009-2023^a^ 20](#_Toc189839953)

[C. c. Food additive mixtures identified by Non-smooth non-negative matrix factorisation (nsNMF): loading values of main additive contributors, NutriNet-Santé cohort, 2009-2023^a^ 23](#_Toc189839954)

[C. d. Stability of the NMF food additive mixtures across time 26](#_Toc189839955)

[Table D. Spearman correlations between the five NMF food additive mixtures 30](#_Toc189839956)

[Table E. Spearman correlations between NMF food additive mixtures and food group intakes. 31](#_Toc189839957)

[Table F. Food group consumption of participants according to sex-specific quintiles of mixtures 2 and 5^a^. 32](#_Toc189839958)

[A. Mixture 2 32](#_Toc189839959)

[B. Mixture 5 35](#_Toc189839960)

[Fig B. Correlations between Schoenfeld residuals and timescale (age, y) from multivariable Cox models between food additive mixtures and type 2 diabetes incidence, NutriNet-Santé cohort, 2009-2023 (n=108,643). 38](#_Toc189839961)

[Fig C. Dose-response associations between food additive mixtures and type 2 diabetes incidence, restricted cubic spline plots, NutriNet-Santé cohort, 2009-2023 (n=108,643 participants ; 1,131 incident cases). 44](#_Toc189839962)

[Table G. Associations between food additive mixtures and type 2 diabetes incidence, NutriNet-Santé cohort, 2009-2023 - Sensitivity analyses 48](#_Toc189839963)

[Table H. Association between food additive mixtures coded as tertiles and incidence of type 2 diabetes, NutriNet-Santé cohort, 2009-2023 - Sensitivity analyses. 51](#_Toc189839964)

[Table I. Associations between food additive mixtures and incidence of type 2 diabetes, stratified by the Programme National Nutrition Santé – Guidelines Score 2 (PNNS-GS2), NutriNet-Santé cohort, 2009-2023 - Sensitivity analyses 52](#_Toc189839965)

[A. Below the sex-specific median 52](#_Toc189839966)

[B. Above the sex-specific median 52](#_Toc189839967)

[Table J. Association between mixtures 2 and 5 and type 2 diabetes incidence adjusted for the key food additives contributing to each mixture (residual method), NutriNet-Santé cohort, 2009-2023 (n=108,643 participants; 1,131 incident cases) - Sensitivity analyses 53](#_Toc189839968)

[Table K. Interactions between the key food additives contributing to mixtures 2 and 5, NutriNet-Santé cohort, 2009-2023 (n=108,643 participants; 1,131 incident cases) 54](#_Toc189839969)

[A. Mixture 2 54](#_Toc189839970)

[B. Mixture 5 55](#_Toc189839971)

[Table L. Mediation analyses 58](#_Toc189839972)

[A. Associations between the food groups most correlated with mixtures 2 and 5 and type 2 diabetes incidence, NutriNet-Santé cohort, 2009-2023 (n=108,643 participants; 1,131 incident cases) 58](#_Toc189839973)

[B. Proportion of the associations mediated by mixtures 2 and 5 58](#_Toc189839974)

[eReferences 59](#_Toc189839975)

# eMethods

## eMethod1. Method for dietary data collection and the identification of under-reporters of energy intake

At inclusion, and every six months thereafter, participants are invited to fill out three non-consecutive days of 24-hour dietary records, randomly assigned over a two-week period, including two weekdays and one weekend day (to account for variability in the diet across the week and the seasons). At all times throughout their assigned dietary record period, participants had access to a dedicated interface on the study website to declare all foods and beverages (including the commercial name/brand for industrial products) consumed during a 24h-period: three main meals (breakfast, lunch, dinner) and any other eating occasion. Participants were asked to estimate portion sizes either by directly entering the weight consumed in the platform, or by using validated photographs or usual containers.^1^ A French food composition database (>3,500 items) was used to estimate mean daily energy, alcohol, macro- and micro-nutrient intakes.^2^ These estimates included contributions from composite dishes using French recipes established by food and nutrition professionals. The web-based questionnaires used in the study have been tested and compared against both in-person interviews by trained dietitians^3^, and urinary and blood markers for energy and micro-/macro-nutrient intakes.^4,5^ In this analysis, we included participants having at least two 24h-dietary records during the first two years of follow-up.

Participants who under-reported their energy intake were excluded from the analyses and were identified using the method from Black, based on the original method developed by Goldberg et al.^6,7^ This method relies on the hypothesis that the maintenance of a stable body weight requires a balance between energy intake and expenditure. The equations developed by Black account for the reported dietary energy intake, basal metabolic rate (calculated using Schofield’s equations), sex, age, height, weight, number of dietary records, physical activity level (PAL), and intra/inter-individual variability.^8^ As recommended by Black, the intra-individual coefficients of variations for BMR and PAL were fixed at 8.5 % and 15%, respectively. In addition, a PAL of 1.55 was used to reflect a “light” physical activity which is assumed to be attained by healthy, normally active individual living a sedentary lifestyle. Finally, some individuals identified as under-reporters of energy intakes using Black’s method were not excluded, if they also reported recent weight variations, adherence to weight-loss restrictive diets, or declared the consumptions entered in their dietary records as unusually low compared to their habitual diets. In this study 23,098 participants (corresponding to 17.2% of the subjects) were considered as under-energy reporters and were excluded from the study. This proportion of under-reporters is common, for instance in the nationally representative INCA 3 study conducted in 2016 by the French Food Safety Agency 18% of adult participants were identified as under-reporters using the Black method.^9^

Several quality control operations were performed to account for over-reporting. Limitations in the online tool were set when participants reported the quantities of food consumed, aiming to alert them that the number they were about to enter was potentially an outlier, thereby encouraging double check and correction. Later on, during the data cleaning process, limitations were set per food category within one eating episode and per record for quantities; for instance, limitations for fruits were set for 3000 grams/day, 1500 grams/day for fish, 2000 grams/day for yoghurts, etc. if more than 10% of reported food items had outliers, then the full record was excluded. Otherwise, values were corrected to the maximum authorised values or standardised.

## eMethod2. Detailed qualitative and quantitative assessment of food additives

Each industrial food item consumed and reported in a specific dietary record was matched against three databases to assess the presence of any food additive: OQALI, a national database whose management has been entrusted to the National Research Institute for Agriculture, Food and Environment (INRAe) and the French food safety authority (ANSES) to characterize the quality of the food supply, Open Food Facts, an open collaborative database of food products marketed worldwide, and the Mintel Global New Products Database (GNPD), an online database of innovative food products in the world.^10^ In a second step, the dose of food additive contained in each food item was estimated based on (i) laboratory assays quantifying additives in specific food items (n=2,677 food-additive pairs analysed), (ii) doses in generic food categories provided by the European Food Safety Authority (EFSA), or (iii) generic doses from the Codex General Standard for Food Additives (GSFA)^11^. Dynamic matching was applied, meaning that products were matched date-to-date: the date of consumption of each food or beverage declared by each participant was used to match the product to the closest composition data available, thus accounting for potential reformulations.

The strength of our methodology relies in the precise qualitative assessment of additive exposure, i.e., presence/absence of a specific additive in the food consumed. This unique level of detail is permitted by the fact that commercial names/brands of industrial product consumed were collected and matched with Open Food Facts, Oqali and GNPD databases providing the ingredient list and thus, presence of the specific additive, at the time when the product was consumed. Thus, we only attribute a non-null dose of a specific additive to a given product declare by a participant if this specific product contains this specific additive. Then, the quantitative assessment of the doses of additives in the products which contain a specific additive is challenging since manufacturers are not compelled to declare this information on the packaging. Hence the three-step method used to assess doses in our cohort. In all, in the framework of the ADDITIVES project, we performed 2,677 quantified analyses, corresponding to a total of 61 food additives in 196 different (generic) food items. “Pairs” (i.e. a specific additive in a specific food vector) selected for laboratory assays corresponded to the most frequently consumed and most emblematic commercial food/beverage items for a given additive. In addition to the assays carried out by certified laboratories, which were sent to us by the consumer association UFC Que Choisir, we contacted two companies (Mérieux & Eurofins) and the Direction Générale de la Consommation, de la Concurrence et de la Répression des Fraudes (DGCCRF) to carry out these assays. Only the additives listed in their catalogue could be measured. In case data was not available from this source, EFSA and GSFA doses were only applied if the specific food item did actually contain the specific food additive in the ingredients list. EFSA collects many information from manufacturers related to their specific commercial products but for confidentiality reasons, only transfers information for generic food items or food groups (no brand-specific data). Additional information related to the estimation of food additive exposure in the NutriNet-Santé cohort have been published previously.^12^

## eMethod3. Methodology for type 2 diabetes ascertainment

Participants were asked to declare major health events though the yearly health questionnaire, through a specific health check-up questionnaire every six months, or at any time through a specific interface on the study website. They were also asked to declare all currently taken medications and treatments via the check-up and yearly questionnaires. A search engine with embedded exhaustive Vidal® drug database is used to facilitate medication data entry for the participants. Besides, our research team was the first in France to obtain the authorization by Decree in the Council of State (n°2013-175) to link data from our general population-based cohorts to medico-administrative databases of the National health insurance. Thus, data from the NutriNet-Santé cohort were linked to these medico-administrative databases, providing detailed information about the reimbursement of medication and medical consultations.

Regarding type 2 diabetes specifically: the 1,131 cases were primarily detected through the declaration by the participants of a type 2 diabetes diagnosed by a physician and/or diabetes medication use, in follow-up questionnaires. The questions were: “Have you been diagnosed with type 2 diabetes (if yes, indicate the date of diagnosis)” and “Are you treated for T2D?”. ATC codes considered for T2D medication were A10AB01, A10AB03, A10AB04, A10AB05, A10AB06, A10AC01, A10AC03, A10AC04, A10AD01, A10AD03, A10AD04, A10AD05, A10AE01, A10AE02, A10AE03, A10AE04, A10AE05, A10AE30, A10BA02, A10BB01, A10BB03, A10BB04, A10BB06, A10BB07, A10BB09, A10BB12, A10BD02, A10BD03, A10BD05, A10BD07, A10BD08, A10BD10, A10BD15, A10BD16, A10BF01, A10BF02, A10BG02, A10BG03, A10BH01, A10BH02, A10BH03, A10BX02, A10BX04, A10BX07, A10BX09, A10BX10, A10BX11, A10BX12.

In addition to the above-mentioned questions about diagnosis of type 2 diabetes and/or a medication report, two additional sources were considered to check for consistency. First, linkage with the medico-administrative databases confirmed more than 80% of the cases surveyed (ICD-10 codes E11). Of note: medico-administrative databases are not fully exhaustive and may miss some disease/medication information since they were not initially designed for research purpose. Second, among participants who provided a blood sample at the clinical/biological examination, 85.3 % of those with elevated fasting blood glucose (i.e., ≥1.26 g/L) had consistently reported a diagnosis of type 2 diabetes and/or medication. Of note, elevated blood glucose without any declaration of type 2 diabetes diagnosis or treatment was not considered specific enough to classify the participant as a type 2 diabetes case.

## eMethod4. Additional information on statistical analyses methods

## *Power calculation*

We conducted a statistical power analysis to ensure that our sample size would be sufficient to detect a clinically meaningful difference in hazard ratios between the compared groups. The power calculation was based on the log-rank test, tailored for survival analysis. This formula accounted for the expected proportion of exposed individuals (pE), the allocation ratio (pA), and the anticipated effect sizes. We preset the significance level (alpha) at 0.05 to control the risk of Type I error and sought a power of 80% (reflecting a beta of 0.20) to mitigate the risk of Type II error. With this sample size (n=108,643), number of incident cases (n=1,131), an alpha of 0.05, a statistical power of 80%, and a continuous food additive mixture exposure with a standard deviation (SD) of 10 for instance (corresponding to the SD of mixture 1), we were capable of detecting as “statistically significant” Hazard Ratio _for an increment of 1SD_≥1.09 or ≤0.92.

## *Non-negative matrix factorisation*

The aim of Nonnegative Matrix Factorisation (NMF) is the factorisation of a matrix, allowing to approximate its original information as precisely as possible, but with a reduced number of components. Compared to other factorisation methods such as principal component analysis (PCA), its particularity is the constraint of nonnegative values (both for the original data and for the outputted components), and that no independence constraints are imposed to the components. It is particularly adapted for sparse matrices such as ours, where individuals are described by a lot of food additives variables with many zeroes, and its prerequisite of nonnegative data is not problematic in the case of food consumption data. Also, compared with other factorisation methods, the absence of independence of components is interesting for food data approximation as an individual can combine several components generated by the NMF, which better considers the diversity of consumption behaviors within a population.^15^

Given a nonnegative matrix A, NMF seeks to produce rank-k matrices W and H such as:

$$A \approx W H$$

With W and H nonnegative and:

$$k \ll rank(A)$$

Each column of X is thus approximated by a non-negative linear combination of the columns of W (the basis components), where the coefficients are given by the corresponding column of H (the mixture coefficients).

The NMF algorithms estimates W and H as a local minimum of the following optimization problem:

$${min}_{W, H\geq0}\left[ D(X, WH) + R(W, H) \right]$$

With D a loss function that measures the approximation quality, and R an optional penalization function designed to improve desirable properties on matrices W and H. D can be a least squares criterion (Frobenius norm) or the Kullback-Leibler divergence. The generated matrices W and H extracted from the NMF were used to compute the correlation matrix.

The algorithms used and the number of ranks k can be determined after evaluation of several parameters, such as the quality of estimation (e.g. residuals, part of explained variance and sparseness^16^) or the stability after multiple runs (e.g. cophenetic correlation coefficient).^13^ We chose the Lee and Sung^17^algorithm for the main model according to those parameters. The number of ranks r was determined according to the method proposed by Brunet et al.^18^, using the smallest value of r for which the cophenetic coefficient starts decreasing, as visualized on the consensus map (eTable C.a.). We conducted sensitivity analyses by testing different NMF algorithms (instead of Lee and Sung), including Brunet^18^ and a modified version of Lee and Sung’s algorithm (i.e. non-smooth non-negative matrix factorisation, with the “nsNMF” option in the NMF package^19^). The identified mixtures and food additives that composed them were similar, regardless the chosen algorithm (shown in eTable C.b. and C.c). We checked the stability of food additive mixture intakes over time by performing NMF analysis on two periods of 7.5 years each (corresponding to the median follow-up: period 1= 2009-2016; period 2=2017-2024, eTable C.d.).

## *Multiple Imputation for missing values*

Missing values for covariates were handled by a multiple imputation approach using additive regression, followed by bootstrapping, and predictive mean matching (n=20 imputed dataset) as implemented in the *Hmisc* R package.^20^ Specifically, the imputation model included a comprehensive set of predictors deemed relevant to the missing covariates. Variables were incorporated to capture the underlying relationships and patterns. The choice of predictors (i.e., age, sex, Body Mass Index (BMI), physical activity, smoking status, number of smoked cigarettes in pack-years, educational level, family history of type 2 diabetes, number of dietary records, socio-professional categories, monthly household income per consumption unit, intakes of energy without alcohol, saturated fatty acids, sodium, dietary fibre, alcohol, added sugars) was guided by their known or hypothesized associations with the variables containing missing values. Missing values were imputed for the following variables: physical activity level (14% of missing values), smoking status (0.3%), education level (0.9%), BMI (3%), height (3%), number of smoked cigarettes in pack-years (0.3%), family history of type 2 diabetes (0.3%), socio-professional category (0.4%) and monthly income per household unit (12.1%).

## *Sensitivity analyses*

Sensitivity analyses were tested: Model 1: Main model + excluding cases diagnosed during the first two years to challenge reverse causality. Model 2: Main model + adjustment for diagnosis and/or treatment for at least one prevalent metabolic disorder (i.e. cardiovascular disease, arterial hypertension, hypertriglyceridemia). Model 3: Main model + mutual adjustment for the other NMF food additive mixtures. Model 4: Main model + Healthy and Western dietary patterns derived by factor analysis (continuous). Model 5: Main model with adjustment for food groups instead of nutrient variables: fruits and vegetables (continuous, g/d), dairy products (continuous, ml/day), red and processed meats (continuous, g/d). Model 6: Main model + indicator of health-seeking behaviours (total number of screening tests or exams in the lifecourse: CT colonography, colonoscopy or sigmoidoscopy, screening test for blood in stools, PAP smear, skin cancer screening exam, mammography, medical breast palpation, PSA blood testing, and digital rectal examination). Model 7: Main model + region (10 regions included: North (Nord-Pas-de-Calais, Haute-Normandie, Basse-Normandie, Picardie), North-East (Alsace, Champagne-Ardenne, Lorraine, Bourgogne, Franche-Comté), West (Bretagne, Pays-de-la-Loire), Center (Center region), South-West (Aquitaine, Midi-Pyrénées, Languedoc-Roussillon, Limousin, Poitou-Charentes), South-East (Auvergne, Rhône-Alpes, Provence-Alpes-Côte d'Azur), Île-de-France, French overseas territories and departments (Guadeloupe, Martinique, Guyane, La Réunion, collectivités d'outre-mer), Corsica, and 'Other' (French-speaking participants living abroad).

In addition, for mixtures 2 and 5 (for which we observed associations with higher type 2 diabetes incidence):

1. In order to investigate whether the associations between mixtures and type 2 diabetes incidence were in fact entirely driven by one or some specific additives (and not the mixture itself), we adjusted the main Cox model for each food additive characteristic of the mixture, using the residual method. To do so, we performed residual analyses: linear regression models of mixture 2 (resp. mixture 5) on each individual food additive characteristic of the mixture were used to extract residuals. These residuals were then included into the main Cox models to assess the association between mixtures and type 2 diabetes incidence, independent from the variability due to each main additive composing the mixture.
2. Interactions between the key food additives contributing to mixture 2 (resp. mixture 5) on type 2 diabetes incidence were formally tested (two by two interactions). Each food additive has been standardized to have a mean of 0 and a standard deviation of 1, enhancing interpretability.

# eResults

## Fig A. Flowchart, NutriNet-Santé cohort, 2009-2023

**
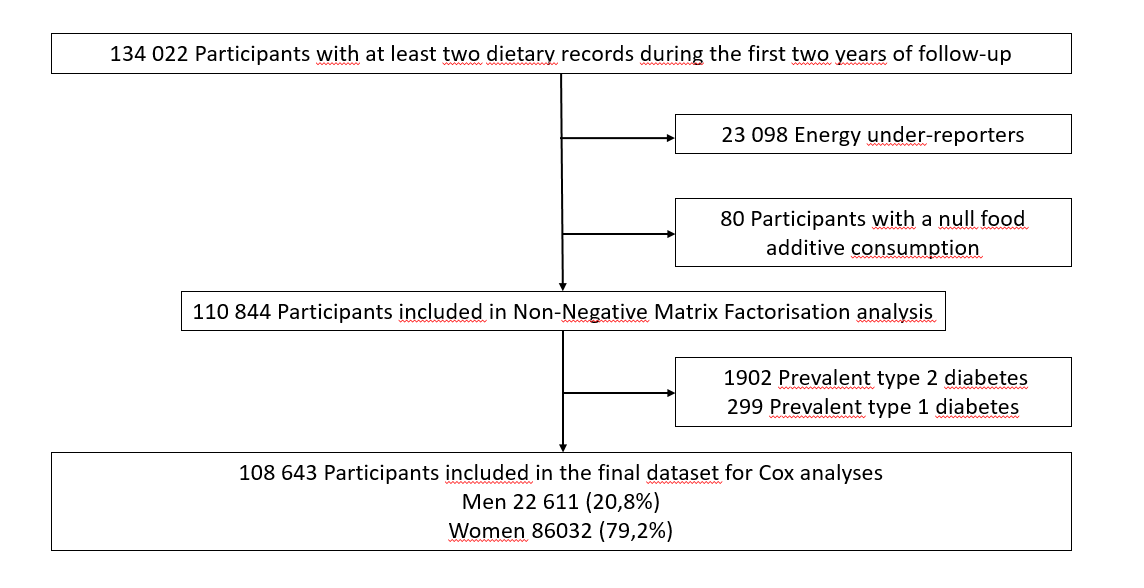
**

## Table A. Daily food additive intakes (mg/d) among study participants from the NutriNet-Santé cohort, 2009-2023 (N=108,643)^a,b^

| **Food additive name** | **European code** | **Mean (SD)** | **Median (25th – 75^th^ percentiles)** | **% consumers** |
| --- | --- | --- | --- | --- |
| Citric acid | E330 | 350.6 (546.0) | 223.1 (0.0-3.3) | 91.8 |
| Modified starches | E1400, E1403, E1404, E1412, E1414, E1420, E1422, E1440, E1442, E1450 | 1426.3 (1145.9) | 1154.3 (0.1-6.6) | 91.3 |
| Lecithins | E322 | 65.3 (150.3) | 42.9 (0.1-1.0) | 87.2 |
| Sulphites | E220, E221, E222, E223, E224, E225, E228, added sulphites - unspecified | 4.6 (5.8) | 2.6 (0.1-0.4) | 83.7 |
| Ascorbic acid | E300 | 72.0 (92.0) | 40.8 (0.0-0.1) | 83.6 |
| Pectins | E440 | 257.0 (316.7) | 163.3 (1.0-8.9) | 80.9 |
| Xanthan gum | E415 | 166.1 (236.2) | 76.8 (0.1-0.5) | 80.6 |
| Mono- and diglycerides of fatty acids | E471 | 219.4 (227.4) | 157.0 (445.8-2131.0) | 79.9 |
| Carrageenan | E407 | 91.8 (113.8) | 55.5 (0.1-0.7) | 75.6 |
| Sodium nitrite | E250 | 0.3 (0.3) | 0.2 (0.1-0.2) | 73.8 |
| Sodium carbonates | E500 | 2000.1 (2207.8) | 1312.5 (0.1-0.2) | 72.5 |
| Guar gum | E412 | 232.0 (243.7) | 162.0 (1.0-4.2) | 70.7 |
| Diphosphates | E450 | 528.7 (551.4) | 357.1 (0.2-1.4) | 70.1 |
| Potassium sorbate | E202 | 25.5 (33.1) | 14.2 (0.0-0.1) | 65.6 |
| Sodium erythorbate | E316 | 15.6 (19.4) | 9.1 (0.1-0.7) | 52.8 |
| Sodium ascorbate | E301 | 13.3 (12.8) | 9.8 (0.0-0.1) | 50.2 |
| Paprika extract, capsanthin, capsorubin | E160c | 0.4 (0.6) | 0.2 (0.1-1.4) | 48.0 |
| Sodium citrates | E331 | 221.3 (354.0) | 107.1 (0.1-4.5) | 47.4 |
| Ammonium carbonates | E503 | 724.0 (970.7) | 400.0 (32.0-143.9) | 46.5 |
| Locust bean gum | E410 | 73.3 (93.8) | 44.6 (6.3-20.0) | 45.4 |
| Carotenes | E160a | 3.2 (12.7) | 0.0 (1.5-26.8) | 42.7 |
| Lactic acid | E270 | 8.3 (29.9) | 3.4 (1.3-6.5) | 37.7 |
| Carotene | E160 | 0.6 (1.3) | 0.0 (12.0-62.1) | 37.0 |
| Acesulfame K | E950 | 12.8 (21.7) | 5.2 (88.4-667.5) | 33.5 |
| Potassium nitrate | E252 | 0.6 (0.7) | 0.4 (0.2-0.6) | 32.6 |
| Lutein | E161b | 1.2 (3.3) | 0.0 (9.7-56.8) | 28.5 |
| Sorbitols | E420 | 198.2 (302.8) | 109.9 (1.0-1.9) | 27.9 |
| Aspartame | E951 | 32.2 (50.5) | 14.7 (0.0-0.6) | 27.4 |
| Cochineal, Carminic acid, Carmines | E120 | 0.5 (1.2) | 0.2 (0.0-0.2) | 27.2 |
| Sulphite ammonia caramel | E150d | 551.4 (795.7) | 285.6 (0.0-0.2) | 26.2 |
| Glycerol | E422 | 561.6 (660.6) | 362.6 (0.1-0.5) | 26.2 |
| Calcium propionate | E282 | 47.6 (46.8) | 33.5 (0.2-0.5) | 25.2 |
| Curcumin | E100 | 2.4 (4.0) | 0.7 (0.3-2.5) | 25.1 |
| Triphosphates | E451 | 138.2 (204.1) | 72.6 (0.0-0.6) | 23.3 |
| Plain caramel | E150a | 22.1 (41.4) | 6.4 (1.2-6.3) | 23.3 |
| Carnosic acid (Extracts of rosemary) | E392 | 1.9 (2.7) | 1.0 (2.6-10.2) | 22.1 |
| Polyphosphates | E452 | 163.8 (250.2) | 77.0 (50.0-750.0) | 21.6 |
| Phosphoric acid | E338 | 64.9 (78.8) | 40.1 (1.5-7.7) | 20.7 |
| Monosodium glutamate | E621 | 315.4 (523.5) | 74.3 (1.2-9.2) | 19.4 |
| Calcium phosphates | E341 | 255.2 (571.4) | 114.3 (2.2-14.6) | 18.7 |
| Annatto, Bixin, Norbixin | E160b | 0.2 (0.2) | 0.1 (5.6-32.9) | 17.0 |
| Polyglycerol polyricinoleate | E476 | 22.3 (29.6) | 13.1 (11.4-34.3) | 15.2 |
| Sucralose | E955 | 9.7 (35.6) | 3.1 (3.1-9.9) | 15.1 |
| Sodium alginate | E401 | 47.4 (55.5) | 31.6 (0.8-5.9) | 14.1 |
| Mono and diacetyl tartaric acid esters of mono and diglycerides of fatty acids | E472e | 37.4 (67.5) | 20.8 (2.9-11.6) | 13.9 |
| Anthocyanins | E163 | 10.0 (17.7) | 5.3 (0.0-0.1) | 13.4 |
| Tocopherols | E306 | 3.6 (5.8) | 1.7 (0.0-0.0) | 13.3 |
| Alpha-tocopherol | E307 | 2.2 (3.7) | 1.0 (0.0-0.0) | 12.8 |
| Malic acid | E296 | 69.8 (146.3) | 27.9 (6.0-22.1) | 12.0 |
| Carboxy methyl cellulose | E466 | 59.2 (227.2) | 16.3 (0.0-0.1) | 11.2 |
| Lactic acid esters of mono- and diglycerides of fatty acids | E472b | 182.5 (263.5) | 80.5 (0.1-0.4) | 11.0 |
| Carnauba wax | E903 | 4.1 (7.6) | 1.7 (0.0-0.3) | 10.9 |
| Arabic gum | E414 | 540.8 (1239.0) | 185.2 (0.2-0.8) | 10.7 |
| Cellulose | E460 | 109.9 (211.9) | 20.2 (56.5-193.5) | 10.5 |
| Sodium acetate | E262 | 22.7 (28.6) | 14.2 (7.1-28.3) | 10.2 |
| Acetic acid | E260 | 153.5 (161.1) | 104.8 (1.2-8.0) | 10.1 |
| Ammonium phosphatides | E442 | 13.1 (25.4) | 5.6 (1.4-5.3) | 9.6 |
| Beetroot Red, betanin | E162 | 5.1 (6.8) | 2.9 (9.6-33.9) | 9.0 |
| Sodium stearoyl-2-lactylate | E481 | 59.4 (67.1) | 38.9 (17.9-60.3) | 8.6 |
| Processed euchema seaweed | E407a | 18.4 (23.4) | 11.4 (4.5-4.5) | 7.9 |
| Ferrous gluconate | E579 | 1.3 (2.3) | 0.5 (9.7-80.4) | 7.3 |
| Citric acid esters of mono- and diglycerides of fatty acids | E472c | 117.0 (186.3) | 53.0 (0.8-4.5) | 7.2 |
| Titanium dioxide | E171 | 7.6 (15.0) | 3.4 (7.5-102.8) | 7.0 |
| Magnesium carbonates | E504 | 1296.6 (3120.7) | 500.0 (5.0-17.1) | 7.0 |
| Calcium sorbate | E203 | 27.3 (25.4) | 20.0 (0.5-1.5) | 6.8 |
| Riboflavin | E101 | 5.3 (8.8) | 3.2 (0.4-6.0) | 6.5 |
| Tartaric acid | E334 | 76.0 (98.5) | 44.6 (0.7-4.1) | 6.3 |
| Potassium carbonates | E501 | 898.6 (1429.3) | 350.0 (0.4-2.5) | 5.9 |
| Sorbic acid | E200 | 11.5 (15.5) | 6.6 (1.1-2.4) | 5.9 |
| Brilliant blue FCF | E133 | 0.2 (0.9) | 0.1 (1.1-4.2) | 5.6 |
| Sodium phosphates | E339 | 215.2 (296.2) | 107.1 (5.0-14.3) | 5.6 |
| Beeswax | E901 | 1.2 (2.1) | 0.5 (1.3-3.3) | 5.5 |
| Copper complexes of chlorophyllis and chlorophyllins | E141 | 2.5 (6.2) | 0.3 (4.3-19.2) | 5.3 |
| Ammonia caramel | E150c | 98.9 (262.9) | 26.8 (0.5-2.3) | 5.3 |
| Potassium phosphates | E340 | 144.9 (396.6) | 58.3 (1.5-10.4) | 5.2 |
| Sodium hydroxide | E524 | 1012.5 (1252.5) | 678.6 (0.1-0.3) | 5.0 |
| Steviol glycosides | E960 | 2.8 (4.6) | 1.5 (19.1-81.4) | 4.9 |
| Calcium disodium ethylene diamine tetraacetate | E385 | 0.3 (0.6) | 0.2 (104.6-409.3) | 4.8 |
| Polyglycerol esters of fatty acids | E475 | 180.9 (202.7) | 107.1 (43.6-251.4) | 4.5 |
| Leucine | E641 | 14.0 (18.7) | 7.9 (8.6-104.2) | 4.3 |
| Hypromellose | E464 | 66.5 (137.4) | 0.4 (9.4-28.3) | 4.2 |
| Calcium carbonate | E170 | 581.7 (905.2) | 262.8 (56.8-84.6) | 3.6 |
| Sodium benzoate | E211 | 5.1 (8.5) | 2.4 (19.8-79.0) | 3.5 |
| Fatty acid esters of ascorbic acid | E304 | 6.9 (15.0) | 1.1 (46.9-272.9) | 3.4 |
| Patent Blue v | E131 | 0.3 (1.6) | 0.0 (16.7-145.7) | 3.4 |
| Tartrazine | E102 | 0.9 (1.7) | 0.3 (45.7-269.8) | 3.4 |
| Acetic acid esters of mono- and diglycerides of fatty acids | E472a | 216.7 (483.4) | 72.3 (21.7-41.2) | 3.2 |
| Agar | E406 | 160.7 (190.9) | 104.8 (27.4-27.4) | 2.8 |
| Lysozyme | E1105 | 7.0 (10.0) | 2.9 (0.1-0.3) | 2.8 |
| Sucrose esters of fatty acids | E473 | 64.0 (86.5) | 37.5 (0.1-0.3) | 2.5 |
| Saccharin | E954 | 8.3 (42.2) | 0.0 (0.4-2.3) | 2.5 |
| Methyl cellulose | E461 | 90.1 (93.8) | 61.8 (2.2-9.0) | 2.4 |
| Silicon dioxide | E551 | 212.4 (968.6) | 16.5 (13.2-61.7) | 2.4 |
| Nisin | E234 | 0.1 (0.1) | 0.0 (8.3-26.7) | 2.3 |
| Triethyl citrate | E1505 | 16.8 (19.3) | 10.4 (15.8-29.9) | 2.1 |
| Gellan gum | E418 | 19.6 (24.7) | 11.9 (8.4-21.9) | 2.1 |
| Beta-apo-8’-carotenal | E160e | 2.3 (4.3) | 0.9 (47.6-212.8) | 2.1 |
| Caramel color | E150 | 117.7 (169.8) | 67.6 (18.2-121.7) | 2.0 |
| Maltitol | E965 | 318.6 (600.3) | 103.7 (6.1-21.6) | 1.9 |
| Propane-1,2-diol esters of acids | E477 | 44.2 (41.1) | 31.1 (22.8-85.5) | 1.8 |
| Iron oxides | E172 | 8.1 (17.7) | 3.7 (67.3-309.5) | 1.7 |
| Polydextrose | E1200 | 1666.6 (2034.7) | 1014.7 (61.3-514.3) | 1.6 |
| Sunset Yellow FCF | E110 | 0.1 (0.5) | 0.0 (26.9-215.9) | 1.6 |
| Isomaltitol | E953 | 628.4 (1959.1) | 193.5 (2.0-5.2) | 1.5 |
| Butylated hydroxyanisole | E320 | 8.0 (9.0) | 5.7 (5.1-24.3) | 1.4 |
| Glycerol esters of wood rosins | E445 | 5.7 (8.1) | 3.6 (54.0-226.8) | 1.4 |
| Erythritol | E968 | 36.4 (128.9) | 3.4 (17.1-44.5) | 1.3 |
| Disodium ethylene diamine tetraacetate | E386 | 0.2 (0.2) | 0.2 (180.0-716.1) | 1.3 |
| Mono- and diacetyltartaric esters of mono- and diglycerides of fatty acids | E472 | 263.1 (236.4) | 185.7 (68.5-176.8) | 1.2 |
| Butylated hydroxytoluene | E321 | 0.4 (0.6) | 0.2 (8.8-27.2) | 1.2 |
| Xylitol | E967 | 185.6 (244.9) | 104.1 (8.9-31.5) | 1.2 |
| Caustic sulphite caramel | E150b | 7.9 (15.4) | 2.4 (72.7-321.8) | 1.1 |
| Polyoxyethene (20) sorbitan monooleate | E433 | 26.6 (35.5) | 14.3 (2.7-12.9) | 1.0 |
| Sodium nitrate | E251 | 0.3 (0.6) | 0.0 (6.5-20.4) | 0.9 |
| Cyclamate | E952 | 8.2 (12.6) | 4.3 (1.7-7.1) | 0.9 |
| Potassium alginate | E402 | 23.8 (26.6) | 15.9 (166.2-704.8) | 0.8 |
| Potassium nitrite | E249 | 0.1 (0.1) | 0.1 (35.9-154.0) | 0.8 |
| Propyl gallate | E310 | 11.8 (11.4) | 7.6 (22.3-199.8) | 0.7 |
| Sodium proprionate | E281 | 33.1 (52.3) | 17.1 (2.7-131.0) | 0.7 |
| Natamycin | E235 | 0.0 (0.0) | 0.0 (36.2-123.4) | 0.7 |
| Erythrosine | E127 | 3.4 (4.2) | 2.3 (0.1-76.5) | 0.6 |
| Potassium benzoate | E212 | 10.1 (12.9) | 5.9 (6.1-42.7) | 0.5 |
| DL-Alpha-tocopherol | E307c | 3.4 (4.2) | 2.1 (2.1-11.4) | 0.5 |
| Sorbitan tristearate | E492 | 73.1 (127.5) | 13.8 (64.4-300.8) | 0.5 |
| Potassium hydroxide | E525 | 40.9 (31.5) | 30.0 (111.4-371.4) | 0.4 |
| Ponceau 4R | E124 | 0.2 (0.1) | 0.1 (41.5-160.9) | 0.4 |
| Magnesium oxide | E530 | 619.1 (577.9) | 416.7 (28.6-234.5) | 0.3 |
| Neohesperidine dihydrochalcone | E959 | 1.6 (1.6) | 1.1 (21.6-139.3) | 0.3 |
| Shellac | E904 | 0.7 (2.1) | 0.0 (10.6-41.6) | 0.3 |
| Hexamine | E239 | 0.0 (0.1) | 0.0 (20.1-75.0) | 0.3 |
| Aspartame-acesulfame salt | E962 | 1.2 (1.5) | 0.7 (57.1-226.2) | 0.3 |
| Quinoline Yellow | E104 | 0.3 (0.4) | 0.2 (5.4-28.6) | 0.3 |
| Green S | E142 | 3.8 (8.4) | 1.7 (19.6-55.0) | 0.3 |
| Calcium stearoyl-2-lactylate | E482 | 47.8 (51.5) | 28.6 (19.0-74.3) | 0.2 |
| Sodium aluminosilicate | E554 | 0.5 (0.5) | 0.4 (17.1-64.3) | 0.2 |
| Propionic acid | E280 | 4.1 (4.1) | 2.8 (5.7-16.5) | 0.2 |
| Indigo Carmine | E132 | 0.7 (2.5) | 0.3 (4.6-96.8) | 0.2 |
| Crosslinked sodium carboxymethyl cellulose | E468 | 9.8 (15.7) | 5.3 (509.9-2700.0) | 0.1 |
| Sodium ferrocyanide | E535 | 0.4 (0.5) | 0.3 (125.0-1112.2) | 0.1 |
| Sucrose acetate isobutyrate | E444 | 15.7 (16.9) | 10.4 (160.0-900.0) | 0.1 |
| Sorbitan monostearate | E491 | 17.3 (43.2) | 7.4 (171.7-1250.0) | 0.1 |
| Calcium alginate | E404 | 31.5 (50.9) | 21.6 (1.8-1.8) | 0.1 |
| Calcium ascorbate | E302 | 1.2 (1.4) | 0.8 (156.3-1297.7) | 0.1 |
| Talc | E553b | 34.9 (28.6) | 24.2 (21.2-50.4) | 0.1 |
| Erythorbic acid | E315 | 2.7 (2.1) | 2.1 (240.0-815.4) | 0.1 |
| Alginic acid | E400 | 7.1 (7.4) | 4.6 (0.0-0.5) | 0.1 |
| Thaumatin | E957 | 0.0 (0.0) | 0.0 (1.3-6.0) | 0.1 |
| Polyoxyethene (20) sorbitan monostearate | E435 | 25.5 (31.6) | 17.5 (0.5-100.0) | 0.0 |
| Dimethyl dicarbonate | E242 | 19.8 (28.2) | 9.9 (20.8-49.5) | 0.0 |
| Allura Red 4C | E129 | 1.0 (0.9) | 1.0 (17.9-44.6) | 0.0 |
| Quillaia extract | E999 | 7.4 (4.1) | 6.0 (0.2-0.7) | 0.0 |
| Sodium tartrates | E335 | 20.7 (16.0) | 19.2 (0.2-1.2) | 0.0 |
| Tert-butylhydroquinone | E319 | 2.5 (4.1) | 0.7 (0.1-0.2) | 0.0 |
| Magnesium silicate | E553 | 40.0 (38.2) | 21.7 (10.1-415.6) | 0.0 |
| Brillant Black | E151 | 0.5 (0.4) | 0.3 (2.7-2.7) | 0.0 |
| Lycopene | E160d | 0.4 (0.3) | 0.4 (3.3-17.8) | 0.0 |
| Benzoic acid | E210 | 8.9 (6.6) | 9.1 (0.0-0.1) | 0.0 |
| Dimethyl polysiloxane | E900 | 0.0 (0.1) | 0.0 (0.2-1.3) | 0.0 |
| Fumaric acid | E297 | 2.9 (2.8) | 1.6 (0.7-4.2) | 0.0 |
| Mannitol | E421 | 35.4 (23.4) | 29.1 (0.0-0.5) | 0.0 |
| Carmoisine | E122 | 0.4 (0.4) | 0.3 (1047.6-1047.6) | 0.0 |
| Brown HT | E155 | 1.5 (0.9) | 1.2 (1.6-1.6) | 0.0 |
| Sodium aluminium phosphate | E541 | 3.8 (3.7) | 1.9 (1.5-14.4) | 0.0 |
| Propylene glycol | E1520 | 32.4 (28.3) | 34.1 (5.3-38.6) | 0.0 |
| Concentrated tocopherol | E307b | 1.8 (1.1) | 1.7 (1.4-10.0) | 0.0 |
| Magnesium phosphates | E343 | 30.8 (19.6) | 33.5 (94.9-517.8) | 0.0 |
| Konjac gum | E425 | 121.8 (108.4) | 125.0 (0.0-0.3) | 0.0 |
| Amaranth | E123 | 0.2 (0.1) | 0.2 (1.4-6.7) | 0.0 |
| Potassium sodium tartrate | E337 | 70.7 (39.4) | 70.7 (0.0-0.0) | 0.0 |
| Propane-1,2-diol alginate | E405 | 15.2 (19.0) | 15.2 (0.6-2.1) | 0.0 |
| Karaya gum | E416 | 3.6 (4.5) | 3.6 (0.7-3.2) | 0.0 |
| Glutamic acid | E620 | 0.1 (0.1) | 0.1 (0.5-1.4) | 0.0 |
| Sodium tetraborate | E285 | 4.5 (0.0) | 4.5 (52.9-311.2) | 0.0 |
| Succinic acid | E363 | 27.4 (0.0) | 27.4 (51.8-211.7) | 0.0 |
| Aluminium sodium sulfate | E521 | 1.8 (0.0) | 1.8 (0.9-12.0) | 0.0 |
| Ethyl maltol | E637 | 2.7 (0.0) | 2.7 (4.5-11.0) | 0.0 |
| Carbamic acid derivatives | E927 | 1047.6 (0.0) | 1047.6 (614.6-1920.6) | 0.0 |
| Azodicarbonamide | E927a | 1.6 (0.0) | 1.6 (0.8-6.2) | 0.0 |

^a^The 75 food additives highlighted in grey represent those consumed by at least 5% of the NutriNet-Santé cohort’s participants and were therefore included in NMF analyses.

^b^Data are mean (standard deviation (SD)), median (interquartile range (IQR)) in mg/day, or %. All food additive intake data in this table are calculated as the mean intake during the first two years of participation in the study.

## Table B. Food additive mixtures identified by non-negative matrix factorisation^a^

| **Food additives** | **Mixtures** | | | | |
| --- | --- | --- | --- | --- | --- |
|  | **1** | **2** | **3** | **4** | **5** |
| E100 Curcumin | 0.01 | 0.16 | 0 | 0.01 | 0.07 |
| E101 Riboflavin | 0.07 | 0.02 | 0.53 | 0.01 | 0.05 |
| E120 Cochineal, Carminic acid, Carmines | 0.02 | 0.09 | 0 | 0.01 | 0.09 |
| E133 Brilliant blue FCF | 0.01 | 0 | 0.02 | 0 | 0.03 |
| E141 Copper complexes of chlorophyllis and chlorophyllins | 0.01 | 0.02 | 0 | 0.01 | 0.04 |
| E150a Plain caramel | 0.02 | 0.08 | 0.1 | 0.02 | 0.05 |
| E150c Ammonia caramel | 0 | 0.1 | 0.02 | 0.01 | 0.03 |
| E150d Sulphite ammonia caramel | 0.03 | -0.01 | 0.03 | 0.03 | 0.59 |
| E160 Carotene | 0.02 | 0.01 | 0.02 | 0.02 | 0.08 |
| E160a Carotenes | 0.01 | 0.03 | 0 | 0.01 | 0.11 |
| E160b Annatto, Bixin, Norbixin | 0.04 | 0.09 | 0 | 0.04 | 0.05 |
| E160c Paprika extract, capsanthin, capsorubin | 0.05 | 0.11 | 0 | 0.02 | 0.17 |
| E161b Lutein | 0.02 | 0.13 | 0 | 0.01 | 0.14 |
| E162 Beetroot Red, betanin | 0.02 | 0.05 | 0 | 0.01 | 0.07 |
| E163 Anthocyanins | 0.02 | 0.06 | 0.01 | 0.02 | 0.15 |
| E171 Titanium dioxide | 0.02 | 0.01 | 0 | 0.04 | 0.1 |
| E200 Sorbic acid | 0.05 | 0.02 | 0 | 0.05 | 0.01 |
| E202 Potassium sorbate | 0.06 | 0.17 | 0.01 | 0.03 | 0.09 |
| E203 Calcium sorbate | -0.01 | 0.01 | 0 | 0 | 0 |
| E250 Sodium nitrite | 0.05 | 0.1 | 0.01 | 0.02 | 0.1 |
| E252 Potassium nitrate | 0.03 | -0.02 | 0.01 | 0.01 | 0.05 |
| E260 Acetic acid | 0.02 | 0.1 | 0 | 0.01 | 0.04 |
| E262 Sodium acetate | 0.02 | 0.05 | 0 | 0.01 | 0.02 |
| E270 Lactic acid | 0.01 | 0.04 | 0 | 0 | 0.06 |
| E282 Calcium propionate | 0.04 | 0.05 | 0 | 0.02 | 0.07 |
| E296 Malic acid | 0.08 | 0.01 | 0.01 | 0.03 | 0.19 |
| E300 Ascorbic acid | 0.11 | 0.06 | 0 | 0.03 | 0.06 |
| E301 Sodium ascorbate | 0.06 | 0.04 | 0.01 | 0.02 | 0.06 |
| E306 Tocopherols | 0.03 | 0.04 | 0.02 | 0.04 | 0.05 |
| E307 Alpha-tocopherol | 0.11 | 0.04 | 0.24 | 0.28 | 0.02 |
| E316 Sodium erythorbate | 0.05 | 0.1 | 0 | 0.01 | 0.06 |
| E322 Lecithins | 0.11 | 0.04 | 0.09 | 0.15 | 0.08 |
| E330 Citric acid | 0.13 | 0.09 | 0.1 | 0.04 | 0.83 |
| E331 Sodium citrates | 0.04 | 0.1 | 0.12 | 0.03 | 0.63 |
| E334 Tartaric acid | 0.01 | 0.06 | 0 | 0 | 0.01 |
| E338 Phosphoric acid | 0.03 | -0.01 | 0.03 | 0.03 | 0.59 |
| E339 Sodium phosphates | 0.02 | 0.08 | 0.01 | 0.01 | 0.03 |
| E340 Potassium phosphates | 0.02 | 0.02 | 0 | 0 | 0.03 |
| E341 Calcium phosphates | 0.07 | 0.07 | 0.05 | 0.08 | 0.02 |
| E392 Carnosic acid (Extracts of rosemary) | 0.01 | 0.02 | 0 | 0.02 | 0.04 |
| E401 Sodium alginate | 0.08 | 0.04 | 0.01 | 0.02 | 0.04 |
| E407 Carrageenan | 0.06 | 0.24 | 0.01 | 0.04 | 0.08 |
| E407a Processed euchema seaweed | 0.01 | 0.04 | 0 | 0.01 | 0.04 |
| E410 Locust bean gum | 0.04 | 0.02 | 0.01 | 0.02 | 0.07 |
| E412 Guar gum | 0.03 | 0.26 | 0.01 | 0.01 | 0.15 |
| E414 Arabic gum | 0.02 | 0 | 0.01 | 0.03 | 0.23 |
| E415 Xanthan gum | 0.05 | 0.16 | 0.01 | 0.02 | 0.11 |
| E420 Sorbitols | 0.16 | 0.02 | 0.01 | 0.08 | 0.09 |
| E422 Glycerol | 0.37 | 0.05 | 0.01 | 0.1 | 0.08 |
| E440 Pectins | 0.13 | 0.31 | 0 | 0.02 | 0.15 |
| E442 Ammonium phosphatides | 0.01 | -0.01 | 0 | 0.01 | 0.03 |
| E450 Diphosphates | 0.78 | 0.07 | 0.07 | 0.3 | 0.08 |
| E451 Triphosphates | 0.03 | 0.12 | 0 | 0.01 | 0.05 |
| E452 Polyphosphates | 0.01 | 0.21 | 0 | 0 | 0.06 |
| E460 Cellulose (microcrystalline cellulose) | 0.06 | 0.06 | 0.01 | 0.08 | 0.04 |
| E466 Carboxy methyl cellulose | 0.02 | 0.04 | 0.03 | 0.02 | 0.04 |
| E471 Mono- and diglycerides of fatty acids | 0.1 | 0.13 | 0 | 0.08 | 0.13 |
| E472b Lactic acid esters of mono- and diglycerides of fatty acids | 0.03 | 0.05 | 0.01 | 0.03 | 0.03 |
| E472c Citric acid esters of mono- and diglycerides of fatty acids | 0.01 | 0.11 | 0.01 | 0 | 0.02 |
| E472e Mono and diacetyl tartaric acid esters of mono and diglycerides of fatty acids | 0.07 | 0.01 | 0.11 | 0.18 | 0.04 |
| E476 Polyglycerol polyricinoleate | 0.03 | 0.05 | 0 | 0.09 | 0.03 |
| E481 Sodium stearoyl-2-lactylate | 0.02 | 0.01 | 0 | 0.02 | 0.04 |
| E500 Sodium carbonates | 0.99 | 0.03 | 0.14 | 0.35 | 0.05 |
| E501 Potassium carbonates | 0.17 | 0 | 0 | 0 | 0 |
| E503 Ammonium carbonates | 0.35 | 0.01 | 0.17 | 0.99 | 0.01 |
| E504 Magnesium carbonates | 0.14 | -0.01 | 0.99 | 0.17 | 0.04 |
| E579 Ferrous gluconate | 0.02 | 0.02 | 0 | 0.01 | 0.02 |
| E621 Monosodium glutamate | 0.01 | 0.12 | 0 | -0.01 | 0.04 |
| E901 Beeswax | 0.02 | 0 | 0 | 0.02 | 0.09 |
| E903 Carnauba wax | 0.03 | 0 | 0.01 | 0.03 | 0.18 |
| E950 Acesulfame K | 0.02 | 0.08 | 0.04 | 0.02 | 0.56 |
| E951 Aspartame | 0 | 0.06 | 0.02 | 0 | 0.41 |
| E955 Sucralose | 0.01 | 0.01 | 0.03 | 0 | 0.25 |
| Food additive sulphites | 0.01 | -0.01 | -0.01 | -0.02 | -0.02 |
| Modified starches | 0.05 | 0.99 | 0 | 0.01 | 0.03 |

^a^Food additive mixtures were identified using non-negative matrix factorisation (NMF) based on the algorithm developed by Lee and Seung^17^. The non-negative matrix factorisation creates linear combinations of the initial set of variables. with the aim to group those that are correlated while explaining as much variation from the dataset as possible. NMF was performed on the 75 food additives that were consumed by at least 5% of the study population. Five main NMF components (i.e. the five food additive mixtures) were identified. This table provides the correlation matrix (Spearman) between each NMF component and each food additive intake. Food additives with loading values ≥│0.15│were considered as the most emblematic of each mixture for description purposes, though all additives contributed to the mixture score calculation. The variance explained by this model was 89% of the total variance, as calculated by determining the reconstruction error of the original matrix.

## Table C. Consensus map for rank number determination and sensitivity analyses using other decomposition algorithms in the NMF procedure

### C. a. Rank selection in NMF (consensus map)


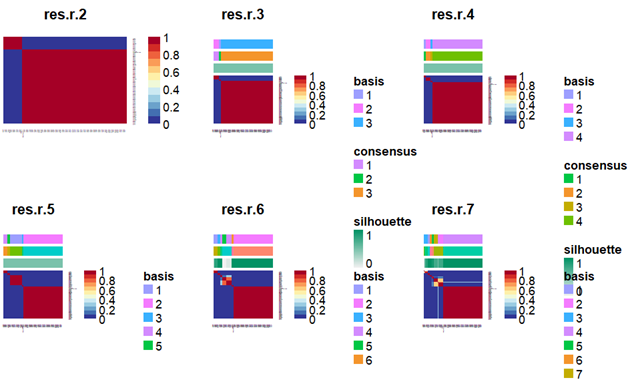


The consensus map provides a visual summary of the NMF analysis results, highlighting the stability of components across multiple iterations of the process^21^. The heatmap represents the pairwise homogeneity of samples within the same component, with values ranging from 0 (indicating that the samples are never grouped together) to 1 (indicating that the samples are always grouped together). Red squares denote strong agreement among samples, whereas blue squares signify weak or no agreement.

Components assignments are depicted by the accompanying colour bar, with each colour representing a distinct component. The consensus values quantify component stability, where higher values indicate more robust and well-defined components. Additionally, the silhouette metric evaluates clustering quality by assessing the separation between components and minimizing overlap.

###

### C. b. Food additive mixtures identified by non-negative matrix factorisation - Brunet algorithm: loading values of main additive contributors, NutriNet-Santé cohort, 2009-2023^a^

| **Food additives** | **Mixtures** | | | | |
| --- | --- | --- | --- | --- | --- |
|  | **1** | **2** | **3** | **4** | **5** |
| E100 Curcumin | 0.01 | 0.20 | 0.00 | 0.01 | 0.05 |
| E101 Riboflavin | 0.06 | 0.04 | 0.53 | 0.01 | 0.05 |
| E120 Cochineal, Carminic acid, Carmines | 0.02 | 0.11 | 0.00 | 0.01 | 0.07 |
| E133 Brilliant blue FCF | 0.01 | 0.00 | 0.02 | 0.00 | 0.06 |
| E141 Copper complexes of chlorophyllis and chlorophyllins | 0.01 | 0.03 | 0.00 | 0.01 | 0.04 |
| E150a Plain caramel | 0.03 | 0.10 | 0.10 | 0.03 | 0.05 |
| E150c Ammonia caramel | 0.00 | 0.11 | 0.02 | 0.04 | 0.12 |
| E150d Sulphite ammonia caramel | 0.03 | -0.02 | 0.03 | 0.03 | 0.73 |
| E160 Carotene | 0.03 | 0.04 | 0.02 | 0.02 | 0.10 |
| E160a Carotenes | 0.02 | 0.07 | 0.00 | 0.01 | 0.09 |
| E160b Annatto, Bixin, Norbixin | 0.04 | 0.11 | 0.00 | 0.03 | 0.03 |
| E160c Paprika extract, capsanthin, capsorubin | 0.05 | 0.17 | 0.00 | 0.02 | 0.16 |
| E161b Lutein | 0.02 | 0.17 | 0.00 | 0.02 | 0.12 |
| E162 Beetroot Red, betanin | 0.02 | 0.10 | 0.00 | 0.01 | 0.03 |
| E163 Anthocyanins | 0.03 | 0.08 | 0.01 | 0.03 | 0.14 |
| E171 Titanium dioxide | 0.02 | 0.01 | 0.00 | 0.04 | 0.22 |
| E200 Sorbic acid | 0.06 | 0.02 | 0.00 | 0.05 | 0.01 |
| E202 Potassium sorbate | 0.07 | 0.32 | 0.01 | 0.03 | 0.07 |
| E203 Calcium sorbate | -0.01 | 0.01 | 0.00 | 0.00 | 0.00 |
| E250 Sodium nitrite | 0.05 | 0.18 | 0.01 | 0.02 | 0.06 |
| E252 Potassium nitrate | 0.03 | 0.00 | 0.01 | 0.01 | 0.04 |
| E260 Acetic acid | 0.02 | 0.16 | 0.00 | 0.01 | 0.04 |
| E262 Sodium acetate | 0.02 | 0.08 | 0.00 | 0.01 | 0.02 |
| E270 Lactic acid | 0.01 | 0.08 | 0.00 | 0.00 | 0.07 |
| E282 Calcium propionate | 0.05 | 0.12 | 0.00 | 0.02 | 0.06 |
| E296 Malic acid | 0.09 | 0.01 | 0.01 | 0.03 | 0.20 |
| E300 Ascorbic acid | 0.12 | 0.11 | 0.00 | 0.04 | 0.05 |
| E301 Sodium ascorbate | 0.05 | 0.06 | 0.01 | 0.01 | 0.05 |
| E306 Tocopherols | 0.03 | 0.07 | 0.02 | 0.05 | 0.12 |
| E307 Alpha-tocopherol | 0.10 | 0.04 | 0.24 | 0.27 | 0.03 |
| E316 Sodium erythorbate | 0.04 | 0.15 | 0.00 | 0.01 | 0.03 |
| E322 Lecithins | 0.12 | 0.07 | 0.09 | 0.15 | 0.20 |
| E330 Citric acid | 0.14 | 0.15 | 0.10 | 0.04 | 0.51 |
| E331 Sodium citrates | 0.04 | 0.12 | 0.12 | 0.04 | 0.71 |
| E334 Tartaric acid | 0.01 | 0.09 | 0.00 | 0.00 | 0.00 |
| E338 Phosphoric acid | 0.03 | -0.02 | 0.03 | 0.02 | 0.73 |
| E339 Sodium phosphates | 0.03 | 0.16 | 0.01 | 0.02 | 0.05 |
| E340 Potassium phosphates | 0.02 | 0.02 | 0.01 | 0.00 | 0.11 |
| E341 Calcium phosphates | 0.07 | 0.06 | 0.04 | 0.39 | 0.01 |
| E392 Carnosic acid (Extracts of rosemary) | 0.02 | 0.05 | 0.00 | 0.02 | 0.03 |
| E401 Sodium alginate | 0.09 | 0.07 | 0.01 | 0.04 | 0.04 |
| E407 Carrageenan | 0.06 | 0.31 | 0.01 | 0.05 | 0.07 |
| E407a Processed euchema seaweed | 0.01 | 0.09 | 0.00 | 0.01 | 0.05 |
| E410 Locust bean gum | 0.05 | 0,12 | 0,01 | 0,02 | 0,05 |
| E412 Guar gum | 0.03 | 0,47 | 0,01 | 0,01 | 0,07 |
| E414 Arabic gum | 0.02 | -0,01 | 0,01 | 0,02 | 0,47 |
| E415 Xanthan gum | 0.05 | 0,35 | 0,01 | 0,02 | 0,04 |
| E420 Sorbitols | 0.20 | 0,03 | 0,01 | 0,07 | 0,20 |
| E422 Glycerol | 0.49 | 0,04 | 0,01 | 0,11 | 0,04 |
| E440 Pectins | 0.13 | 0.43 | 0.00 | 0.03 | 0.06 |
| E442 Ammonium phosphatides | 0.01 | 0.01 | 0.00 | 0.01 | 0.03 |
| E450 Diphosphates | 0.82 | 0.11 | 0.07 | 0.29 | 0.04 |
| E451 Triphosphates | 0.03 | 0.27 | 0.00 | 0.01 | 0.04 |
| E452 Polyphosphates | 0.01 | 0.35 | 0.00 | 0.02 | 0.02 |
| E460 Cellulose (microcrystalline cellulose) | 0.06 | 0.11 | 0.01 | 0.08 | 0.07 |
| E466 Carboxy methyl cellulose | 0.02 | 0.06 | 0.03 | 0.02 | 0.17 |
| E471 Mono- and diglycerides of fatty acids | 0.12 | 0.27 | 0.00 | 0.08 | 0.08 |
| E472b Lactic acid esters of mono- and diglycerides of fatty acids | 0.03 | 0.10 | 0.01 | 0.04 | 0.04 |
| E472c Citric acid esters of mono- and diglycerides of fatty acids | 0.02 | 0.18 | 0.01 | 0.02 | 0.05 |
| E472e Mono and diacetyl tartaric acid esters of mono and diglycerides of fatty acids | 0.07 | 0.04 | 0.12 | 0.17 | 0.04 |
| E476 Polyglycerol polyricinoleate | 0.03 | 0.07 | 0.00 | 0.15 | 0.02 |
| E481 Sodium stearoyl-2-lactylate | 0.02 | 0.05 | 0.00 | 0.02 | 0.04 |
| E500 Sodium carbonates | 0.97 | 0.02 | 0.14 | 0.34 | 0.02 |
| E501 Potassium carbonates | 0.27 | 0.00 | 0.00 | 0.00 | 0.00 |
| E503 Ammonium carbonates | 0.33 | -0.01 | 0.17 | 0.95 | 0.03 |
| E504 Magnesium carbonates | 0.11 | -0.01 | 1.00 | 0.17 | 0.04 |
| E579 Ferrous gluconate | 0.02 | 0.06 | 0.00 | 0.00 | 0.01 |
| E621 Monosodium glutamate | 0.01 | 0.27 | 0.00 | -0.01 | -0.01 |
| E901 Beeswax | 0.03 | 0.00 | 0.00 | 0.02 | 0.09 |
| E903 Carnauba wax | 0.03 | 0.00 | 0.01 | 0.03 | 0.26 |
| E950 Acesulfame K | 0.02 | 0.09 | 0.04 | 0.02 | 0.67 |
| E951 Aspartame | 0.00 | 0.07 | 0.02 | 0.00 | 0.50 |
| E955 Sucralose | 0.01 | 0.04 | 0.03 | 0.00 | 0.19 |
| Modified starches | 0.04 | 0.89 | 0.00 | 0.03 | 0.00 |
| Food additive sulphites | 0.00 | 0.02 | -0.01 | -0.02 | -0.04 |

^a^Food additive mixtures were identified using non-negative matrix factorisation (NMF) based on the algorithm based on Kullback-Leibler divergence^18^. The non-negative matrix factorisation creates linear combinations of the initial set of variables. with the aim to group those that are correlated while explaining as much variation from the dataset as possible. NMF was performed on the 75 food additives that were consumed by at least 5% of the study population. Five main NMF components (i.e. the five food additive mixtures) were identified. This table provides the correlation matrix (Spearman) between each NMF component and each food additive intake. Food additives with loading values ≥│0.15│were considered as the most emblematic of each mixture for description purposes, though all additives contributed to the mixture score calculation. The variance explained by this model was 85% of the total variance, as calculated by determining the reconstruction error of the original matrix.

### C. c. Food additive mixtures identified by Non-smooth non-negative matrix factorisation (nsNMF): loading values of main additive contributors, NutriNet-Santé cohort, 2009-2023^a^

| **Food additives** | **Mixtures** | | | | |
| --- | --- | --- | --- | --- | --- |
|  | **1** | **2** | **3** | **4** | **5** |
| E100 Curcumin | 0.01 | 0.20 | 0.00 | 0.01 | 0.05 |
| E101 Riboflavin | 0.06 | 0.04 | 0.53 | 0.01 | 0.05 |
| E120 Cochineal, Carminic acid, Carmines | 0.02 | 0.11 | 0.00 | 0.01 | 0.07 |
| E133 Brilliant blue FCF | 0.01 | 0.00 | 0.02 | 0.00 | 0.06 |
| E141 Copper complexes of chlorophyllis and chlorophyllins | 0.01 | 0.03 | 0.00 | 0.01 | 0.04 |
| E150a Plain caramel | 0.03 | 0.10 | 0.10 | 0.03 | 0.05 |
| E150c Ammonia caramel | 0.00 | 0.11 | 0.02 | 0.04 | 0.12 |
| E150d Sulphite ammonia caramel | 0.03 | -0.02 | 0.03 | 0.03 | 0.73 |
| E160 Carotene | 0.03 | 0.04 | 0.02 | 0.02 | 0.10 |
| E160a Carotenes | 0.02 | 0.07 | 0.00 | 0.01 | 0.09 |
| E160b Annatto, Bixin, Norbixin | 0.04 | 0.11 | 0.00 | 0.03 | 0.03 |
| E160c Paprika extract, capsanthin, capsorubin | 0.05 | 0.17 | 0.00 | 0.02 | 0.16 |
| E161b Lutein | 0.02 | 0.17 | 0.00 | 0.02 | 0.12 |
| E162 Beetroot Red, betanin | 0.02 | 0.10 | 0.00 | 0.01 | 0.03 |
| E163 Anthocyanins | 0.03 | 0.08 | 0.01 | 0.03 | 0.14 |
| E171 Titanium dioxide | 0.02 | 0.01 | 0.00 | 0.04 | 0.22 |
| E200 Sorbic acid | 0.06 | 0.02 | 0.00 | 0.05 | 0.01 |
| E202 Potassium sorbate | 0.07 | 0.32 | 0.01 | 0.03 | 0.07 |
| E203 Calcium sorbate | -0.01 | 0.01 | 0.00 | 0.00 | 0.00 |
| E250 Sodium nitrite | 0.05 | 0.18 | 0.01 | 0.02 | 0.06 |
| E252 Potassium nitrate | 0.03 | 0.00 | 0.01 | 0.01 | 0.04 |
| E260 Acetic acid | 0.02 | 0.16 | 0.00 | 0.01 | 0.04 |
| E262 Sodium acetate | 0.02 | 0.08 | 0.00 | 0.01 | 0.02 |
| E270 Lactic acid | 0.01 | 0.08 | 0.00 | 0.00 | 0.07 |
| E282 Calcium propionate | 0.05 | 0.12 | 0.00 | 0.02 | 0.06 |
| E296 Malic acid | 0.09 | 0.01 | 0.01 | 0.03 | 0.20 |
| E300 Ascorbic acid | 0.12 | 0.11 | 0.00 | 0.04 | 0.05 |
| E301 Sodium ascorbate | 0.05 | 0.06 | 0.01 | 0.01 | 0.05 |
| E306 Tocopherols | 0.03 | 0.07 | 0.02 | 0.05 | 0.12 |
| E307 Alpha-tocopherol | 0.10 | 0.04 | 0.24 | 0.27 | 0.03 |
| E316 Sodium erythorbate | 0.04 | 0.15 | 0.00 | 0.01 | 0.03 |
| E322 Lecithins | 0.12 | 0.07 | 0.09 | 0.15 | 0.20 |
| E330 Citric acid | 0.14 | 0.15 | 0.10 | 0.04 | 0.51 |
| E331 Sodium citrates | 0.04 | 0.12 | 0.12 | 0.04 | 0.71 |
| E334 Tartaric acid | 0.01 | 0.09 | 0.00 | 0.00 | 0.00 |
| E338 Phosphoric acid | 0.03 | -0.02 | 0.03 | 0.02 | 0.73 |
| E339 Sodium phosphates | 0.03 | 0.16 | 0.01 | 0.02 | 0.05 |
| E340 Potassium phosphates | 0.02 | 0.02 | 0.01 | 0.00 | 0.11 |
| E341 Calcium phosphates | 0.07 | 0.06 | 0.04 | 0.39 | 0.01 |
| E392 Carnosic acid (Extracts of rosemary) | 0.02 | 0.05 | 0.00 | 0.02 | 0.03 |
| E401 Sodium alginate | 0.09 | 0.07 | 0.01 | 0.04 | 0.04 |
| E407 Carrageenan | 0.06 | 0.31 | 0.01 | 0.05 | 0.07 |
| E407a Processed euchema seaweed | 0.01 | 0.09 | 0.00 | 0.01 | 0.05 |
| E410 Locust bean gum | 0.05 | 0.12 | 0.01 | 0.02 | 0.05 |
| E412 Guar gum | 0.03 | 0.47 | 0.01 | 0.01 | 0.07 |
| E414 Arabic gum | 0.02 | -0.01 | 0.01 | 0.02 | 0.47 |
| E415 Xanthan gum | 0.05 | 0.35 | 0.01 | 0.02 | 0.04 |
| E420 Sorbitols | 0.20 | 0.03 | 0.01 | 0.07 | 0.20 |
| E422 Glycerol | 0.49 | 0.04 | 0.01 | 0.11 | 0.04 |
| E440 Pectins | 0.13 | 0.43 | 0.00 | 0.03 | 0.06 |
| E442 Ammonium phosphatides | 0.01 | 0.01 | 0.00 | 0.01 | 0.03 |
| E450 Diphosphates | 0.82 | 0.11 | 0.07 | 0.29 | 0.04 |
| E451 Triphosphates | 0.03 | 0.27 | 0.00 | 0.01 | 0.04 |
| E452 Polyphosphates | 0.01 | 0.35 | 0.00 | 0.02 | 0.02 |
| E460 Cellulose (microcrystalline cellulose) | 0.06 | 0.11 | 0.01 | 0.08 | 0.07 |
| E466 Carboxy methyl cellulose | 0.02 | 0.06 | 0.03 | 0.02 | 0.17 |
| E471 Mono- and diglycerides of fatty acids | 0.12 | 0.27 | 0.00 | 0.08 | 0.08 |
| E472b Lactic acid esters of mono- and diglycerides of fatty acids | 0.03 | 0.10 | 0.01 | 0.04 | 0.04 |
| E472c Citric acid esters of mono- and diglycerides of fatty acids | 0.02 | 0.18 | 0.01 | 0.02 | 0.05 |
| E472e Mono and diacetyl tartaric acid esters of mono and diglycerides of fatty acids | 0.07 | 0.04 | 0.12 | 0.17 | 0.04 |
| E476 Polyglycerol polyricinoleate | 0.03 | 0.07 | 0.00 | 0.15 | 0.02 |
| E481 Sodium stearoyl-2-lactylate | 0.02 | 0.05 | 0.00 | 0.02 | 0.04 |
| E500 Sodium carbonates | 0.97 | 0.02 | 0.14 | 0.34 | 0.02 |
| E501 Potassium carbonates | 0.27 | 0.00 | 0.00 | 0.00 | 0.00 |
| E503 Ammonium carbonates | 0.33 | -0.01 | 0.17 | 0.95 | 0.03 |
| E504 Magnesium carbonates | 0.11 | -0.01 | 1.00 | 0.17 | 0.04 |
| E579 Ferrous gluconate | 0.02 | 0.06 | 0.00 | 0.00 | 0.01 |
| E621 Monosodium glutamate | 0.01 | 0.27 | 0.00 | -0.01 | -0.01 |
| E901 Beeswax | 0.03 | 0.00 | 0.00 | 0.02 | 0.09 |
| E903 Carnauba wax | 0.03 | 0.00 | 0.01 | 0.03 | 0.26 |
| E950 Acesulfame K | 0.02 | 0.09 | 0.04 | 0.02 | 0.67 |
| E951 Aspartame | 0.00 | 0.07 | 0.02 | 0.00 | 0.50 |
| E955 Sucralose | 0.01 | 0.04 | 0.03 | 0.00 | 0.19 |
| Modified starches | 0.04 | 0.89 | 0.00 | 0.03 | 0.00 |
| Food additive sulphites | 0.00 | 0.02 | -0.01 | -0.02 | -0.04 |

^a^ Food additive mixtures were identified using a non-smooth nonnegative matrix factorisation (nsNMF), which optimizes a clear cost function designed to explicitly represent sparsity^19^. NMF was performed on the 75 food additives that were consumed by at least 5% of the study population. Five main NMF components (i.e. the five food additive mixtures) were identified. This table provides the correlation matrix (Spearman) between each NMF component and each food additive intake. Food additives with loading values ≥│0.15│were considered as the most emblematic of each mixture for description purposes, though all additives contributed to the mixture score calculation. The variance explained by this model was 62.6% of the total variance, as calculated by determining the reconstruction error of the original matrix.

### C. d. Stability of the NMF food additive mixtures across time

#### NMF performed on mean daily food additive intakes in the 2009-2016 period

|  | **Mixtures** | | | | | |
| --- | --- | --- | --- | --- | --- | --- |
| **Food additives** | **1** | **2** | **3** | **4** | **5** |  |
| E100 Curcumin | 0.02 | 0.16 | 0.00 | 0.01 | 0.07 |  |
| E101 Riboflavin | 0.08 | 0.02 | 0.57 | 0.01 | 0.05 |  |
| E120 Cochineal, Carminic acid, Carmines | 0.02 | 0.09 | 0.00 | 0.01 | 0.08 |  |
| E133 Brilliant blue FCF | 0.00 | 0.00 | 0.02 | 0.00 | 0.03 |  |
| Modified starches | 0.05 | 1.00 | 0.00 | 0.02 | 0.01 |  |
| E141 Copper complexes of chlorophyllis and chlorophyllins | 0.00 | 0.02 | 0.00 | 0.01 | 0.04 |  |
| E150a Plain caramel | 0.03 | 0.07 | 0.09 | 0.03 | 0.04 |  |
| E150c Ammonia caramel | 0.00 | 0.10 | 0.03 | 0.01 | 0.03 |  |
| E150d Sulphite ammonia caramel | 0.03 | -0.01 | 0.03 | 0.03 | 0.66 |  |
| E160 Carotene | 0.03 | 0.01 | 0.02 | 0.02 | 0.08 |  |
| E160a Carotenes | 0.02 | 0.04 | 0.00 | 0.01 | 0.10 |  |
| E160b Annatto, Bixin, Norbixin | 0.05 | 0.08 | 0.01 | 0.04 | 0.05 |  |
| E160c Paprika extract, capsanthin, capsorubin | 0.05 | 0.11 | 0.00 | 0.02 | 0.17 |  |
| E161b Lutein | 0.02 | 0.12 | 0.00 | 0.01 | 0.13 |  |
| E162 Beetroot Red, betanin | 0.02 | 0.05 | 0.00 | 0.01 | 0.06 |  |
| E163 Anthocyanins | 0.03 | 0.06 | 0.01 | 0.02 | 0.15 |  |
| E171 Titanium dioxide | 0.02 | 0.01 | 0.00 | 0.04 | 0.11 |  |
| E200 Sorbic acid | 0.06 | 0.02 | 0.00 | 0.05 | 0.01 |  |
| E202 Potassium sorbate | 0.07 | 0.18 | 0.01 | 0.03 | 0.09 |  |
| E203 Calcium sorbate | -0.01 | 0.01 | 0.00 | -0.01 | 0.00 |  |
| Food additive sulphites | 0.00 | -0.01 | 0.00 | -0.02 | -0.03 |  |
| E250 Sodium nitrite | 0.05 | 0.10 | 0.01 | 0.02 | 0.10 |  |
| E252 Potassium nitrate | 0.03 | -0.02 | 0.01 | 0.01 | 0.05 |  |
| E260 Acetic acid | 0.02 | 0.09 | 0.00 | 0.01 | 0.04 |  |
| E262 Sodium acetate | 0.02 | 0.05 | 0.00 | 0.01 | 0.02 |  |
| E270 Lactic acid | 0.01 | 0.04 | 0.00 | 0.00 | 0.06 |  |
| E282 Calcium propionate | 0.04 | 0.04 | 0.00 | 0.02 | 0.07 |  |
| E296 Malic acid | 0.08 | 0.01 | 0.01 | 0.03 | 0.18 |  |
| E300 Ascorbic acid | 0.11 | 0.06 | 0.00 | 0.03 | 0.06 |  |
| E301 Sodium ascorbate | 0.06 | 0.04 | 0.01 | 0.02 | 0.06 |  |
| E306 Tocopherols | 0.03 | 0.04 | 0.02 | 0.04 | 0.05 |  |
| E307 Alpha-tocopherol | 0.11 | 0.03 | 0.25 | 0.27 | 0.02 |  |
| E316 Sodium erythorbate | 0.05 | 0.10 | 0.01 | 0.01 | 0.06 |  |
| E322 Lecithins | 0.12 | 0.04 | 0.09 | 0.15 | 0.08 |  |
| E330 Citric acid | 0.13 | 0.09 | 0.09 | 0.04 | 0.78 |  |
| E331 Sodium citrates | 0.05 | 0.09 | 0.11 | 0.03 | 0.67 |  |
| E334 Tartaric acid | 0.01 | 0.05 | 0.00 | 0.01 | 0.01 |  |
| E338 Phosphoric acid | 0.03 | -0.01 | 0.03 | 0.03 | 0.66 |  |
| E339 Sodium phosphates | 0.02 | 0.08 | 0.01 | 0.01 | 0.02 |  |
| E340 Potassium phosphates | 0.02 | 0.01 | 0.01 | 0.00 | 0.03 |  |
| E341 Calcium phosphates | 0.07 | 0.07 | 0.05 | 0.08 | 0.02 |  |
| E392 Carnosic acid (Extract of rosemary) | 0.01 | 0.02 | 0.00 | 0.02 | 0.04 |  |
| E401 Sodium alginate | 0.08 | 0.04 | 0.01 | 0.02 | 0.04 |  |
| E407 Carrageenan | 0.07 | 0.26 | 0.01 | 0.04 | 0.07 |  |
| E407a Processed euchema seaweed | 0.01 | 0.04 | 0.00 | 0.01 | 0.05 |  |
| E410 Locust bean gum | 0.05 | 0.03 | 0.01 | 0.02 | 0.06 |  |
| E412 Guar gum | 0.03 | 0.27 | 0.01 | 0.01 | 0.14 |  |
| E414 Arabic gum | 0.03 | 0.00 | 0.01 | 0.03 | 0.24 |  |
| E415 Xanthan gum | 0.05 | 0.16 | 0.01 | 0.03 | 0.11 |  |
| E420 Sorbitols | 0.17 | 0.02 | 0.01 | 0.08 | 0.09 |  |
| E422 Glycerol | 0.37 | 0.05 | 0.01 | 0.11 | 0.07 |  |
| E440 Pectins | 0.12 | 0.33 | 0.00 | 0.02 | 0.13 |  |
| E442 Ammonium phosphatides | 0.02 | -0.01 | 0.00 | 0.01 | 0.03 |  |
| E450 Diphosphates | 0.79 | 0.07 | 0.08 | 0.31 | 0.07 |  |
| E451 Triphosphates | 0.03 | 0.12 | 0.00 | 0.02 | 0.06 |  |
| E452 Polyphosphates | 0.02 | 0.20 | 0.01 | 0.01 | 0.06 |  |
| E460 Cellulose (microcrystalline cellulose) | 0.05 | 0.06 | 0.01 | 0.08 | 0.04 |  |
| E466 Carboxy methyl cellulose | 0.02 | 0.04 | 0.03 | 0.02 | 0.04 |  |
| E471 Mono- and diglycerides of fatty acids | 0.10 | 0.14 | 0.00 | 0.08 | 0.12 |  |
| E472b Lactic acid esters of mono- and diglycerides of fatty acids | 0.04 | 0.06 | 0.01 | 0.03 | 0.03 |  |
| E472c Citric acid esters of mono- and diglycerides of fatty acids | 0.01 | 0.10 | 0.01 | 0.00 | 0.02 |  |
| E472e Mono and diacetyl tartaric acid esters of mono and diglycerides of fatty acids | 0.08 | 0.01 | 0.11 | 0.18 | 0.05 |  |
| E476 Polyglycerol polyricinoleate | 0.04 | 0.07 | 0.00 | 0.10 | 0.02 |  |
| E481 Sodium stearoyl-2-lactylate | 0.02 | 0.01 | 0.00 | 0.02 | 0.04 |  |
| E500 Sodium carbonates | 1.00 | 0.03 | 0.15 | 0.36 | 0.04 |  |
| E501 Potassium carbonates | 0.17 | 0.01 | 0.00 | 0.00 | 0.00 |  |
| E503 Ammonium carbonates | 0.36 | 0.01 | 0.17 | 1.00 | 0.02 |  |
| E504 Magnesium carbonates | 0.15 | -0.01 | 1.00 | 0.17 | 0.04 |  |
| E579 Ferrous gluconate | 0.02 | 0.02 | 0.00 | 0.01 | 0.01 |  |
| E621 Monosodium glutamate | 0.01 | 0.12 | 0.00 | -0.01 | 0.04 |  |
| E901 Beeswax | 0.02 | 0.00 | 0.01 | 0.02 | 0.09 |  |
| E903 Carnauba wax | 0.04 | 0.00 | 0.01 | 0.03 | 0.18 |  |
| E950 Acesulfame K | 0.02 | 0.08 | 0.04 | 0.02 | 0.60 |  |
| E951 Aspartame | 0.00 | 0.07 | 0.02 | 0.00 | 0.45 |  |
| E955 Sucralose | 0.01 | 0.02 | 0.02 | 0.00 | 0.24 |  |

#### NMF performed on mean daily food additive intakes in the 2017-2024 period

|  | **Mixtures** | | | | |
| --- | --- | --- | --- | --- | --- |
| **Food additives** | **1** | **2** | **3** | **4** | **5** |
| E100 Curcumin | 0.02 | 0.14 | 0.02 | 0.07 | 0.00 |
| E101 Riboflavin | 0.02 | 0.03 | 0.00 | 0.04 | 0.33 |
| E120 Cochineal, Carminic acid, Carmines | 0.03 | 0.09 | 0.02 | 0.09 | 0.00 |
| E133 Brilliant blue FCF | 0.01 | 0.01 | 0.01 | 0.02 | 0.03 |
| Modified starches | 0.06 | 1.00 | 0.04 | 0.14 | 0.01 |
| E141 Copper complexes of chlorophyllis and chlorophyllins | 0.01 | 0.05 | 0.02 | 0.05 | 0.01 |
| E150a Plain caramel | 0.02 | 0.07 | 0.03 | 0.05 | 0.01 |
| E150c Ammonia caramel | 0.02 | 0.06 | 0.02 | 0.02 | 0.00 |
| E150d Sulphite ammonia caramel | 0.03 | 0.02 | 0.01 | 0.25 | 0.08 |
| E160 Carotene | 0.03 | 0.03 | 0.02 | 0.06 | 0.01 |
| E160a Carotenes | 0.01 | 0.05 | 0.02 | 0.10 | 0.01 |
| E160b Annatto, Bixin, Norbixin | 0.04 | 0.11 | 0.03 | 0.07 | 0.00 |
| E160c Paprika extract, capsanthin, capsorubin | 0.06 | 0.10 | 0.02 | 0.13 | 0.00 |
| E161b Lutein | 0.02 | 0.10 | 0.00 | 0.10 | 0.01 |
| E162 Beetroot Red, betanin | 0.01 | 0.04 | 0.01 | 0.08 | 0.00 |
| E163 Anthocyanins | 0.02 | 0.06 | 0.01 | 0.14 | 0.08 |
| E171 Titanium dioxide | 0.01 | 0.02 | 0.02 | 0.06 | 0.01 |
| E200 Sorbic acid | 0.08 | 0.01 | 0.01 | 0.02 | 0.02 |
| E202 Potassium sorbate | 0.08 | 0.23 | 0.03 | 0.18 | 0.01 |
| E203 Calcium sorbate | -0.02 | 0.02 | -0.01 | 0.00 | 0.00 |
| Food additive sulphites | 0.02 | 0.03 | 0.00 | 0.03 | 0.00 |
| E250 Sodium nitrite | 0.08 | 0.16 | 0.03 | 0.13 | 0.00 |
| E252 Potassium nitrate | 0.07 | 0.03 | 0.01 | 0.06 | 0.01 |
| E260 Acetic acid | 0.02 | 0.10 | 0.00 | 0.04 | 0.00 |
| E262 Sodium acetate | 0.04 | 0.11 | 0.02 | 0.06 | 0.00 |
| E270 Lactic acid | 0.04 | 0.06 | 0.00 | 0.09 | 0.00 |
| E282 Calcium propionate | 0.07 | 0.07 | 0.03 | 0.11 | 0.01 |
| E296 Malic acid | 0.14 | 0.02 | 0.05 | 0.20 | 0.02 |
| E300 Ascorbic acid | 0.09 | 0.10 | 0.06 | 0.13 | 0.00 |
| E301 Sodium ascorbate | 0.04 | 0.09 | 0.02 | 0.07 | 0.00 |
| E306 Tocopherols | 0.03 | 0.02 | 0.02 | 0.07 | 0.04 |
| E307 Alpha-tocopherol | 0.01 | 0.06 | 0.03 | 0.03 | 0.09 |
| E316 Sodium erythorbate | 0.11 | 0.18 | 0.02 | 0.11 | 0.00 |
| E322 Lecithins | 0.12 | 0.05 | 0.13 | 0.10 | 0.06 |
| E330 Citric acid | 0.14 | 0.15 | 0.04 | 0.95 | 0.07 |
| E331 Sodium citrates | 0.06 | 0.12 | 0.03 | 0.38 | 0.17 |
| E334 Tartaric acid | 0.03 | 0.08 | 0.00 | 0.02 | 0.00 |
| E338 Phosphoric acid | 0.03 | 0.02 | 0.01 | 0.26 | 0.08 |
| E339 Sodium phosphates | 0.01 | 0.07 | 0.01 | 0.05 | 0.02 |
| E340 Potassium phosphates | 0.02 | 0.01 | 0.00 | 0.04 | 0.00 |
| E341 Calcium phosphates | 0.07 | 0.05 | 0.06 | 0.10 | 0.00 |
| E392 Carnosic acid (Extracts of rosemary) | 0.02 | 0.05 | 0.03 | 0.07 | 0.01 |
| E401 Sodium alginate | 0.07 | 0.04 | 0.02 | 0.09 | 0.00 |
| E407 Carrageenan | 0.08 | 0.28 | 0.05 | 0.10 | 0.00 |
| E407a Processed euchema seaweed | 0.02 | 0.06 | 0.01 | 0.05 | 0.00 |
| E410 Locust bean gum | 0.07 | 0.06 | 0.02 | 0.10 | 0.01 |
| E412 Guar gum | 0.03 | 0.22 | 0.01 | 0.19 | 0.00 |
| E414 Arabic gum | 0.03 | 0.00 | 0.05 | 0.15 | 0.00 |
| E415 Xanthan gum | 0.05 | 0.20 | 0.01 | 0.29 | 0.00 |
| E420 Sorbitols | 0.14 | 0.04 | 0.06 | 0.09 | 0.02 |
| E422 Glycerol | 0.37 | 0.05 | 0.13 | 0.24 | 0.01 |
| E440 Pectins | 0.14 | 0.24 | 0.03 | 0.18 | 0.00 |
| E442 Ammonium phosphatides | 0.02 | 0.00 | 0.01 | 0.02 | 0.00 |
| E450 Diphosphates | 0.70 | 0.13 | 0.25 | 0.24 | 0.01 |
| E451 Triphosphates | 0.02 | 0.15 | 0.02 | 0.11 | 0.01 |
| E452 Polyphosphates | 0.01 | 0.24 | 0.01 | 0.17 | 0.00 |
| E460 Cellulose (microcrystalline cellulose) | 0.04 | 0.07 | 0.04 | 0.03 | 0.01 |
| E466 Carboxy methyl cellulose | 0.05 | 0.02 | 0.02 | 0.05 | 0.01 |
| E471 Mono- and diglycerides of fatty acids | 0.13 | 0.20 | 0.10 | 0.21 | 0.00 |
| E472b Lactic acid esters of mono- and diglycerides of fatty acids | 0.02 | 0.05 | 0.03 | 0.02 | 0.00 |
| E472c Citric acid esters of mono- and diglycerides of fatty acids | 0.00 | 0.09 | 0.01 | 0.04 | 0.02 |
| E472e Mono and diacetyl tartaric acid esters of mono and diglycerides of fatty acids | 0.04 | 0.02 | 0.07 | 0.07 | 0.00 |
| E476 Polyglycerol polyricinoleate | 0.02 | 0.09 | 0.05 | 0.05 | 0.01 |
| E481 Sodium stearoyl-2-lactylate | 0.02 | 0.04 | 0.03 | 0.06 | 0.00 |
| E500 Sodium carbonates | 1.00 | 0.05 | 0.28 | 0.15 | 0.02 |
| E501 Potassium carbonates | 0.13 | -0.01 | 0.01 | -0.01 | 0.00 |
| E503 Ammonium carbonates | 0.27 | 0.04 | 1.00 | 0.03 | 0.01 |
| E504 Magnesium carbonates | 0.02 | 0.00 | 0.01 | 0.01 | 1.00 |
| E579 Ferrous gluconate | 0.02 | 0.04 | 0.00 | 0.03 | 0.00 |
| E621 Monosodium glutamate | 0.00 | 0.14 | 0.00 | 0.18 | 0.01 |
| E901 Beeswax | 0.02 | 0.00 | 0.01 | 0.09 | 0.02 |
| E903 Carnauba wax | 0.02 | 0.00 | 0.02 | 0.14 | 0.02 |
| E950 Acesulfame K | 0.02 | 0.07 | 0.00 | 0.35 | 0.06 |
| E951 Aspartame | 0.02 | 0.05 | 0.01 | 0.25 | 0.05 |
| E955 Sucralose | 0.00 | 0.01 | 0.00 | 0.38 | 0.01 |

Only a few additives were not consistent (e.g. E307 and E472e found in period 1 and not in period 2 and conversely for E621), which can be attributed to market evolutions and reformulations, but most of the additives and their grouping in the five mixtures were identical.

## Table D. Spearman correlations between the five NMF food additive mixtures

|  | **Mixture 1** | **Mixture 2** | **Mixture 3** | **Mixture 4** | **Mixture 5** |
| --- | --- | --- | --- | --- | --- |
| **Mixture 1** | 1.00 |  |  |  |  |
| **Mixture 2** | 0.02 | 1.00 |  |  |  |
| **Mixture 3** | 0.02 | 0.04 | 1.00 |  |  |
| **Mixture 4** | 0.39 | -0.04 | 0.05 | 1.00 |  |
| **Mixture 5** | 0.07 | 0.04 | -0.009 | 0.04 | 1.00 |

## Table E. Spearman correlations between NMF food additive mixtures and food group intakes.

|  | **Mixtures** | | | | |
| --- | --- | --- | --- | --- | --- |
| **Food groups** | **1** | **2** | **3** | **4** | **5** |
| Fruits and vegetables | -0.03 | 0.06 | 0.04 | -0.06 | -0.09 |
| Broth^a^ | -0.01 | 0.40 | 0.05 | -0.04 | -0.14 |
| Potatoes and tubers | 0.02 | 0.14 | -0.01 | 0.02 | -0.02 |
| Refined grains and cereals | 0.03 | 0.04 | -0.03 | 0.03 | 0.03 |
| Whole grains and cereals | -0.02 | -0.01 | 0.02 | -0.02 | -0.09 |
| Pulses and legumes | 0.03 | 0.02 | 0.02 | 0.03 | -0.06 |
| Nuts, seeds and dried fruits | 0.12 | -0.01 | 0.00 | 0.09 | 0.02 |
| Dairy products | 0.06 | 0.09 | 0.01 | 0.05 | 0.06 |
| Dairy desserts | 0.09 | 0.22 | 0.03 | 0.09 | 0.07 |
| Red meat and offals | 0.00 | 0.06 | -0.02 | -0.01 | 0.07 |
| Poultry | 0.02 | 0.07 | -0.01 | 0.00 | 0.07 |
| Processed meat | 0.02 | 0.07 | -0.01 | 0.00 | 0.07 |
| Eggs | 0.05 | 0.03 | 0.01 | 0.02 | 0.02 |
| Fish and seafood | -0.01 | 0.04 | -0.01 | 0.00 | -0.03 |
| Processed fish and seafood | 0.06 | 0.05 | 0.00 | 0.05 | 0.06 |
| Fats and sauces | 0.08 | 0.21 | -0.05 | 0.05 | 0.10 |
| Other seasonings | 0.02 | -0.04 | 0.00 | 0.01 | 0.02 |
| Breakfast cereals | 0.07 | 0.00 | 0.02 | 0.08 | 0.06 |
| Confectionery | 0.09 | 0.05 | -0.01 | 0.07 | 0.04 |
| Cakes and biscuits | 0.35 | 0.09 | -0.02 | 0.18 | 0.06 |
| Pastries | 0.13 | 0.03 | -0.03 | 0.14 | 0.11 |
| Artificially sweetened drinks | 0.03 | 0.04 | -0.14 | 0.01 | 0.41 |
| Tea, coffee, water | 0.05 | 0.02 | 0.04 | 0.04 | -0.04 |
| Sugary drinks | 0.13 | 0.00 | -0.01 | 0.09 | 0.37 |
| Alcoholic drinks | 0.05 | -0.01 | 0.00 | 0.02 | -0.03 |
| Meal replacements | 0.01 | 0.04 | 0.04 | 0.01 | 0.04 |
| Savoury snacks | 0.18 | 0.01 | 0.00 | 0.19 | 0.10 |

^a^“Broth” refers to a food item called "bouillon" in French, which is a clarified soup or seasoned liquid typically made by simmering meat, vegetables, and seasonings in water. It can be consumed as a standalone dish.

## Table F. Food group consumption of participants according to sex-specific quintiles of mixtures 2 and 5^a^.

### Mixture 2

|  | **Mixture 2** | | | | |
| --- | --- | --- | --- | --- | --- |
| Intakes (g/d for solid foods and ml/d for liquids) | Q1 | Q2 | Q3 | Q4 | Q5 |
|  | (N=21730) | (N=21728) | (N=21728) | (N=21728) | (N=21729) |
| Fruits and vegetables |  |  |  |  |  |
| Mean (SD) | 480.9 (248.2) | 477.5 (223.5) | 475.6 (214.8) | 464.6 (218.9) | 423.7 (240.9) |
| Median [25th – 75th percentiles] | 439 [291.7, 608.8] | 427.6 [295.6 - 575.7] | 423.4 [299.5, 565.7] | 433.7 [308.4, 573.7] | 476.5 [342.4, 626.9] |
| Broth |  |  |  |  |  |
| Mean (SD) | 13.7 (35.3) | 14.6 (30.6) | 19.1 (32.8) | 28.1 (36.7) | 60.0 (56.6) |
| Median [25th – 75th percentiles] | 0.0 [0.0 - 6.6] | 0.0 [0.0 - 16.6] | 2.6 [0.0, 27.4] | 15.9 [0.0, 43.9] | 52.5 [4.3, 92.5] |
| Potatoes and tubers |  |  |  |  |  |
| Mean (SD) | 43.3 (51.0) | 44.3 (43.2) | 45.5 (42.3) | 47.9 (42.7) | 56.5 (48.9) |
| Median [25th – 75th percentiles] | 28.6 [0.0 - 64.3] | 34.8 [12.1 - 64.3] | 36.0 [14.3, 65.5] | 39.3 [15.8, 68.6] | 46.1 [22.2, 78.7] |
| Refined grains and cereals |  |  |  |  |  |
| Mean (SD) | 141.5 (88.8) | 143.0 (79.9) | 144.7 (78.9) | 146.9 (78.6) | 145.4 (86.8) |
| Median [25th – 75th percentiles] | 126.8 [75.9 - 186.7] | 133.4 [87.6 - 187.4] | 136.4 [92.1, 188.1] | 136.1 [91.7, 188.7] | 134.1 [83.9, 192.1] |
| Whole grains and cereals |  |  |  |  |  |
| Mean (SD) | 39.9 (55.0) | 34.3 (44.8) | 32.2 (42.6) | 31.8 (40.8) | 35.9 (47.3) |
| Median [25th – 75th percentiles] | 21.4 [0.0 - 58.8] | 19.1 [0.0 - 50.4] | 17.9 [0.0, 47.1] | 17.9 [0.0, 46.6] | 20.0 [0.0, 53.6] |
| Pulses and legumes |  |  |  |  |  |
| Mean (SD) | 15.7 (33.3) | 12.3 (24.1) | 11.6 (21.8) | 11.6 (21.6) | 12.1 (23.1) |
| Median [25th – 75th percentiles] | 0.0 [0.0 - 17.9] | 0.0 [0.0 - 16.1] | 0.0 [0.0, 15.2] | 0.0 [0.0, 15.4] | 0.0 [0.0, 15.2] |
| Nuts seeds and dried fruits |  |  |  |  |  |
| Mean (SD) | 9.4 (19.9) | 6.9 (13.0) | 6.6 (12.8) | 6.2 (12.2) | 6.9 (15.1) |
| Median [25th – 75th percentiles] | 0.7 [0.0 - 10.7] | 1.5 [0.0 - 8.5] | 1.5 [0.0, 7.7] | 1.4 [0.0, 7.3] | 0.7 [0.0, 7.7] |
| Dairy products |  |  |  |  |  |
| Mean (SD) | 187.3 (154.3) | 189.1 (143.7) | 194.1 (140.9) | 201.2 (145.1) | 287.9 (154.4) |
| Median [25th – 75th percentiles] | 137.4 [61.1 - 252.1] | 150.7 [80.6 - 257.1] | 160.7 [88.7, 266.6] | 169.9 [96.6, 277.3] | 191.9 [107.3, 307.3] |
| Dairy desserts |  |  |  |  |  |
| Mean (SD) | 20.8 (39.2) | 30.4 (40.2) | 36.8 (43.6) | 43.4 (49.5) | 49.0 (61.1) |
| Median [25th – 75th percentiles] | 0.0 [0.0 - 29.8] | 17.9 [0.0 - 44.6] | 24.3 [0.0, 56.6] | 30.0 [0.0, 67.0] | 31.5 [0.0, 75.9] |
| Redmeat and offals |  |  |  |  |  |
| Mean (SD) | 42.3 (46.0) | 44.1 (39.1) | 45.3 (38.5) | 45.3 (37.9) | 45.8 (40.5) |
| Median [25th – 75th percentiles] | 31.0 [0.0 - 65.0] | 37.5 [14.3- 64.3] | 38.5 [17.1, 65.0] | 39.1 [17.2, 64.7] | 38.6 [15.2, 67.0] |
| Poultry |  |  |  |  |  |
| Mean (SD) | 23.7 (33.6) | 24.0 (28.0) | 24.8 (27.6) | 25.5 (28.0) | 26.0 (30.5) |
| Median [25th – 75th percentiles] | 11.3 [0.0 - 37.1] | 16.5 [0.0 - 36.3] | 18.4 [2.3, 36.5] | 18.9 [2.6, 37.5] | 18.3 [0.0, 38.3] |
| Processed meat |  |  |  |  |  |
| Mean (SD) | 52.6 (47.6) | 55.1 (40.9) | 56.9 (39.8) | 57.7 (40.3) | 57.1 (42.6) |
| Median [25th – 75th percentiles] | 43.5 [17.7 - 75.2] | 48.4 [26.9 - 75.1] | 50.5 [29.8, 76.7] | 51.6 [30.0, 78.3] | 50.0 [27.1, 78.1] |
| Eggs |  |  |  |  |  |
| Mean (SD) | 14.7 (23.3) | 13.8 (18.4) | 13.8 (17.6) | 13.8 (18.0) | 14.0 (20.2) |
| Median [25th – 75th percentiles] | 4.8 [0.0 - 21.0] | 7.1 [0.8 - 19.6] | 7.5 [1.2, 19.7] | 7.4 [1.2, 19.3] | 7.1 [0.0, 19.6] |
| Fish and seafood |  |  |  |  |  |
| Mean (SD) | 36.8 (43.1) | 36.9 (36.8) | 37.2 (36.0) | 37.2 (36.5) | 37.8 (38.7) |
| Median [25th – 75th percentiles] | 25.2 [0.0 - 55.4] | 28.6 [8.1 - 54.5] | 29.3 [9.7, 54.3] | 29.4 [9.5, 53.8] | 29.5 [6.7, 55.9] |
| Processed fish and seafood |  |  |  |  |  |
| Mean (SD) | 2.4 (10.3) | 2.8 (9.2) | 2.9 (9.5) | 3.0 (9.4) | 3.1 (10.7) |
| Median [25th – 75th percentiles] | 0.0 [0.0 - 0.0] | 0.0 [0.0 - 0.0] | 0.0 [0.0 - 0.0] | 0.0 [0.0, 0.0] | 0.0 [0.0, 0.0] |
| Fats and sauce |  |  |  |  |  |
| Mean (SD) | 35.5 (20.6) | 40.1 (19.9) | 42.8 (20.5) | 45.9 (21.6) | 50.8 (25.7) |
| Median [25th – 75th percentiles] | 32.5 [21.4 - 45.8] | 37.6 [26.4 - 50.4] | 40.1 [29.2, 53.3] | 43.2 [31.0, 57.2] | 46.9 [33.9, 62.9] |
| Breakfast cereals |  |  |  |  |  |
| Mean (SD) | 7.9 (18.2) | 7.3 (16.1) | 7.0 (15.0) | 7.0 (15.5) | 6.8 (15.7) |
| Median [25th – 75th percentiles] | 0.0 [0.0 - 6.0] | 0.0 [0.0 - 7.1] | 0.0 [0.0, 7.3] | 0.0 [0.0, 7.1] | 0.0 [0.0, 5.1] |
| Confectionery |  |  |  |  |  |
| Mean (SD) | 40.0 (37.5) | 42.8 (34.6) | 43.6 (33.9) | 43.9 (34.5) | 41.6 (36.1) |
| Median [25th – 75th percentiles] | 31.4 [14.1 - 55.4] | 35.9 [18.6 - 58.6] | 36.9 [19.7, 59.4] | 37.1 [19.4, 59.6] | 34.3 [16.4, 57.1] |
| Cakes and biscuits |  |  |  |  |  |
| Mean (SD) | 32.2 (39.9) | 39.3 (38.9) | 42.6 (39.8) | 45.9 (42.6) | 45.7 (47.7) |
| Median [25th – 75th percentiles] | 20.7 [0.0 - 46.7] | 30.2 [10.7 - 56.8] | 33.9 [13.3, 61.5] | 36.5 [14.3, 65.7] | 33.9 [10.6, 65.4] |
| Pastries |  |  |  |  |  |
| Mean (SD) | 8.9 (19.4) | 10.0 (18.3) | 10.6 (18.6) | 10.6 (18.8) | 9.5 (18.8) |
| Median [25th – 75th percentiles] | 0.0 [0.0 - 10.0] | 0.0 [0.0 - 14.3] | 0.0 [0.0, 14.3] | 0.0 [0.0, 14.8] | 0.0 [0.0, 12.5] |
| Artificially sweetened drinks |  |  |  |  |  |
| Mean (SD) | 4.9 (37.9) | 5.2 (30.9) | 6.7 (30.1) | 14.3 (38.5) | 98.0 (181.1) |
| Median [25th – 75th percentiles] | 0.0 [0.0 - 0.0] | 0.0 [0.0 - 0.0] | 0.0 [0.0, 0.0] | 0.0 [0.0, 0.0] | 0.0 [0.0, 130.9] |
| Tea coffee water |  |  |  |  |  |
| Mean (SD) | 1066.9 (542.2) | 1098.1 (529.9) | 1102.2 (515.9) | 1096.7 (521.9) | 1089.8 (586.7) |
| Median [25th – 75th percentiles] | 992.9 [682.8 - 1392.9] | 1012.9 [709.5 - 1372.9] | 1035.7 [719.3, 1369.3] | 1021.2 [728.6, 1368.8] | 1036.9 [738.6, 1399.3] |
| Sugary drinks |  |  |  |  |  |
| Mean (SD) | 48.8 (116.4) | 50.5 (108.0) | 49.6 (101.8) | 47.9 (101.2) | 40.0 (98.2) |
| Median [25th – 75th percentiles] | 0.0 [0.0 - 47.6] | 0.0 [0.0 - 57.1] | 0.0 [0.0, 58.3] | 0.0 [0.0, 56.6] | 0.0 [0.0, 0.0] |
| Alcoholic drinks |  |  |  |  |  |
| Mean (SD) | 96.7 (152.5) | 99.6 (145.8) | 100.1 (145.9) | 96.2 (148.4) | 85.3 (144.5) |
| Median [25th – 75th percentiles] | 32.1 [0.0 - 128.6] | 46.4 [0.0 - 141.7] | 46.4 [0.0, 138.9] | 42.9 [0.0, 128.6] | 29.8 [0.0, 108.9] |
| Meal replacements |  |  |  |  |  |
| Mean (SD) | 1.3 (19.2) | 0.9 (11.5) | 1.1 (12.5) | 1.5 (13.9) | 3. 8 (29.6) |
| Median [25th – 75th percentiles] | 0.0 [0.0 - 0.0] | 0.0 [0.0 - 0.0] | 0.0 [0.0 - 0.0] | 0.0 [0.0, 0.0] | 0.0 [0.0, 0.0] |
| Savory snacks |  |  |  |  |  |
| Mean (SD) | 9.5 (15.8) | 11.2 (14.5) | 12.1 (14.6) | 12.1 (15.1) | 11.1 (15.9) |
| Median [25th – 75th percentiles] | 2.9 [0.0 - 13.3] | 7.1 [0.0 - 16.4] | 8.1 [0.0, 17.9] | 8.0 [0.0, 17.9] | - 1. [0.0, 16.2] |

### Mixture 5

|  | **Mixture 5** | | | | |
| --- | --- | --- | --- | --- | --- |
|  | Q1 | Q2 | Q3 | Q4 | Q5 |
|  | (N=21730) | (N=21728) | (N=21728) | (N=21728) | (N=21729) |
| Fruits and vegetables |  |  |  |  |  |
| Mean (SD) | 480.9 (248.2) | 477.5 (223.5) | 475.6 (214.8) | 464.6 (218.9) | 423.7 (240.9) |
| Median [25th – 75th percentiles] | 451.2 [313.6 - 609.5] | 456.4 [327.1 - 598.5] | 454.7 [328.3 - 595.8] | 442.5 [313.7 - 585.5] | 391.2 [255.2 - 553.3] |
| Broth |  |  |  |  |  |
| Mean (SD) | 39.2 (52.2) | 25.9 (40.0) | 25.0 (38.4) | 24.3 (39.1) | 21.2 (42.0) |
| Median [25th – 75th percentiles] | 16.3 [0.0 - 65.6] | 5.4 [0.0 - 39.6] | 5.7 [0.0 - 37.5] | 3.0 [0.0 - 35.7] | 0.0 [0.0 - 26.2] |
| Potatoes and tubers |  |  |  |  |  |
| Mean (SD) | 49.6 (50.1) | 46.8 (44.5) | 47.1 (43.0) | 47.6 (44.9) | 46.4 (47.1) |
| Median [25th – 75th percentiles] | 37.5 [12.1 - 71.4] | 36.7 [14.3 - 67.1] | 38.4 [15.7 - 66.7] | 37.5 [14.3 - 68.3] | 35.7 [10.7 - 67.9] |
| Refined grains and cereals |  |  |  |  |  |
| Mean (SD) | 141.5 (88.9) | 143.0 (81.6) | 144.7 (79.2) | 146.9 (80.8) | 145.4 (82.8) |
| Median [25th – 75th percentiles] | 128.7 [77.9 - 189.3] | 132.4 [85.7 - 187.1] | 134.3 [89.3 - 187.7] | 136.4 [90.7 - 190.1] | 135.0 [87.9 - 189.5] |
| Whole grains and cereals |  |  |  |  |  |
| Mean (SD) | 40.1 (54.0) | 38.5 (47.6) | 35.3 (44.8) | 32.3 (43.0) | 27.9 (40.8) |
| Median [25th – 75th percentiles] | 21.4 [0.0 - 58.9] | 23.0 [0.0 - 57.1] | 20.7 [0.0 - 51.4] | 17.9 [0.0 - 47.1] | 12.5 [0.0 - 40.0] |
| Pulses and legumes |  |  |  |  |  |
| Mean (SD) | 15.0 (29.8) | 14.0 (25.8) | 12.9 (24.5) | 11.6 (22.8) | 9.8 (22.1) |
| Median [25th – 75th percentiles] | 0.0 [0.0 - 18.6] | 0.0 [0.0 - 18.6] | 0.0 [0.0 - 17.4] | 0.0 [0.0 - 14.3] | 0.0 [0.0 - 10.0] |
| Nuts seeds and dried fruits |  |  |  |  |  |
| Mean (SD) | 9.0 (19.1) | 8.3 (15.4) | 7.2 (13.8) | 6.3 (12.7) | 5.3 (12.1) |
| Median [25th – 75th percentiles] | 0.8 [0.0 - 10.1] | 2.1 [0.0 - 10.1] | 2.0 [0.0 - 8.7] | 1.3 [0.0 - 7.2] | 0.0 [0.0 - 5.7] |
| Dairy products |  |  |  |  |  |
| Mean (SD) | 187.3 (154.3) | 189.1 (143.7) | 194.1 (140.9) | 202.2 (145.1) | 204.5 (154.4) |
| Median [25th – 75th percentiles] | 150.8 [73.2 - 264.4] | 156.4 [83.0 - 264.3] | 161.4 [90.8 - 267.9] | 170.3 [95.4 - 280.0] | 171.2 [87.5 - 287.9] |
| Dairy desserts |  |  |  |  |  |
| Mean (SD) | 32.5 (49.2) | 32.7 (44.2) | 35.4 (44.8) | 38.4 (47.8) | 41.4 (54.8) |
| Median [25th – 75th percentiles] | 8.3 [0.0 - 44.6] | 16.7 [0.0 - 48.1] | 22.3 [0.0 - 53.6] | 23.8 [0.0 - 58.0] | 23.8 [0.0 - 64.3] |
| Red meat and offals |  |  |  |  |  |
| Mean (SD) | 42.1 (42.4) | 42.8 (38.6) | 44.0 (38.0) | 45.6 (38.5) | 48.4 (44.3) |
| Median [25th – 75th percentiles] | 34.3 [5.6 - 63.0] | 36.4 [12.9 - 62.8] | 37.5 [15.6 - 64.0] | 39.2 [16.8 - 66.0] | 40.1 [15.5 - 69.9] |
| Poultry |  |  |  |  |  |
| Mean (SD) | 23.1 (30.8) | 23.6 (27.9) | 24.2 (27.0) | 25.5 (28.6) | 27.7 (33.3) |
| Median [25th – 75th percentiles] | 12.5 [0.0 - 35.7] | 16.1 [0.0 - 35.6] | 17.9 [1.3 - 36.3] | 18.6 [1.5 - 37.5] | 19.0 [0.0 - 40.3] |
| Processed meat |  |  |  |  |  |
| Mean (SD) | 49.7 (42.7) | 52.3 (39.8) | 55.0 (38.8) | 58.4 (41.0) | 63.9 (47.7) |
| Median [25th – 75th percentiles] | 41.8 [17.9 - 71.0] | 46.2 [24.9 - 72.1] | 49.4 [28.4 - 74.6] | 52.5 [30.3 - 79.4] | 55.9 [31.4 - 86.5] |
| Eggs |  |  |  |  |  |
| Mean (SD) | 13.8 (21.6) | 13.8 (17.9) | 13.9 (17.6) | 14.4 (19.3) | 14.2 (21.4) |
| Median [25th – 75th percentiles] | 5.1 [0.0 - 18.9] | 7.4 [0.7 - 20.0] | 8.0 [1.4 - 19.9] | 7.5 [1.0 - 20.5] | 6.0 [0.0 - 19.1] |
| Fish and seafood |  |  |  |  |  |
| Mean (SD) | 37.4 (41.8) | 38.4 (37.4) | 38.2 (36.6) | 37.8 (37.2) | 34.2 (38.3) |
| Median [25th – 75th percentiles] | 27.5 [1.4 - 56.1] | 30.5 [9.5 - 56.0] | 30.6 [10.7 - 55.7] | 29.6 [8.9 - 55.0] | 24.5 [2.9 - 50.9] |
| Processed fish and seafood |  |  |  |  |  |
| Mean (SD) | 2.3 (8.8) | 2.5 (8.4) | 2.7 (8.8) | 3.1 (9.5) | 3.85 (12.9) |
| Median [25th – 75th percentiles] | 0.0 [0.0 - 0.0] | 0.0 [0.0 - 0.0] | 0.0 [0.0 - 0.0] | 0.0 [0.0 - 0.0] | 0.0 [0.0 - 0.0] |
| Fats and sauce |  |  |  |  |  |
| Mean (SD) | 42.0 (23.3) | 42.0 (21.0) | 43.2 (20.8) | 43.9 (21.9) | 43.9 (24.6) |
| Median [25th – 75th percentiles] | 38.8 [26.0 - 53.6] | 39.3 [27.6 - 52.7] | 40.5 [29.2 - 54.1] | 41.1 [29.2 - 55.0] | 40.2 [27.5 - 55.6] |
| Breakfast cereals |  |  |  |  |  |
| Mean (SD) | 7.1 (17.3) | 6.9 (16.1) | 6.9 (15.5) | 6.9 (14.9) | 8.1 (16.8) |
| Median [25th – 75th percentiles] | 0.0 [0.0 - 3.8] | 0.0 [0.0 - 5.6] | 0.0 [0.0 - 0.0] | 0.0 [0.0 - 7.1] | 0.0 [0.0 - 8.9] |
| Confectionery |  |  |  |  |  |
| Mean (SD) | 35.2 (32.1) | 40.1 (32.5) | 42.2 (31.8) | 45.1 (35.0) | 49.2 (42.8) |
| Median [25th – 75th percentiles] | 28.6 [12.4 - 48.9] | 33.9 [17.9 - 54.8] | 0.0 [0.0 - 0.0] | 38.5 [20.4 - 61.8] | 40.0 [19.2 - 68.2] |
| Cakes and biscuits |  |  |  |  |  |
| Mean (SD) | 35.5 (42.6) | 36.7 (36.9) | 40.9 (38.8) | 44.8 (42.2) | 47.8 (48.3) |
| Median [25th – 75th percentiles] | 23.2 [0.0 - 52.1] | 28.3 [9.2 - 53.2] | 0.0 [0.0 - 0.0] | 35.5 [13.6 - 64.5] | 35.7 [11.4 - 69.6] |
| Pastries |  |  |  |  |  |
| Mean (SD) | 7.3 (16.1) | 8.8 (17.5) | 9.6 (17.5) | 10.9 (19.0) | 13.0 (22.6) |
| Median [25th – 75th percentiles] | 0.0 [0.0 - 7.5] | 0.0 [0.0 - 11.4] | 0.0 [0.0 - 0.0] | 0.0 [0.0 - 15.2] | 0.0 [0.0 - 18.9] |
| Artificially sweetened drinks |  |  |  |  |  |
| Mean (SD) | 4.9 (37.9) | 5.2 (30.9) | 6.7 (30.1) | 14.3 (38.5) | 98.0 (187.6) |
| Median [25th – 75th percentiles] | 0.0 [0.0 - 0.0] | 0.0 [0.0 - 0.0] | 0.0 [0.0 - 0.0] | 0.0 [0.0 - 0.0] | 0.0 [0.0 - 131.0] |
| Tea coffee water |  |  |  |  |  |
| Mean (SD) | 1066.9 (542.3) | 1098.1 (529.9) | 1102.2 (515.9) | 1096.7 (521.9) | 1089.8 (586.7) |
| Median [25th – 75th percentiles] | 992.9 [690.0 - 1353.6] | 1025.9 [731.6 - 1381.6] | 0.0 [0.0 - 0.0] | 1026.9 [733.9 - 1383.8] | 1009.5 [684.3 - 1403.6] |
| Sugary drinks |  |  |  |  |  |
| Mean (SD) | 17.4 (55.0) | 19.9 (51.2) | 27.1 (55.1) | 45.5 (71.8) | 126.8 (182.5) |
| Median [25th – 75th percentiles] | 0.0 [0.0 - 0.0] | 0.0 [0.0 - 0.0] | 0.0 [0.0 - 34.3] | 14.3 [0.0 - 69.7] | 64.3 [0.0 - 178.6] |
| Alcoholic drinks |  |  |  |  |  |
| Mean (SD) | 92.1 (152.5) | 99.6 (145.8) | 100.1 (145.9) | 96.2 (148.4) | 85.3 (144.5) |
| Median [25th – 75th percentiles] | 34.3 [0.0 - 125.0] | 47.6 [0.0 - 139.2] | 50.0 [0.0 - 137.5] | 42.9 [0.0 - 130.7] | 28.6 [0.0 - 114.3] |
| Meal replacements |  |  |  |  |  |
| Mean (SD) | 1.4 (19.4) | 1.2 (14.7) | 1.5 (17.4) | 1.8 (17.5) | 2.7 (23.0) |
| Median [25th – 75th percentiles] | 0.0 [0.0 - 0.0] | 0.0 [0.0 - 0.0] | 0.0 [0.0 - 0.0] | 0.0 [0.0 - 0.0] | 0.0 [0.0 - 0.0] |
| Savory snacks |  |  |  |  |  |
| Mean (SD) | 9.2 (15.1) | 10.0 (13.6) | 11.0 (13.7) | 12.0 (14.9) | 13.8 (18.0) |
| Median [25th – 75th percentiles] | 2.9 [0.0 - 13.6] | 5.8 [0.0 - 14.5] | 7.1 [0.0 - 16.1] | 7.9 [0.0 - 17.9] | 8.6 [0.0 - 20.4] |

Abbreviations: SD = Standard Deviation

^a^All food group intakes are calculated as the mean intake during the first two years of participation in the study.

## Fig B. Correlations between Schoenfeld residuals and timescale (age, y) from multivariable Cox models between food additive mixtures and type 2 diabetes incidence, NutriNet-Santé cohort, 2009-2023 (n=108,643).

**Mixture 1**


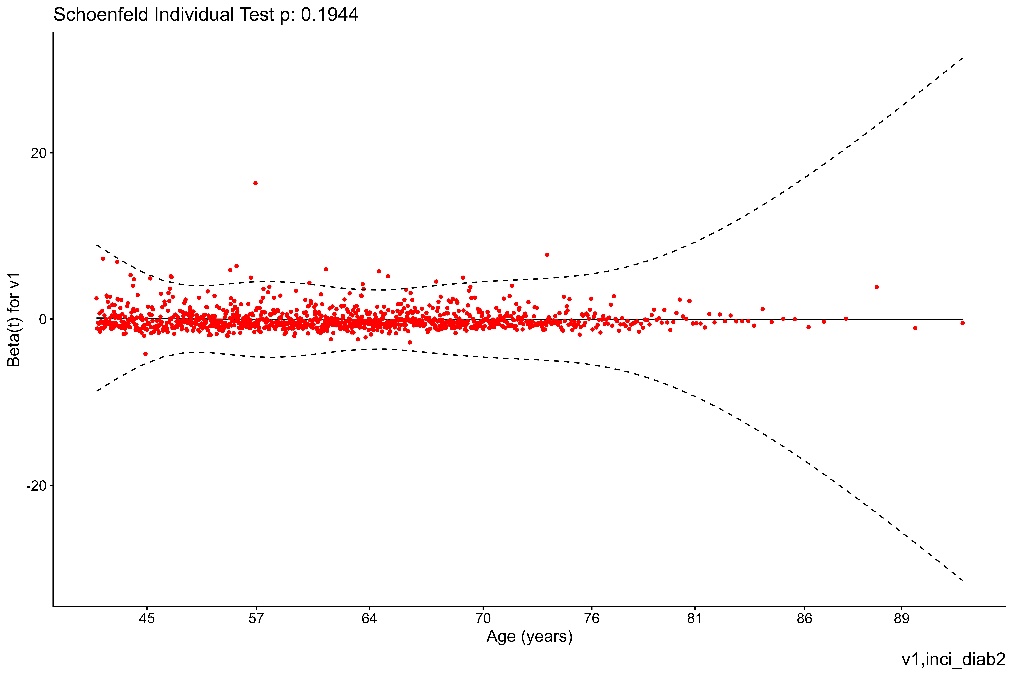


**Mixture 2**

**
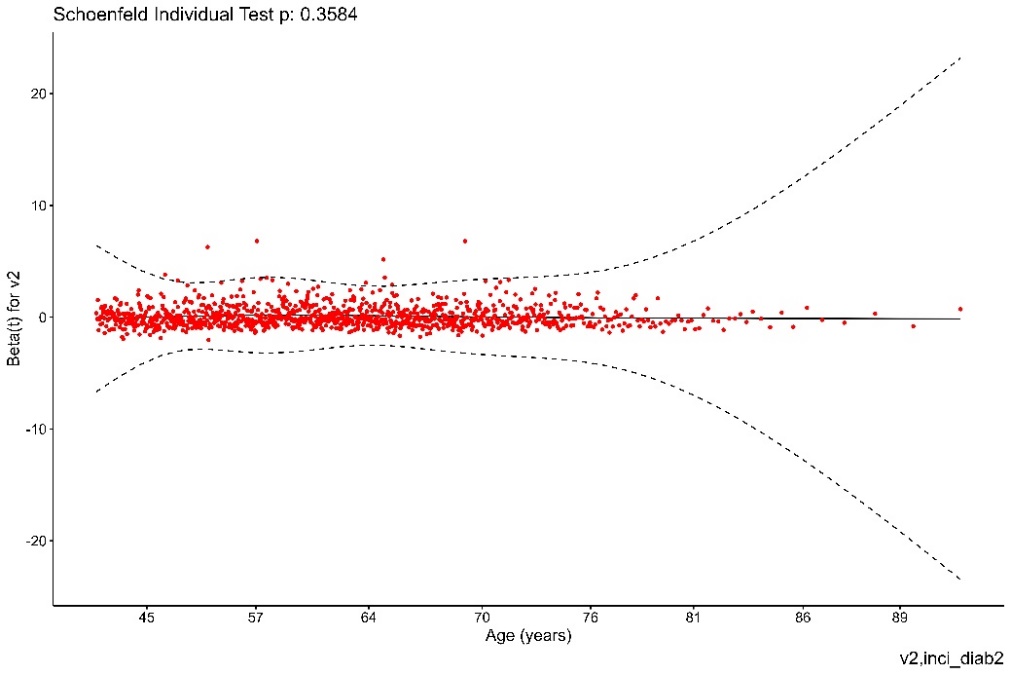
**

**Mixture 3**


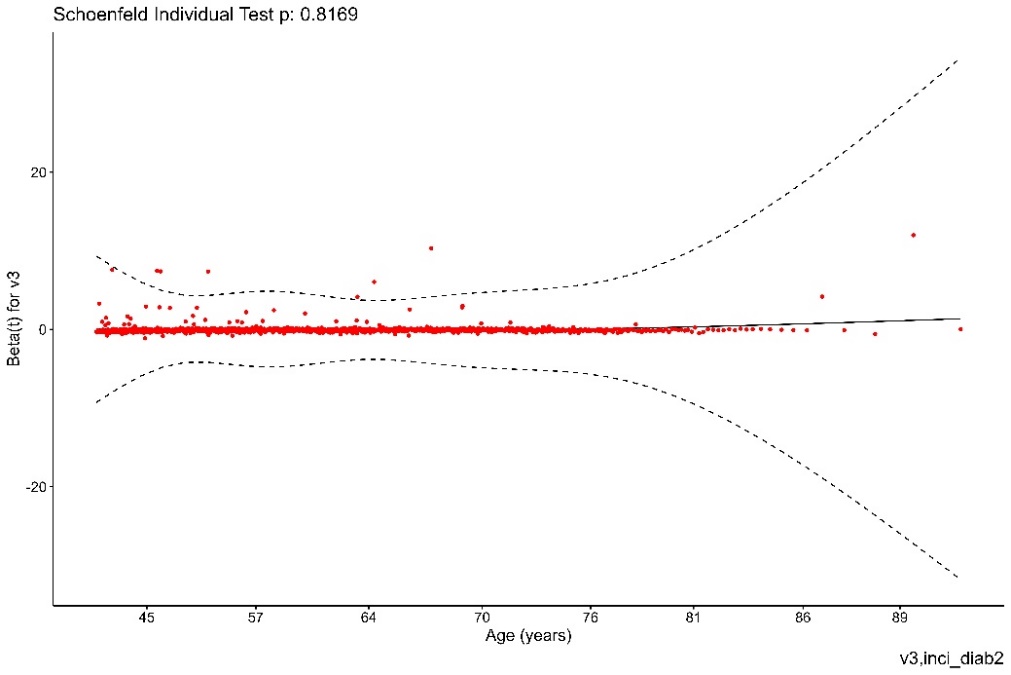


**Mixture 4**


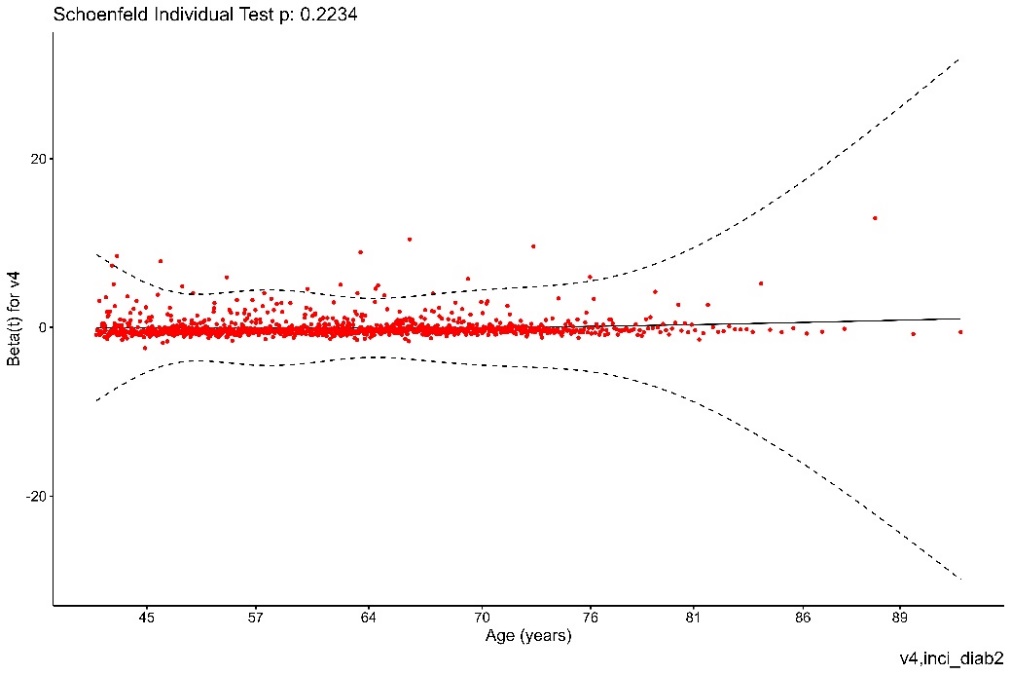


**Mixture 5**


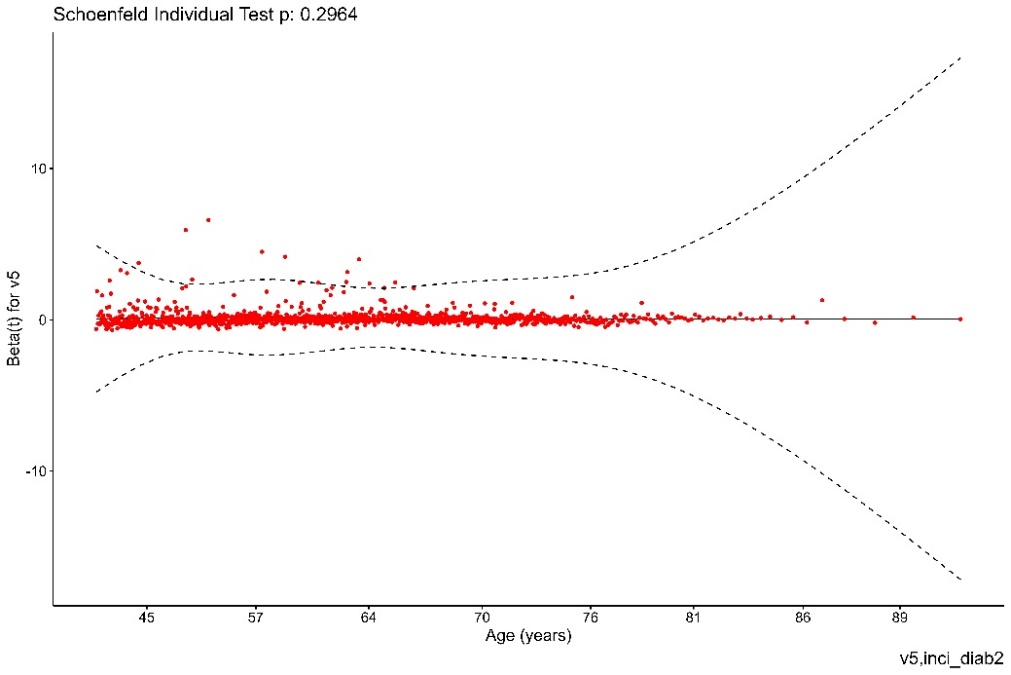


## Fig C. Dose-response associations between food additive mixtures and type 2 diabetes incidence, restricted cubic spline plots, NutriNet-Santé cohort, 2009-2023 (n=108,643 participants ; 1,131 incident cases).

**Mixture 1**


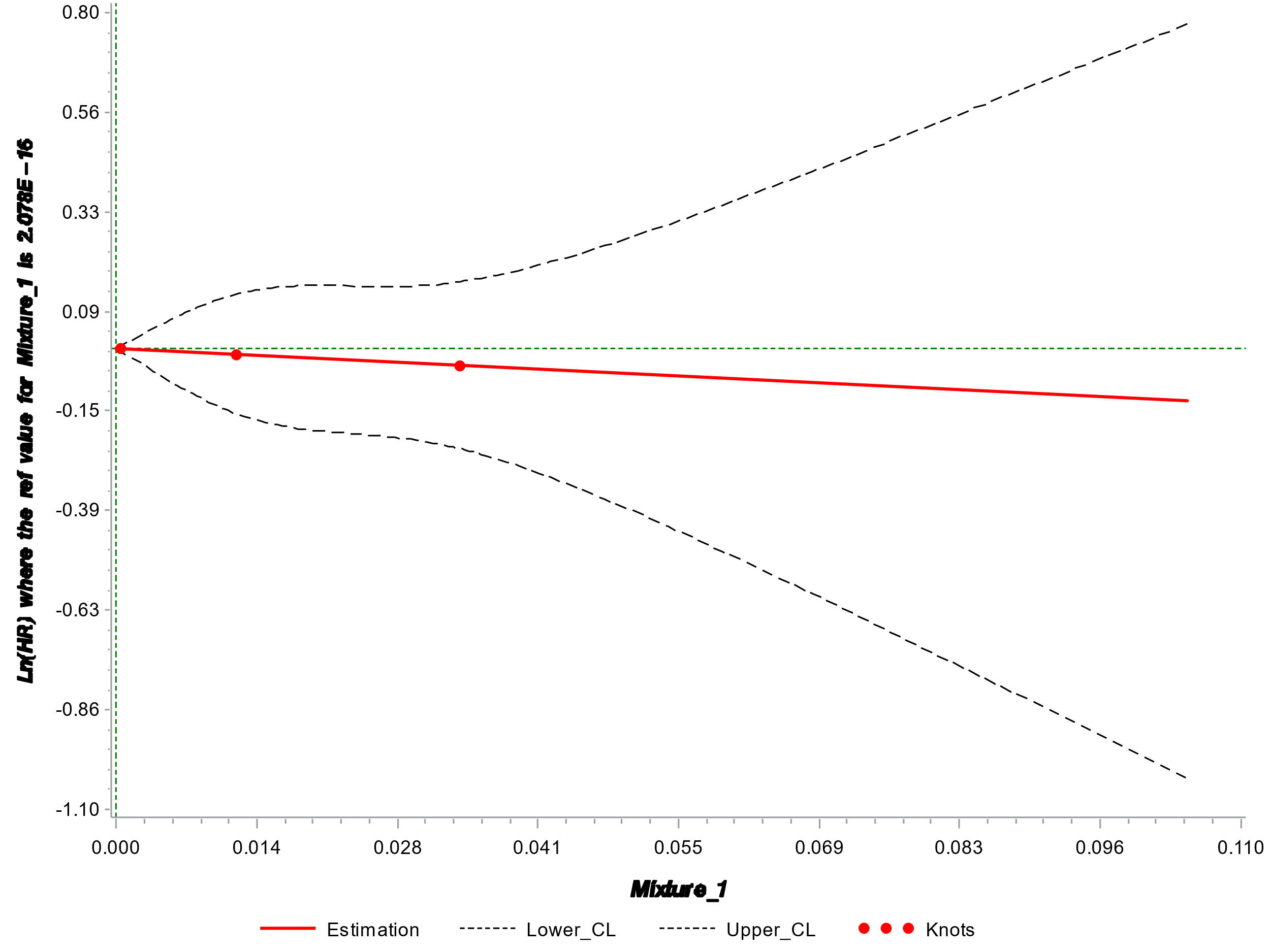


Mixture 1

Log(HR)

P for non-linearity = 0.9

**Mixture 2**


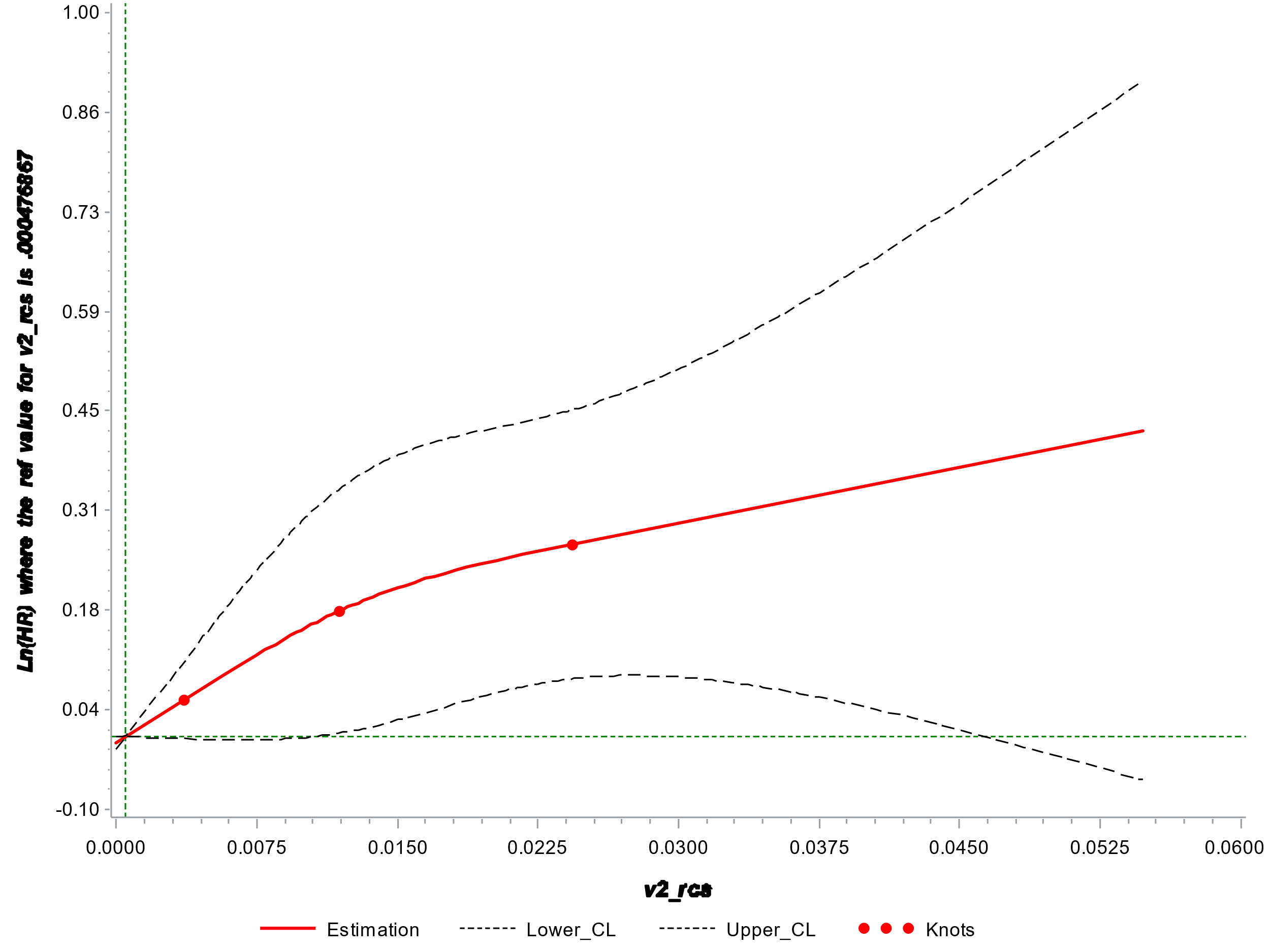


Mixture 2

Log(HR)

P for non-linearity = 0.4

**Mixture 3**


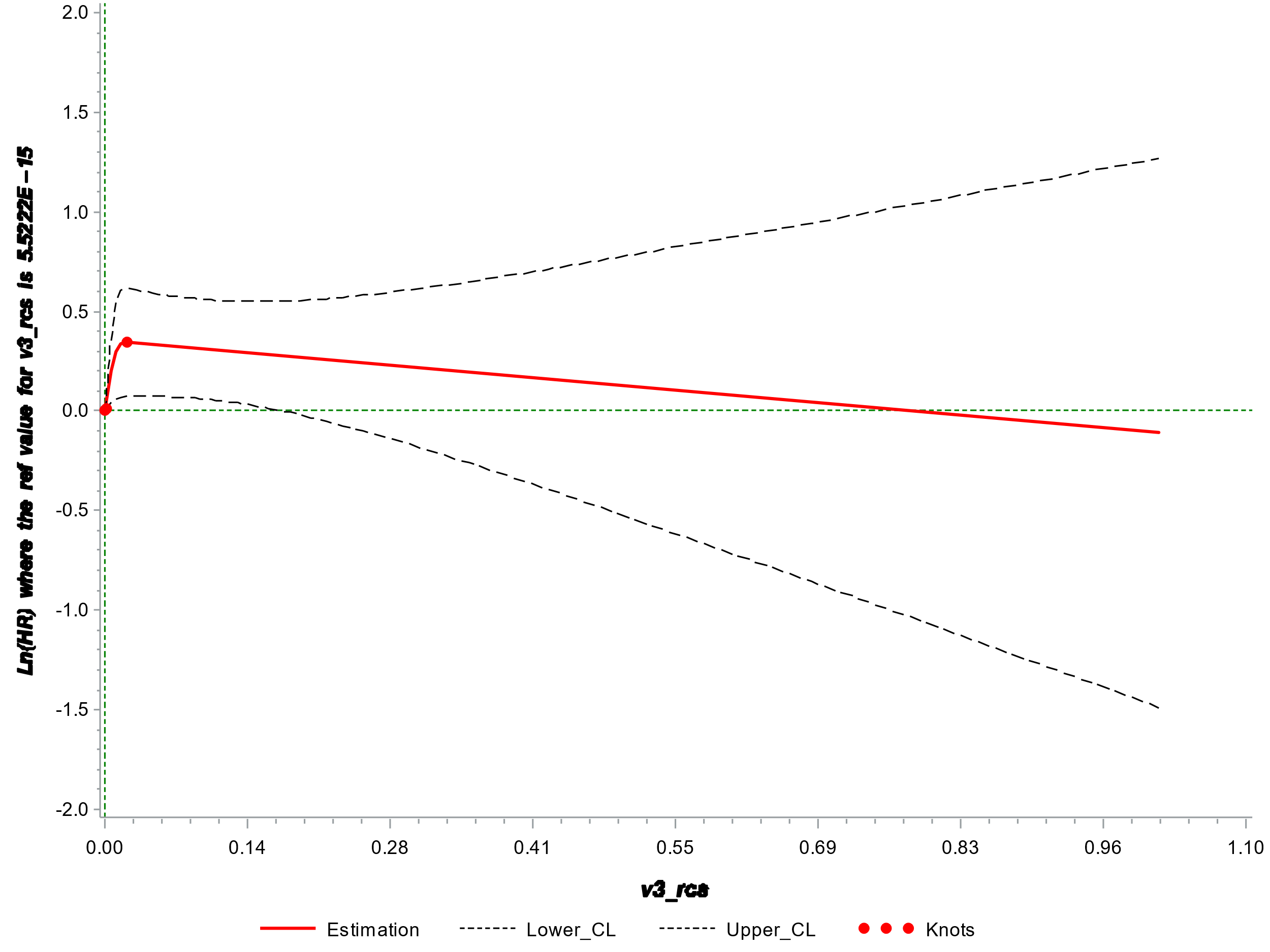


Mixture 3

Log(HR)

P for non-linearity = 0.01

**Mixture 4**


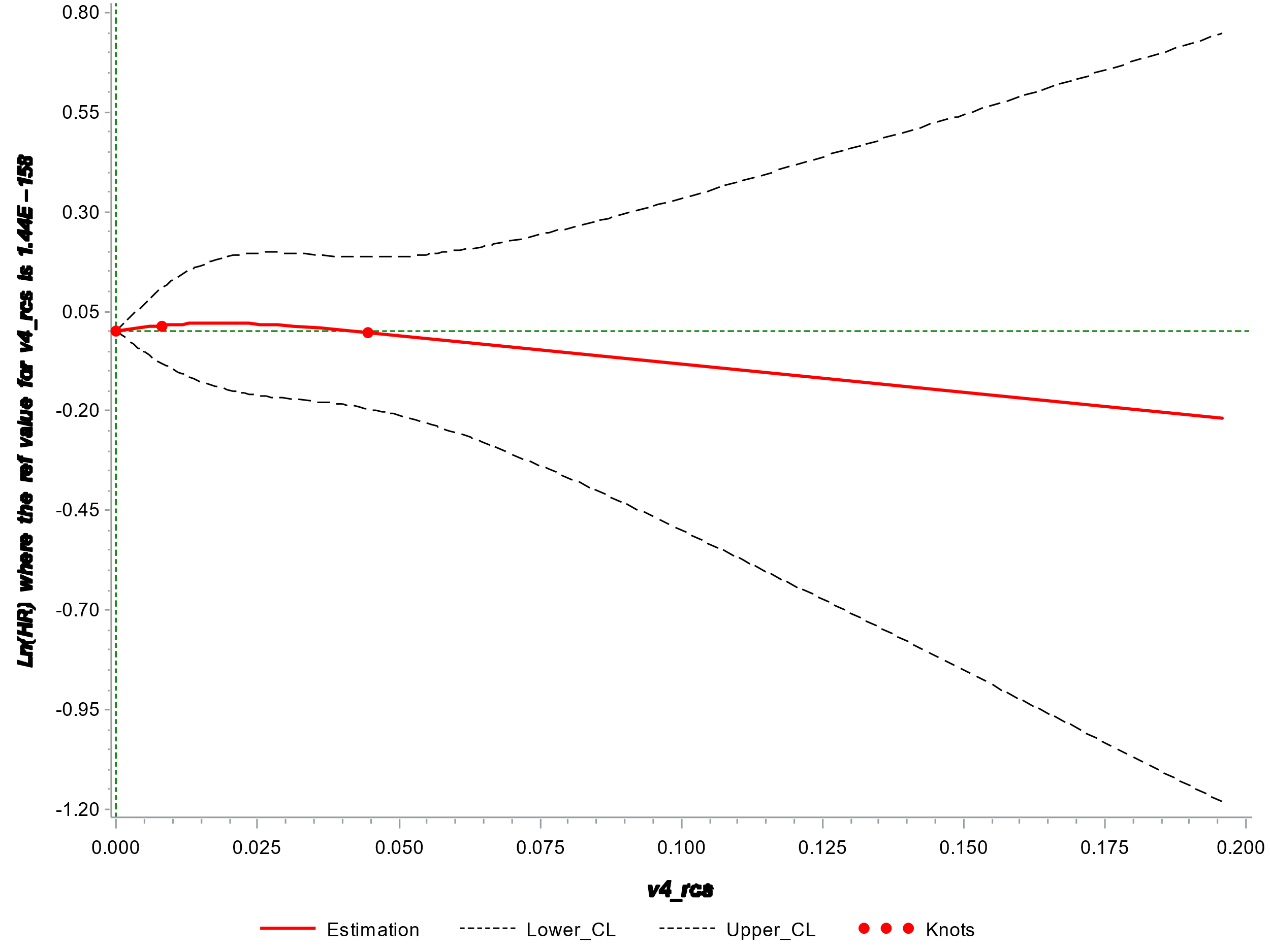


Mixture 4

Log(HR)

P for non-linearity = 0.7

**Mixture 5**


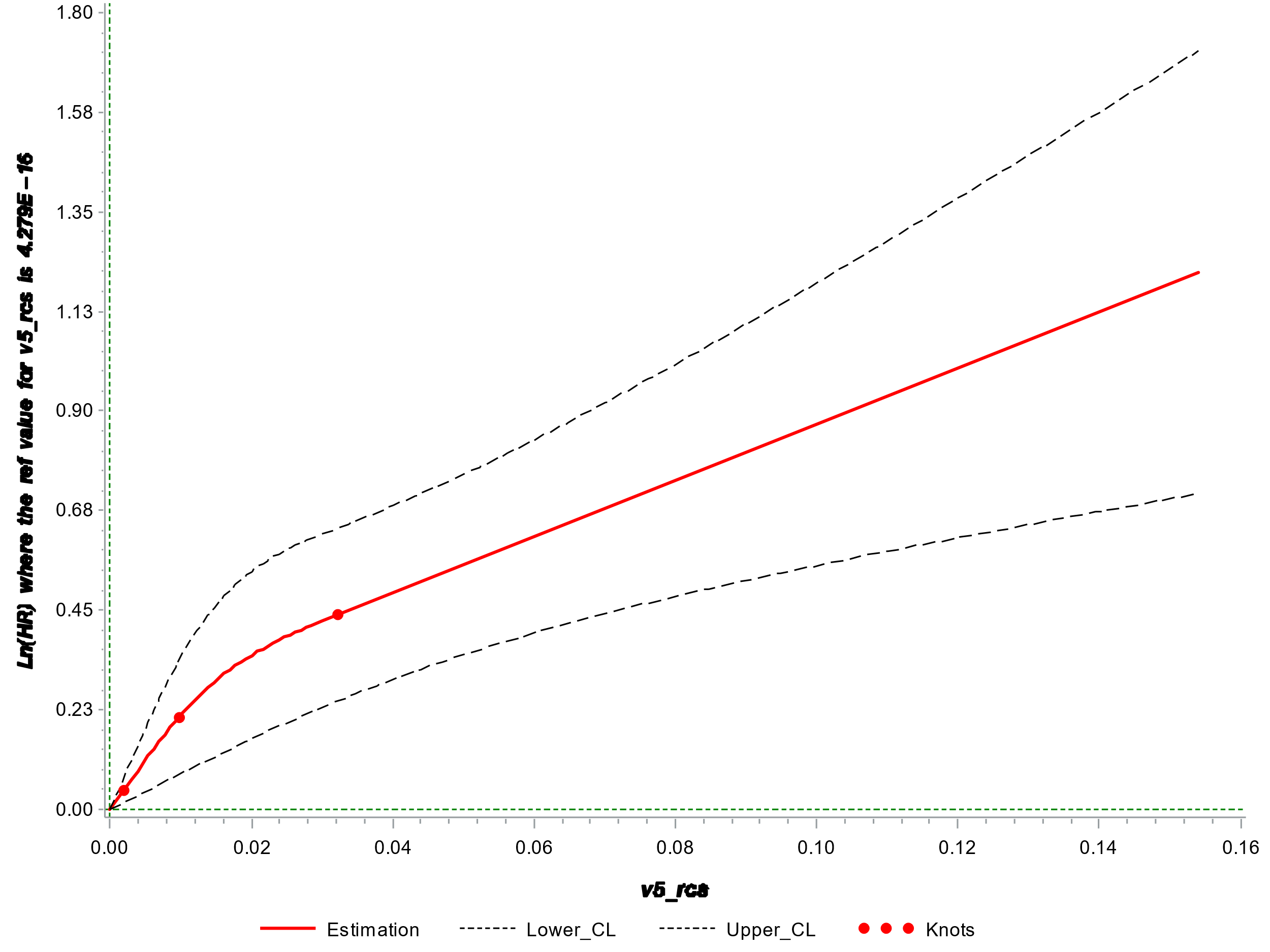


Mixture 5

Log(HR)

P for non-linearity = 0.05

## Table G. Associations between food additive mixtures and type 2 diabetes incidence, NutriNet-Santé cohort, 2009-2023 - Sensitivity analyses

| **Food additive mixtures** | **Models** | **Number of incident cases / number of participants** | **HR for an increment of one SD of the NMF score (95%CI)** | **P value** |
| --- | --- | --- | --- | --- |
| **Mixture 1** | **Model 1** | 927 / 108439 | 0.94 (0.86,1.03) | 0.175 |
|  | **Model 2** | 1131 / 108643 | 0.98 (0.91,1.06) | 0.586 |
|  | **Model 3** | 1131 / 108643 | 0.98 (0.90,1.05) | 0.535 |
|  | **Model 4** | 1131 / 108643 | 0.94 (0.87,1.01) | 0.097 |
|  | **Model 5** | 1131 / 108643 | 0.95 (0.89,1.03) | 0.219 |
|  | **Model 6** | 1131 / 108643 | 0.98 (0.91,1.06) | 0.673 |
|  | **Model 7** | 1131 / 108643 | 0.98 (0.91,1.06) | 0.635 |
| **Mixture 2** | **Model 1** | 927 / 108439 | 1.09 (1.03,1.16) | 0.005 |
|  | **Model 2** | 1131 / 108643 | 1.06 (1.01,1.13) | 0.031 |
|  | **Model 3** | 1131 / 108643 | 1.08 (1.02,1.15) | 0.006 |
|  | **Model 4** | 1131 / 108643 | 1.08 (1.02,1.15) | 0.006 |
|  | **Model 5** | 1131 / 108643 | 1.07 (1.01,1.13) | 0.014 |
|  | **Model 6** | 1131 / 108643 | 1.08 (1.02,1.15) | 0.006 |
|  | **Model 7** | 1131 / 108643 | 1.09 (1.03,1.15) | 0.004 |
| **Mixture 3** | **Model 1** | 927 / 108439 | 1.01 (0.93,1.11) | 0.739 |
|  | **Model 2** | 1131 / 108643 | 1.02 (0.94,1.10) | 0.597 |
|  | **Model 3** | 1131 / 108643 | 1.00 (0.91,1.10) | 0.981 |
|  | **Model 4** | 1131 / 108643 | 0.99 (0.90,1.08) | 0.808 |
|  | **Model 5** | 1131 / 108643 | 0.99 (0.91,1.08) | 0.868 |
|  | **Model 6** | 1131 / 108643 | 1.02 (0.94,1.10) | 0.683 |
|  | **Model 7** | 1131 / 108643 | 1.01 (0.94,1.10) | 0.721 |
| **Mixture 4** | **Model 1** | 927 / 108439 | 0.98 (0.90,1.06) | 0.599 |
|  | **Model 2** | 1131 / 108643 | 0.98 (0.91,1.06) | 0.683 |
|  | **Model 3** | 1131 / 108643 | 0.98 (0.91,1.06) | 0.648 |
|  | **Model 4** | 1131 / 108643 | 0.95 (0.88,1.03) | 0.231 |
|  | **Model 5** | 1131 / 108643 | 0.97 (0.90,1.04) | 0.367 |
|  | **Model 6** | 1131 / 108643 | 0.99 (0.92,1.07) | 0.778 |
|  | **Model 7** | 1131 / 108643 | 0.99 (0.92,1.07) | 0.776 |
| **Mixture 5** | **Model 1** | 927 / 108439 | 1.12 (1.07,1.18) | <0.001 |
|  | **Model 2** | 1131 / 108643 | 1.11 (1.06,1.16) | <0.001 |
|  | **Model 3** | 1131 / 108643 | 1.13 (1.08,1.18) | <0.001 |
|  | **Model 4** | 1131 / 108643 | 1.10 (1.05,1.15) | <0.001 |
|  | **Model 5** | 1131 / 108643 | 1.09 (1.05,1.14) | <0.001 |
|  | **Model 6** | 1131 / 108643 | 1.13 (1.08,1.18) | <0.001 |
|  | **Model 7** | 1131 / 108643 | 1.13 (1.08,1.18) | <0.001 |

Abbreviations: HR: Hazard ratio; SD: Standard Deviation; CI: Confidence Interval.

Mixtures of food additives were derived from non-negative matrix factorisation (NMF, Supplemental eMethod4).

HRs were computed for increments of one SD of each NMF mixture score (SD mixture 1: 12.9; SD mixture 2: 8.0; SD mixture 3: 88.3; SD mixture 4: 20.1; SD mixture 5: 14.7).

All multivariable Cox proportional hazard models were adjusted for (main model): age (time-scale), sex, Body Mass Index (BMI,continuous, kg/m²), physical activity (categorical International Physical Activity Questionnaire (IPAQ) variable: high, moderate, low), smoking status (never smoked, former smoker, current smokers), number of smoked cigarettes in pack-years (continuous), educational level (did not complete secondary education / up to two years of university studies / bachelor degree or higher), socio-professional categories (farmer operator, craftsman/shopkeeper/entrepreneur, managerial staff/intellectual profession, intermediate profession, employee, manual worker, retired, unemployed, student, and other without professional activity), monthly income per household unit (<1200 €/month; 1200–1800 €/month; 1800–2700 €/month; >2700 €/month), family history of type 2 diabetes (yes/no), number of dietary records (continuous), intakes of energy without alcohol (continuous, kcal/d), saturated fatty acids (continuous, g/d), sodium (continuous, mg/d), dietary fibre (continuous, g/d), alcohol (continuous, g/d), and added sugars (continuous, g/d).

Model 1: Main model + excluding cases diagnosed during the first two years to challenge reverse causality. Model 2: Main model + adjustment for diagnosis and/or treatment for at least one prevalent metabolic disorder (i.e. cardiovascular disease. arterial hypertension. hypertriglyceridemia). Model 3: Main model + mutual adjustment for the other NMF food additive mixtures. Model 4: Main model + Healthy and Western dietary patterns derived by factor analysis (continuous). Model 5: Main model with adjustment for food groups instead of nutrients: fruits and vegetables (continuous. g/d), dairy products (continuous. ml/day), red and processed meats (continuous. g/d). Model 6: Main model + indicator of health-seeking behaviours (total number of screening tests or exams in the life course: CT colonography, colonoscopy or sigmoidoscopy, screening test for blood in stools, PAP smear, skin cancer screening exams, mammography, medical breast palpation, PSA blood testing and/or digital rectal examination). Model 7: Main model + region (10 French regions).

## Table H. Association between food additive mixtures coded as tertiles and incidence of type 2 diabetes, NutriNet-Santé cohort, 2009-2023 - Sensitivity analyses.

| Exposure |  | Tertile 1 | Tertile 2 | Tertile 3 | *P*-trend |
| --- | --- | --- | --- | --- | --- |
| Mixture 1 | Participants / Incident cases | 399 / 36215 | 407 / 36214 | 325 / 36214 | 0.977 |
|  | HR (95% CI) | 1 | 1.05 (0.91,1.22) | 1.00 (0.85,1.17) |  |
| Mixture 2 | Participants / Incident cases | 260 / 36215 | 406 / 36214 | 465 / 36214 | 0.007 |
|  | HR (95% CI) | 1 | 1.28 (1.09,1.49) | 1.26 (1.08,1.48) |  |
| Mixture 3 | Participants / Incident cases | 382 / 36215 | 380 / 36214 | 369 / 36214 | 0.586 |
|  | HR (95% CI) | 1 | 0.90 (0.77,1.04) | 0.96 (0.83,1.11) |  |
| Mixture 4 | Participants / Incident cases | 365 / 36215 | 411 / 36214 | 355 / 36214 | 0.346 |
|  | HR (95% CI) | 1 | 1.09 (0.94,1.25) | 1.08 (0.92,1.25) |  |
| Mixture 5 | Participants / Incident cases | 355 / 36215 | 392 / 36214 | 384 / 36214 | 0.001 |
|  | HR (95% CI) | 1 | 0.99 (0.86,1.15) | 1.29 (1.10,1.50) |  |

Abbreviations: HR: Hazard ratio; CI: Confidence Interval.

Mixtures of food additives were derived from non-negative matrix factorisation (NMF, Supplemental eMethod4).

Cut-offs for tertiles were: mixture 1: 1.1 and 9.3; mixture 2: 4.5 and 10.5; mixture 3: 2.1e-11 and 0.1; mixture 4: 0.1 and 5.0; mixture 5: 2.6 and 8.0.

Multivariable Cox proportional hazard models were adjusted for age (time-scale), age (time-scale), sex, Body Mass Index (BMI, continuous, kg/m²), physical activity (categorical International Physical Activity Questionnaire (IPAQ) variable: high, moderate, low), smoking status (never smoked, former smoker, current smokers), number of smoked cigarettes in pack-years (continuous), educational level (did not complete secondary education / up to two years of university studies / bachelor degree or higher), socio-professional categories (farmer operator, craftsman/shopkeeper/entrepreneur, managerial staff/intellectual profession, intermediate profession, employee, manual worker, retired, unemployed, student, and other without professional activity), monthly income per household unit (<1200 €/month; 1200–1800 €/month; 1800–2700 €/month; >2700 €/month), number of dietary records (continuous), intakes of energy without alcohol (continuous, kcal/d), saturated fatty acids (continuous, g/d), sodium (continuous, mg/d), dietary fibre (continuous, g/d), alcohol (continuous, g/d), and added sugars (continuous, g/d).

# Table I. Associations between food additive mixtures and incidence of type 2 diabetes, stratified by the Programme National Nutrition Santé – Guidelines Score 2 (PNNS-GS2), NutriNet-Santé cohort, 2009-2023 - Sensitivity analyses

### Below the sex-specific median

| **Exposure** | **HR for an increment of one SD of the NMF score (95%CI)** | **P-value** | **Number of incident cases / number of participants** |
| --- | --- | --- | --- |
| Mixture 1 | 0.98 (0.89, 1.08) | 0.6 | 732 / 62225 |
| Mixture 2 | 1.07 (0.99, 1.15) | 0.07 | 732 / 62225 |
| Mixture 3 | 1.01 (0.90, 1.14) | 0.8 | 732 / 62225 |
| Mixture 4 | 0.97 (0.89, 1.07) | 0.6 | 732 / 62225 |
| Mixture 5 | 1.13 (1.07, 1.20) | <0.001 | 732 / 62225 |

### Above the sex-specific median

| **Exposure** | **HR for an increment of one SD of the NMF score (95%CI)** | **P-value** | **Number of incident cases / number of participants** |
| --- | --- | --- | --- |
| Mixture 1 | 0.97 (0.86, 1.10) | 0.6 | 399 / 46418 |
| Mixture 2 | 1.09 (0.99, 1.20) | 0.07 | 399 / 46418 |
| Mixture 3 | 1.02 (0.93, 1.12) | 0.7 | 399 / 46418 |
| Mixture 4 | 1.00 (0.89, 1.14) | 0.9 | 399 / 46418 |
| Mixture 5 | 1.10 (1.04, 1.16) | 0.001 | 399 / 46418 |

Abbreviations: HR: Hazard ratio; SD: Standard Deviation, NMF: Non-Negative Matrix Factorisation, CI: Confidence Interval.

Models were adjusted for age (time-scale), sex, , Body Mass Index (BMI, continuous, kg/m²), physical activity (categorical International Physical Activity Questionnaire (IPAQ) variable: high, moderate, low), smoking status (never smoked, former smoker, current smokers), number of smoked cigarettes in pack-years (continuous), educational level (did not complete secondary education / up to two years of university studies / bachelor degree or higher), socio-professional categories (farmer operator, craftsman/shopkeeper/entrepreneur, managerial staff/intellectual profession, intermediate profession, employee, manual worker, retired, unemployed, student, and other without professional activity), monthly income per household unit (<1200 €/month; 1200–1800 €/month; 1800–2700 €/month; >2700 €/month), family history of type 2 diabetes (yes/no), number of dietary records (continuous), intakes of energy without alcohol (continuous, kcal/d), saturated fatty acids (continuous, g/d), sodium (continuous, mg/d), dietary fibre (continuous, g/d), alcohol (continuous, g/d), and added sugars (continuous, g/d).

No interaction was detected between the Programme National Nutrition Santé – Guidelines Score 2 (PNNS-GS2) score and neither mixture 2 (p interaction=0.8) nor mixture 5 (p interaction=0.6).

## Table J. Association between mixtures 2 and 5 and type 2 diabetes incidence adjusted for the key food additives contributing to each mixture (residual method), NutriNet-Santé cohort, 2009-2023 (n=108,643 participants; 1,131 incident cases) - Sensitivity analyses

| **Mixture 2** | | **Mixture 5** | |
| --- | --- | --- | --- |
| Residuals after regression on the following additives: | **HR for an increment of one SD of the residual NMF score (95%CI)** | Residuals after regression on the following additives: | **HR for an increment of one SD of the residual NMF score (95%CI)** |
| Modified starches | 1.06 (1.00, 1.12) | E 330 Citric acid | 1.10 (1.04, 1.16) |
| E 440 Pectins | 1.07 (1.01, 1.13) | E331 Sodium citrates | 1.09 (1.05, 1.14) |
| E 412 Guar gum | 1.05 (0.99, 1.12) | E338 Phosphoric acid | 1.09 (1.05, 1.14) |
| E 407 Carrageenan | 1.08 (1.02, 1.14) | E150d Sulphite ammonia caramel | 1.09 (1.05, 1.14) |
| E452 Polyphosphates | 1.08 (1.02, 1.14) | E950 Acesulfame K | 1.08 (1.03, 1.14) |
| E202 Potassium sorbate | 1.06 (1.00, 1.13) | E951 Aspartame | 1.10 (1.05, 1.15) |
| E100 Curcumin | 1.07 (1.01, 1.13) | E955 Sucralose | 1.13 (1.08, 1.18) |
| E415 Xanthan gum | 1.07 (1.01, 1.13) | E414 Arabic gum | 1.13 (1.08, 1.18) |
|  |  | E296 Malic acid | 1.12 (1.08, 1.17) |
|  |  | E903 Carnauba wax | 1.12 (1.08, 1.17) |
|  |  | E160c Paprika extract. capsanthin. capsorubin | 1.12 (1.08, 1.17) |
|  |  | E163 Anthocyanins | 1.12 (1.08, 1.17) |
|  |  | E412 Guar gum | 1.12 (1.07, 1.17) |
|  |  | E440 Pectins | 1.13 (1.08, 1.17) |

Abbreviations: HR: Hazard ratio; CI: Confidence Interval.

Mixtures of food additives were derived from non-negative matrix factorisation (NMF, Supplemental eMethod4). Mixtures 2 and 5 were regressed (linear regression models) on each main additive characteristic of the mixture and residuals were extracted. HRs were computed for increments of one SD of each NMF mixture score residuals (SD residual mixture 2: 8.0; SD residual mixture 5: 14.7).

Multivariable Cox proportional hazard models were adjusted for age (time-scale), Sex, Body Mass Index (BMI, continuous, kg/m²), physical activity (categorical International Physical Activity Questionnaire (IPAQ) variable: high, moderate, low), smoking status (never smoked, former smoker, current smokers), number of smoked cigarettes in pack-years (continuous), socio-professional categories (farmer, craftsman/shopkeeper/entrepreneur, managerial staff/intellectual profession, intermediate profession, employee, manual worker, retired, unemployed, student, and other without professional activity), monthly income per household unit (<1200 €/month; 1200–1800 €/month; 1800–2700 €/month; >2700 €/month), intakes of energy without alcohol (continuous, kcal/d), total saturated fatty acids (continuous, g/d), sodium (continuous, mg/d), dietary fibre (continuous, g/d), alcohol (continuous, g/d), added sugars (continuous, g/d).

## Table K. Interactions between the key food additives contributing to mixtures 2 and 5, NutriNet-Santé cohort, 2009-2023 (n=108,643 participants; 1,131 incident cases)

### A. Mixture 2

###

|  |  |  | **IC95%** | |  | **IC95%** | |
| --- | --- | --- | --- | --- | --- | --- | --- |
| **Interaction** | **P-value** | **Regression coefficient beta** | **Lower** | **Upper** | **Regression coefficient beta after standardization** | **Lower** | **Upper** |
| E202 | <0,001 | 7,78E-03 | 4,21E-03 | 1,14E-02 | 1,77E-01 | 1,08E-01 | 2,46E-01 |
| E412 : E415 | 0,002 | -1,08E-06 | -1,76E-06 | -4,05E-07 | -5,55E-02 | -9,03E-02 | -2,07E-02 |
| E100 | 0,002 | 8,12E-02 | 2,99E-02 | 1,33E-01 | 1,03E-01 | 3,11E-02 | 1,76E-01 |
| E440 : E415 | 0,011 | 8,56E-07 | 1,94E-07 | 1,52E-06 | 5,74E-02 | 1,30E-02 | 1,02E-01 |
| Modified Starches : E100 | 0,013 | -2,90E-05 | -5,20E-05 | -6,12E-06 | -7,65E-02 | -1,37E-01 | -1,61E-02 |
| Modified Starches : E412 | 0,019 | 2,46E-07 | 3,97E-08 | 4,51E-07 | 6,60E-02 | 1,07E-02 | 1,21E-01 |
| E202 : E100 | 0,033 | 5,18E-04 | 4,12E-05 | 9,95E-04 | 3,44E-02 | 2,74E-03 | 6,61E-02 |
| E452 | 0,045 | 1,13E-03 | 2,33E-05 | 2,24E-03 | 8,48E-02 | -1,39E-02 | 1,84E-01 |
| Modified Starches : E202 | 0,062 | -1,36E-06 | -2,79E-06 | 6,68E-08 | -4,66E-02 | -9,56E-02 | 2,29E-03 |
| E440 : E412 | 0,067 | -4,24E-07 | -8,77E-07 | 2,93E-08 | -2,95E-02 | -6,11E-02 | 2,04E-03 |
| E100 : E415 | 0,097 | -1,01E-04 | -2,21E-04 | 1,82E-05 | -5,08E-02 | -1,11E-01 | 9,11E-03 |
| E412 | 0,105 | 4,73E-04 | -9,89E-05 | 1,04E-03 | 1,22E-01 | 3,71E-02 | 2,08E-01 |
| E407 : E452 | 0,158 | -3,97E-06 | -9,49E-06 | 1,55E-06 | -5,68E-02 | -1,36E-01 | 2,22E-02 |
| E415 | 0,200 | 3,84E-04 | -2,03E-04 | 9,72E-04 | 5,94E-02 | -3,13E-02 | 1,50E-01 |
| E440 : E407 | 0,218 | -1,02E-06 | -2,63E-06 | 5,99E-07 | -3,27E-02 | -8,47E-02 | 1,93E-02 |
| E407 | 0,247 | 6,18E-04 | -4,28E-04 | 1,66E-03 | 3,23E-02 | -4,04E-02 | 1,05E-01 |
| E407 : E100 | 0,330 | 4,93E-05 | -5,00E-05 | 1,49E-04 | 1,19E-02 | -1,20E-02 | 3,58E-02 |
| E452 : E202 | 0,374 | -2,92E-06 | -9,37E-06 | 3,53E-06 | -1,16E-02 | -3,70E-02 | 1,39E-02 |
| E440 : E202 | 0,454 | -2,29E-06 | -8,29E-06 | 3,71E-06 | -2,04E-02 | -7,37E-02 | 3,30E-02 |
| E440 | 0,561 | 1,20E-04 | -2,85E-04 | 5,26E-04 | 1,74E-02 | -6,78E-02 | 1,03E-01 |
| E412 : E452 | 0,579 | -5,94E-07 | -2,69E-06 | 1,50E-06 | -1,84E-02 | -8,33E-02 | 4,65E-02 |
| Modified Starches : E452 | 0,582 | -9,40E-08 | -4,28E-07 | 2,40E-07 | -1,47E-02 | -6,72E-02 | 3,77E-02 |
| E440 : E452 | 0,613 | 3,96E-07 | -1,14E-06 | 1,93E-06 | 1,61E-02 | -4,62E-02 | 7,84E-02 |
| E407 : E202 | 0,689 | 3,07E-06 | -1,20E-05 | 1,81E-05 | 9,63E-03 | -3,76E-02 | 5,68E-02 |
| Modified Starches | 0,751 | -1,31E-05 | -9,40E-05 | 6,78E-05 | -2,25E-02 | -9,00E-02 | 4,50E-02 |
| E407 : E415 | 0,782 | -3,19E-07 | -2,58E-06 | 1,94E-06 | -7,54E-03 | -6,09E-02 | 4,58E-02 |
| E452 : E415 | 0,789 | -3,71E-07 | -3,09E-06 | 2,34E-06 | -1,11E-02 | -9,21E-02 | 7,00E-02 |
| E440 : E100 | 0,798 | 7,22E-06 | -4,80E-05 | 6,24E-05 | 4,93E-03 | -3,28E-02 | 4,26E-02 |
| E452 : E100 | 0,802 | 2,31E-05 | -1,58E-04 | 2,04E-04 | 7,03E-03 | -4,79E-02 | 6,20E-02 |
| E412 : E407 | 0,823 | -2,51E-07 | -2,46E-06 | 1,96E-06 | -6,17E-03 | -6,04E-02 | 4,80E-02 |
| E412 : E100 | 0,854 | 9,24E-06 | -8,93E-05 | 1,08E-04 | 4,81E-03 | -4,65E-02 | 5,61E-02 |
| Modified Starches : E440 | 0,856 | -1,32E-08 | -1,56E-07 | 1,29E-07 | -4,67E-03 | -5,49E-02 | 4,56E-02 |
| Modified Starches : E415 | 0,870 | -1,67E-08 | -2,17E-07 | 1,84E-07 | -4,33E-03 | -5,62E-02 | 4,75E-02 |
| Modified Starches : E407 | 0,875 | 3,02E-08 | -3,47E-07 | 4,07E-07 | 3,75E-03 | -4,31E-02 | 5,06E-02 |
| E412 : E202 | 0,896 | 2,74E-07 | -3,85E-06 | 4,40E-06 | 1,86E-03 | -2,61E-02 | 2,98E-02 |
| E202 : E415 | 0,948 | 9,95E-08 | -2,87E-06 | 3,07E-06 | 6,50E-04 | -1,88E-02 | 2,01E-02 |

###

### B. Mixture 5

|  |  |  | **IC95%** | |  | **IC95%** | |
| --- | --- | --- | --- | --- | --- | --- | --- |
| **Interaction** | **P-value** | **Regression coefficient beta** | **Lower** | **Upper** | **Regression coefficient beta after standardization** | **Lower** | **Upper** |
| E412 | 0,001 | 5,93E-04 | 2,50E-04 | 9,36E-04 | 7,72E-02 | 1,15E-02 | 1,43E-01 |
| E331 | 0,016 | 1,00E-03 | 1,86E-04 | 1,82E-03 | 1,69E-01 | -3,59E-03 | 3,42E-01 |
| E163 | 0,032 | 2,61E-02 | 2,29E-03 | 4,99E-02 | 1,44E-01 | 1,48E-02 | 2,72E-01 |
| E330 : E160c | 0,041 | 1,01E-04 | 4,34E-06 | 1,99E-04 | 2,64E-02 | 1,13E-03 | 5,17E-02 |
| E296 : E903 | 0,051 | 3,07E-04 | -8,47E-07 | 6,15E-04 | 4,79E-02 | -1,32E-04 | 9,60E-02 |
| E950 : E955 | 0,053 | -4,29E-04 | -8,63E-04 | 5,13E-06 | -8,52E-02 | -1,71E-01 | 1,02E-03 |
| E951 : E903 | 0,064 | 8,35E-04 | -4,78E-05 | 1,72E-03 | 7,07E-02 | -4,04E-03 | 1,45E-01 |
| E950 : E414 | 0,076 | 3,15E-05 | -3,29E-06 | 6,62E-05 | 1,92E-01 | -2,01E-02 | 4,04E-01 |
| E903 : E163 | 0,077 | -1,45E-03 | -3,05E-03 | 1,56E-04 | -2,97E-02 | -6,27E-02 | 3,21E-03 |
| E160c : E440 | 0,077 | -2,44E-04 | -5,15E-04 | 2,65E-05 | -3,61E-02 | -7,62E-02 | 3,92E-03 |
| E950 : E160c | 0,082 | 9,14E-03 | -1,17E-03 | 1,95E-02 | 6,23E-02 | -7,98E-03 | 1,33E-01 |
| E414 : E440 | 0,084 | 6,68E-07 | -8,90E-08 | 1,42E-06 | 8,84E-02 | -1,18E-02 | 1,89E-01 |
| E951 : E296 | 0,091 | -5,80E-05 | -1,25E-04 | 9,16E-06 | -9,71E-02 | -2,09E-01 | 1,53E-02 |
| E296 | 0,095 | 1,89E-03 | -3,30E-04 | 4,12E-03 | 7,96E-02 | 3,27E-04 | 1,59E-01 |
| E330 | 0,098 | 1,06E-04 | -1,98E-05 | 2,32E-04 | 3,64E-02 | -2,04E-02 | 9,33E-02 |
| E955 : E163 | 0,102 | 3,28E-04 | -6,54E-05 | 7,22E-04 | 3,43E-02 | -6,82E-03 | 7,53E-02 |
| E903 | 0,110 | 6,08E-02 | -1,38E-02 | 1,35E-01 | 6,57E-02 | -9,08E-02 | 2,22E-01 |
| E951 : E160c | 0,118 | -2,38E-03 | -5,37E-03 | 6,02E-04 | -3,52E-02 | -7,92E-02 | 8,87E-03 |
| E331 : E163 | 0,158 | -4,28E-05 | -1,02E-04 | 1,65E-05 | -8,38E-02 | -2,00E-01 | 3,24E-02 |
| E903 : E412 | 0,161 | -1,61E-04 | -3,86E-04 | 6,39E-05 | -1,04E-01 | -2,50E-01 | 4,14E-02 |
| E330 : E331 | 0,163 | -3,10E-07 | -7,45E-07 | 1,25E-07 | -4,41E-02 | -1,06E-01 | 1,78E-02 |
| E330 : E903 | 0,166 | -4,47E-05 | -1,08E-04 | 1,85E-05 | -6,69E-02 | -1,61E-01 | 2,77E-02 |
| E951 | 0,171 | 2,31E-03 | -1,00E-03 | 5,63E-03 | 4,05E-02 | -4,36E-02 | 1,25E-01 |
| E331 : E440 | 0,175 | -6,61E-07 | -1,62E-06 | 2,94E-07 | -5,35E-02 | -1,31E-01 | 2,38E-02 |
| E903 : E160c | 0,198 | -9,92E-02 | -2,50E-01 | 5,17E-02 | -1,36E-01 | -3,44E-01 | 7,11E-02 |
| E955 : E903 | 0,212 | 2,37E-03 | -1,36E-03 | 6,10E-03 | 9,51E-02 | -5,44E-02 | 2,45E-01 |
| E951 : E414 | 0,238 | -1,51E-05 | -4,02E-05 | 9,96E-06 | -1,99E-01 | -5,30E-01 | 1,31E-01 |
| E163 : E412 | 0,254 | -3,47E-05 | -9,43E-05 | 2,49E-05 | -5,86E-02 | -1,59E-01 | 4,20E-02 |
| E903 : E440 | 0,280 | 7,00E-05 | -5,71E-05 | 1,97E-04 | 5,95E-02 | -4,85E-02 | 1,67E-01 |
| E414 : E903 | 0,280 | -3,62E-05 | -1,02E-04 | 2,95E-05 | -4,45E-02 | -1,25E-01 | 3,63E-02 |
| E330 : E296 | 0,306 | -1,27E-06 | -3,71E-06 | 1,16E-06 | -3,76E-02 | -1,10E-01 | 3,44E-02 |
| E955 : E296 | 0,310 | 4,56E-05 | -4,25E-05 | 1,34E-04 | 3,61E-02 | -3,37E-02 | 1,06E-01 |
| E331 : E160c | 0,312 | -3,37E-04 | -9,92E-04 | 3,17E-04 | -4,42E-02 | -1,30E-01 | 4,15E-02 |
| E330 : E951 | 0,317 | 1,18E-06 | -1,13E-06 | 3,49E-06 | 1,89E-02 | -1,82E-02 | 5,60E-02 |
| E331 : E296 | 0,328 | 2,21E-06 | -2,21E-06 | 6,63E-06 | 3,28E-02 | -3,29E-02 | 9,85E-02 |
| E338 : E951 | 0,331 | -2,84E-04 | -8,56E-04 | 2,88E-04 | -3,80E-01 | -1,15E+00 | 3,87E-01 |
| E150d : E951 | 0,338 | 2,60E-05 | -2,71E-05 | 7,91E-05 | 3,71E-01 | -3,87E-01 | 1,13E+00 |
| E414 : E412 | 0,346 | -8,65E-07 | -2,66E-06 | 9,34E-07 | -8,74E-02 | -2,69E-01 | 9,43E-02 |
| E950 : E903 | 0,354 | -1,94E-03 | -6,03E-03 | 2,16E-03 | -7,58E-02 | -2,36E-01 | 8,43E-02 |
| E950 | 0,360 | 7,75E-03 | -8,85E-03 | 2,43E-02 | 1,56E-01 | -3,14E-02 | 3,43E-01 |
| E296 : E412 | 0,369 | -2,62E-06 | -8,33E-06 | 3,09E-06 | -3,35E-02 | -1,07E-01 | 3,96E-02 |
| E331 : E414 | 0,377 | 5,05E-07 | -6,16E-07 | 1,63E-06 | 5,92E-02 | -7,22E-02 | 1,91E-01 |
| E955 : E160c | 0,424 | -6,45E-03 | -2,23E-02 | 9,37E-03 | -4,50E-02 | -1,55E-01 | 6,54E-02 |
| E414 | 0,426 | 2,26E-04 | -3,30E-04 | 7,81E-04 | 1,08E-03 | -1,88E-01 | 1,90E-01 |
| E296 : E160c | 0,463 | 3,52E-04 | -5,88E-04 | 1,29E-03 | 9,57E-03 | -1,60E-02 | 3,51E-02 |
| E330 : E412 | 0,465 | -1,76E-07 | -6,46E-07 | 2,95E-07 | -2,15E-02 | -7,93E-02 | 3,62E-02 |
| E955 | 0,466 | 4,58E-03 | -7,74E-03 | 1,69E-02 | 7,96E-02 | -6,44E-02 | 2,24E-01 |
| E331 : E955 | 0,481 | -9,02E-06 | -3,41E-05 | 1,61E-05 | -3,44E-02 | -1,30E-01 | 6,14E-02 |
| E951 : E412 | 0,520 | 2,14E-06 | -4,38E-06 | 8,66E-06 | 1,49E-02 | -3,04E-02 | 6,02E-02 |
| E955 : E440 | 0,523 | 4,73E-06 | -9,78E-06 | 1,92E-05 | 2,04E-02 | -4,22E-02 | 8,30E-02 |
| E955 : E412 | 0,524 | 6,59E-06 | -1,37E-05 | 2,68E-05 | 2,17E-02 | -4,50E-02 | 8,83E-02 |
| E160c : E412 | 0,549 | 1,19E-04 | -2,71E-04 | 5,09E-04 | 1,35E-02 | -3,05E-02 | 5,74E-02 |
| E331 : E951 | 0,560 | -2,60E-06 | -1,14E-05 | 6,16E-06 | -2,10E-02 | -9,16E-02 | 4,97E-02 |
| E331 : E950 | 0,567 | 3,21E-06 | -7,78E-06 | 1,42E-05 | 1,20E-02 | -2,90E-02 | 5,29E-02 |
| E412 : E440 | 0,572 | -1,06E-07 | -4,72E-07 | 2,61E-07 | -7,36E-03 | -3,29E-02 | 1,82E-02 |
| E163 : E440 | 0,619 | 9,45E-06 | -2,78E-05 | 4,67E-05 | 2,09E-02 | -6,15E-02 | 1,03E-01 |
| E440 | 0,631 | 8,79E-05 | -2,71E-04 | 4,47E-04 | 2,93E-02 | -5,65E-02 | 1,15E-01 |
| E150d : E903 | 0,661 | 2,96E-04 | -1,03E-03 | 1,62E-03 | 3,94E-01 | -1,37E+00 | 2,16E+00 |
| E950 : E163 | 0,674 | 2,99E-04 | -1,09E-03 | 1,69E-03 | 3,05E-02 | -1,11E-01 | 1,72E-01 |
| E338 : E296 | 0,683 | 1,16E-04 | -4,39E-04 | 6,71E-04 | 2,86E-01 | -1,09E+00 | 1,66E+00 |
| E296 : E163 | 0,687 | 4,17E-05 | -1,61E-04 | 2,44E-04 | 1,69E-02 | -6,54E-02 | 9,93E-02 |
| E338 : E163 | 0,696 | 8,37E-04 | -3,36E-03 | 5,03E-03 | 2,72E-01 | -1,09E+00 | 1,64E+00 |
| E414 : E163 | 0,701 | -7,81E-06 | -4,76E-05 | 3,20E-05 | -2,50E-02 | -1,53E-01 | 1,03E-01 |
| E338 : E903 | 0,707 | -2,73E-03 | -1,69E-02 | 1,15E-02 | -3,41E-01 | -2,12E+00 | 1,43E+00 |
| E950 : E951 | 0,707 | -2,72E-05 | -1,69E-04 | 1,15E-04 | -1,14E-02 | -7,09E-02 | 4,80E-02 |
| E331 : E150d | 0,717 | -1,19E-06 | -7,64E-06 | 5,26E-06 | -1,51E-01 | -9,69E-01 | 6,67E-01 |
| E951 : E955 | 0,718 | 2,38E-05 | -1,06E-04 | 1,53E-04 | 1,02E-02 | -4,54E-02 | 6,59E-02 |
| E150d : E163 | 0,719 | -7,18E-05 | -4,63E-04 | 3,20E-04 | -2,49E-01 | -1,61E+00 | 1,11E+00 |
| E150d : E296 | 0,720 | -9,50E-06 | -6,13E-05 | 4,24E-05 | -2,50E-01 | -1,61E+00 | 1,11E+00 |
| E338 : E440 | 0,730 | 1,35E-05 | -6,31E-05 | 9,01E-05 | 1,81E-01 | -8,48E-01 | 1,21E+00 |
| E951 : E440 | 0,733 | 8,18E-07 | -3,89E-06 | 5,53E-06 | 7,45E-03 | -3,54E-02 | 5,03E-02 |
| E414 : E160c | 0,747 | -6,59E-05 | -4,66E-04 | 3,34E-04 | -1,41E-02 | -9,99E-02 | 7,17E-02 |
| E338 : E160c | 0,759 | 7,28E-03 | -3,92E-02 | 5,38E-02 | 1,58E-01 | -8,54E-01 | 1,17E+00 |
| E150d : E160c | 0,767 | -6,61E-04 | -5,03E-03 | 3,71E-03 | -1,53E-01 | -1,17E+00 | 8,60E-01 |
| E331 : E338 | 0,774 | 1,02E-05 | -5,91E-05 | 7,95E-05 | 1,21E-01 | -7,04E-01 | 9,46E-01 |
| E150d : E440 | 0,801 | -9,20E-07 | -8,07E-06 | 6,23E-06 | -1,32E-01 | -1,16E+00 | 8,93E-01 |
| E330 : E163 | 0,801 | -1,74E-06 | -1,53E-05 | 1,18E-05 | -6,75E-03 | -5,94E-02 | 4,59E-02 |
| E296 : E440 | 0,826 | 6,50E-07 | -5,14E-06 | 6,44E-06 | 1,09E-02 | -8,62E-02 | 1,08E-01 |
| E331 : E903 | 0,844 | 2,12E-05 | -1,90E-04 | 2,33E-04 | 1,59E-02 | -1,43E-01 | 1,75E-01 |
| E950 : E412 | 0,848 | 2,30E-06 | -2,11E-05 | 2,57E-05 | 7,38E-03 | -6,79E-02 | 8,26E-02 |
| E150d : E950 | 0,853 | -1,15E-05 | -1,33E-04 | 1,10E-04 | -7,59E-02 | -8,78E-01 | 7,26E-01 |
| E330 : E950 | 0,854 | 6,60E-07 | -6,35E-06 | 7,67E-06 | 4,89E-03 | -4,71E-02 | 5,68E-02 |
| E338 : E950 | 0,859 | 1,18E-04 | -1,19E-03 | 1,42E-03 | 7,30E-02 | -7,35E-01 | 8,81E-01 |
| E338 : E150d | 0,868 | 1,05E-07 | -1,13E-06 | 1,34E-06 | 2,21E-03 | -2,37E-02 | 2,82E-02 |
| E330 : E440 | 0,871 | -2,60E-08 | -3,40E-07 | 2,88E-07 | -4,19E-03 | -5,47E-02 | 4,64E-02 |
| E160c | 0,880 | 1,36E-02 | -1,64E-01 | 1,91E-01 | -2,57E-02 | -1,01E-01 | 4,95E-02 |
| E338 : E414 | 0,894 | -9,08E-06 | -1,42E-04 | 1,24E-04 | -1,77E-01 | -2,77E+00 | 2,42E+00 |
| E950 : E440 | 0,897 | 9,62E-07 | -1,36E-05 | 1,55E-05 | 4,05E-03 | -5,73E-02 | 6,54E-02 |
| E150d : E414 | 0,900 | -7,78E-07 | -1,29E-05 | 1,13E-05 | -1,61E-01 | -2,67E+00 | 2,35E+00 |
| E160c : E163 | 0,912 | 1,00E-03 | -1,68E-02 | 1,88E-02 | 3,59E-03 | -6,00E-02 | 6,72E-02 |
| E338 : E412 | 0,912 | -6,77E-06 | -1,27E-04 | 1,13E-04 | -6,94E-02 | -1,30E+00 | 1,16E+00 |
| E330 : E955 | 0,922 | -2,92E-07 | -6,11E-06 | 5,53E-06 | -2,22E-03 | -4,64E-02 | 4,20E-02 |
| E951 : E163 | 0,924 | 2,15E-05 | -4,18E-04 | 4,61E-04 | 4,73E-03 | -9,21E-02 | 1,02E-01 |
| E338 | 0,938 | 1,96E-03 | -4,75E-02 | 5,14E-02 | 2,14E-01 | -1,41E+00 | 1,84E+00 |
| E330 : E150d | 0,939 | 1,21E-07 | -2,99E-06 | 3,23E-06 | 3,04E-02 | -7,53E-01 | 8,13E-01 |
| E331 : E412 | 0,947 | -4,38E-08 | -1,35E-06 | 1,26E-06 | -2,70E-03 | -8,31E-02 | 7,77E-02 |
| E150d : E955 | 0,955 | 8,81E-06 | -3,01E-04 | 3,18E-04 | 5,96E-02 | -2,03E+00 | 2,15E+00 |
| E150d : E412 | 0,959 | 2,95E-07 | -1,09E-05 | 1,15E-05 | 3,23E-02 | -1,19E+00 | 1,26E+00 |
| E330 : E338 | 0,963 | 7,88E-07 | -3,24E-05 | 3,40E-05 | 1,86E-02 | -7,67E-01 | 8,04E-01 |
| E950 : E296 | 0,969 | 1,76E-06 | -8,63E-05 | 8,99E-05 | 1,36E-03 | -6,67E-02 | 6,95E-02 |
| E955 : E414 | 0,970 | 1,54E-07 | -7,80E-06 | 8,11E-06 | 9,64E-04 | -4,88E-02 | 5,07E-02 |
| E414 : E296 | 0,990 | 1,52E-08 | -2,42E-06 | 2,45E-06 | 3,70E-04 | -5,88E-02 | 5,96E-02 |
| E150d | 0,992 | 2,50E-05 | -4,58E-03 | 4,63E-03 | -1,11E-01 | -1,72E+00 | 1,50E+00 |
| E338 : E955 | 0,992 | -1,62E-05 | -3,34E-03 | 3,31E-03 | -1,03E-02 | -2,12E+00 | 2,10E+00 |
| E330 : E414 | 0,996 | -1,46E-09 | -5,26E-07 | 5,23E-07 | -3,41E-04 | -1,23E-01 | 1,22E-01 |

For a given model, the individual food additives (intake in mg/d, continuous variable) as well as the product of the two corresponding variables (i.e. interaction term) were simultaneously introduced into Cox models.

Multivariable Cox proportional hazard models were adjusted for age (time-scale), sex, Body Mass Index (BMI, continuous, kg/m²), physical activity (categorical International Physical Activity Questionnaire (IPAQ) variable: high, moderate, low), smoking status (never smoked, former smoker, current smokers), number of smoked cigarettes in pack-years (continuous), educational level (did not complete secondary education / up to two years of university studies / bachelor degree or higher), socio-professional categories (farmer, craftsman/shopkeeper/entrepreneur, managerial staff/intellectual profession, intermediate profession, employee, manual worker, retired, unemployed, student, and other without professional activity), monthly income per household unit (<1200 €/month; 1200–1800 €/month; 1800–2700 €/month; >2700 €/month), family history of type 2 diabetes (yes/no), number of dietary records (continuous), intakes of energy without alcohol (continuous, kcal/d), saturated fatty acids (continuous, g/d), sodium (continuous, mg/d), dietary fibre (continuous, g/d), alcohol (continuous, g/d), and added sugars (continuous, g/d). Each food additive has been standardized to have a mean of 0 and a standard deviation of 1, enhancing interpretability.

## Table L. Mediation analyses

### A. Associations between the food groups most correlated with mixtures 2 and 5 and type 2 diabetes incidence, NutriNet-Santé cohort, 2009-2023 (n=108,643 participants; 1,131 incident cases)

|  | HR for an increment  of 100 g or ml / d | 95%CI | P-value |
| --- | --- | --- | --- |
| Fats and sauces | 1.06 | (1.00, 1.13) | 0.04 |
| Dairy desserts | 1.04 | (0.97, 1.11) | 0.33 |
| Broths | 1.01 | (0.95, 1.07) | 0.86 |
| Artificially sweetened beverages | 1.11 | (1.07, 1.16) | <0.001 |
| Sugary drinks | 1.12 | (1.05, 1.20) | 0.001 |

^a^“Broth” refers to a food item called "bouillon" in French, which is a clarified soup or seasoned liquid typically made by simmering meat, vegetables, and seasonings in water. It can be consumed as a standalone dish.

### B. Proportion of the associations mediated by mixtures 2 and 5

|  | Mediator | Proportion of the association mediated by the mixture | P-value |
| --- | --- | --- | --- |
| Association between Fats and sauces and T2D | Mixture 2 | 18% | 0.09 |
| Association between Artificially sweetened beverages and T2D | Mixture 5 | 52% | 0.03 |
| Association between Sugary drinks and T2D | Mixture 5 | 42% | <0.001 |

TABLE 3

Abbreviations: HR: Hazard ratio; CI: Confidence Interval

Mixtures of food additives were derived from non-negative matrix factorisation (NMF, Supplemental eMethod4).

Mediation analyses were performed using the CMAVERSE R package.

Multivariable Cox proportional hazard models were adjusted for age (time-scale), sex, Body Mass Index (BMI, continuous, kg/m²), physical activity (categorical International Physical Activity Questionnaire (IPAQ) variable: high, moderate, low), smoking status (never smoked, former smoker, current smokers), number of smoked cigarettes in pack-years (continuous), educational level (did not complete secondary education / up to two years of university studies / bachelor degree or higher), family history of type 2 diabetes (yes/no), number of dietary records (continuous), socio-professional categories (farmer, craftsman/shopkeeper/entrepreneur, managerial staff/intellectual profession, intermediate profession, employee, manual worker, retired, unemployed, student, and other without professional activity), monthly household income per consumption unit (<1200 €/month; 1200–1800 €/month; 1800–2700 €/month; >2700 €/month), intakes of energy without alcohol (continuous, kcal/d), saturated fatty acids (continuous, g/d), sodium (continuous, mg/d), dietary fibre (continuous, g/d), alcohol (continuous, g/d), added sugars (continuous, g/d).

# eReferences

1 Moullec NL, Deheeger M, Preziosi P, *et al.* Validation du manuel-photos utilisé pour l’enquête alimentaire de l’étude SU.VI.MAX. *Nutr Clin Metab* 1996; **31**: 158–64.

2 Arnault N, Caillot L, Castetbon K, et al. Table de composition des aliments, étude NutriNet-Santé. [Food composition table, NutriNet-Santé study] (in French). 2013.

3 Touvier M, Méjean C, Kesse-Guyot E, *et al.* Comparison between web-based and paper versions of a self-administered anthropometric questionnaire. *Eur J Epidemiol* 2010; **25**: 287–96.

4 Lassale C, Castetbon K, Laporte F, *et al.* Validation of a Web-based, self-administered, non-consecutive-day dietary record tool against urinary biomarkers. *Br J Nutr* 2015; **113**: 953–62.

5 Lassale C, Castetbon K, Laporte F, *et al.* Correlations between Fruit, Vegetables, Fish, Vitamins, and Fatty Acids Estimated by Web-Based Nonconsecutive Dietary Records and Respective Biomarkers of Nutritional Status. *J Acad Nutr Diet* 2016; **116**: 427-438.e5.

6 Black AE. Critical evaluation of energy intake using the Goldberg cut-off for energy intake:basal metabolic rate. A practical guide to its calculation, use and limitations. *IntJObesRelat Metab Disord* 2000; **24**: 1119–30.

7 Black AE. The sensitivity and specificity of the Goldberg cut-off for EI:BMR for identifying diet reports of poor validity. *Eur J Clin Nutr* 2000; **54**: 395–404.

8 Schofield WN. Predicting basal metabolic rate, new standards and review of previous work. *HumNutr Clin Nutr* 1985; **39 Suppl 1**: 5–41.

9 Anses. Etude Individuelle Nationale des Consommations Alimentaires 3 (INCA 3). 2017.

10 GNPD - Global New Products Database, Monitoring New Product Trends and Innovations. https://www.gnpd.com/sinatra/anonymous_frontpage (accessed Feb 25, 2023).

11 GFSA. Codex General Standard for Food Additives (GSFA, Codex STAN 192-1995). Codex Alimentarius Commission 2018. http://www.fao.org/fao-who-codexalimentarius/sh proxy/en/?lnk=1&url=https%253A%252F%252Fworkspace.fao.org%252Fsites%252Fcodex%252FStandards %252FCODEX%2BSTAN%2B192-1995%252FCXS_192e.pdf. (accessed July 21, 2024).

12 Chazelas E, Druesne-Pecollo N, Esseddik Y, *et al.* Exposure to food additive mixtures in 106,000 French adults from the NutriNet-Santé cohort. *Sci Rep* 2021; **11**: 19680.

13 Paatero P, Tapper U. Positive matrix factorization: A non-negative factor model with optimal utilization of error estimates of data values. *Environmetrics* 1994; **5**: 111–26.

14 Lee DD, Seung HS. Learning the parts of objects by non-negative matrix factorization. *Nature* 1999; **401**: 788–91.

15 Zetlaoui M, Feinberg M, Verger P, Clémençon S. Extraction of food consumption systems by nonnegative matrix factorization (NMF) for the assessment of food choices. *Biometrics* 2011; **67**: 1647–58.

16 Hoyer PO. Non-negative matrix factorization with sparseness constraints. *J Mach Learn Res* 2004; **5**.

17 Lee D, Seung H. Algorithms for Non-negative Matrix Factorization. *Adv Neural Inf Process Syst* 2001; **13**.

18 Brunet J-P, Tamayo P, Golub TR, Mesirov JP. Metagenes and molecular pattern discovery using matrix factorization. *Proc Natl Acad Sci* 2004; **101**: 4164–9.

19 Pascual-Montano A, Carazo JM, Kochi K, Lehmann D, Pascual-Marqui RD. Nonsmooth nonnegative matrix factorization (nsNMF). *IEEE Trans Pattern Anal Mach Intell* 2006; **28**: 403–15.

20 Harrell FE, Dupont C. Harrell miscellaneous. *R Package Version* 2018; : 4–0.

21 Cai Y, Gu H, Kenney T. Rank selection for non-negative matrix factorization. *Stat Med* 2023; **42**: 5676–93.
