# Supplementary material for: Food additive mixtures and type 2 diabetes incidence: Results from the NutriNet-Santé prospective cohort
Source: PLoS Med. 2025 Apr 8;22(4):e1004570. doi: 10.1371/journal.pmed.1004570 (PMC11977966; doi:10.1371/journal.pmed.1004570)
Supplement: Attachment — Submitted filename: garanderie.pdf [file pmed.1004570.s004.pdf]

This paper examines the association between mixtures of food additives and subsequent type 2 diabetes in a large French cohort.

I was asked for a methodological report and I interpret that to include all aspects of the design and conduct of the study.

# Points of detail

**Page 3** I think I would mention the average follow–up time here. It is important for casual readers to see whether this is a short– or long–term study.

**Page 7** I am sure the authors are aware that the NOVA classification has been criticised over the years. It only seems to get a brief mention later on page 10 and is not used in the models (unless I missed it) so probably not a serious issue.

**Page 8** Typo incline should be inclined. Having said that liable or likely would seem more natural to me.

**Page 10** It is always tricky to translate between educational systems not helped here by the different meaning of 'high school' in different Anglophone countries. I do not think we Anglophones use 'degree' consistently either. Are these categories: did not complete secondary education (which I believe would mean they did not achieve the *baccalauréat*), completed but did not complete university, completed university?

**Page 10** The phrase about the restricted cubic spline is repeated. Note that it was R 4.3.3 which was used, RStudio is the IDE which sits above it.

**Page 10** For some of the descriptives I wonder whether medians and other selected quantiles would be more useful? They are used elsewhere (for instance in Table 1).

**Page 11 and elsewhere** I would understand broth to mean the same as stock (a flavoured liquid used to make other recipes) although there are some soups called broths (most famously Scotch Broth). Is soup meant here?

**Page 16** Typo singles should be single.

**Page 23** I am constantly being reminded on packages that adults need 2000 kcal/day (in the UK). I am therefore impressed that so few of this cohort exceed that. Does that throw any light on the representativeness of the cohort or its generalisability internationally?

**Supplement page 6** Which analyses were adjusted for multiplicity? I would not have considered that the mixtures were examples of multiplicity since presumably the theory and underlying mechanisms were different for each. I would not have adjusted each sensitivity analysis for the others either for rather similar reasons. The authors have not gone on a fishing expedition. Perneger (1998); Cook and Farewell (1996); Schulz and Graimes (2005a,b) have some thoughts on the matter.

# Points of more substance

In the models the authors have included as covariates a number of food groups. If those food groups contain one or more of the mixtures is there a danger that this is adjusting out the effect of the additive mixture (because it is, in part, in the model twice)? The authors do allude to this issue in their discussion (page 13) but I wonder whether there is anything to be gained by other approaches.

# Summary

No major issues but some points for clarification.

Michael Dewey

# References

R J Cook and V T Farewell. Multiplicity considerations in the design and analysis of clinical trials. *Journal of the Royal Statistical Society — Series A*, 159:93–110, 1996.

T V Perneger. What's wrong with Bonferroni adjustments. *British Medical Journal*, 316:1236–1238, 1998.

K F Schulz and D A Graimes. Multiplicity in randomised trials I: endpoints and treatments. *Lancet*, 365:1591–1595, 2005a.

K F Schulz and D A Graimes. Multiplicity in randomised trials II: subgroups and interim analyses. *Lancet*, 365:1657–1661, 2005b.
