# Supplementary material for: Food additive mixtures and type 2 diabetes incidence: Results from the NutriNet-Santé prospective cohort
Source: PLoS Med. 2025 Apr 8;22(4):e1004570. doi: 10.1371/journal.pmed.1004570 (PMC11977966; doi:10.1371/journal.pmed.1004570)
Supplement: Attachment — Submitted filename: Response to reviewer.docx [file pmed.1004570.s006.docx]

Review "Food additive mixtures and risk of type 2 diabetes: results from the NutriNet-Santé cohort" (PMEDICINE-D-24-03030R1) – Point-by-point responses to Editors and Reviewers’ comments

-----------------------------------------------------------

Comments from the academic editor:

The authors conducted an observational study relating 'mixtures' of food additives to incidence of type 2 diabetes. The major and minor comments are supplied hereafter.

🡪 We thank the Academic Editor for his/her thorough analysis and insightful comments. All of them have been addressed below.

Major comments:

0. Some of the individual food additives were evaluated previously, as cited #5 (Lancet Diabetes Endocrinol, 2024) and were shown to be associated with type 2 diabetes incidence in the same population. Because of the associations, the novelty of this current study is weak. Also, two analytic studies from the same cohort could question whether single chemicals or a combination played a role in showing the positive association. The authors discussed it but did not address the question.

The previous study reported in Lancet D&E 2024 had the weakness of mutual confounding between food additives (emulsifiers), and then this study demonstrated it. It would be an important notion, but it remained unclear if the positive association was due to a single factor or a combination. Without such an in-depth investigation, this study seems weak.

The authors argued the "cocktail effect", but a specific analysis to test the presence is absent, and the method is too weak to demonstrate or argue the effect. The authors cannot rule out the possibility that a single agent drove the positive association. Even if the "cocktail effect" looked significant, measurement errors and confounding would remain concerning in the multivariable setting.

The authors should design their analyses to test the effect of multiple food additives above and beyond the individual ones. Of note, several food additives in the statistically identified "mixture" were confirmed to be associated with type 2 diabetes, so the confounded interpretations should be avoided. The authors seem aware of the research question (Page 14 in the Discussion) but not aware of proper methods, but any approaches need to be conducted with or without limitations/assumptions. At least, the authors should cite the Lancet D&E paper and re-justify this study to test the hypothesis of the interaction.

The "Cocktail" effect is not a technical term, and the authors should avoid it. What the authors examined must align with a hypothesis about an interaction. The authors should document their interest using such a known term.

🡪 We fully agree that this is a key question and thank the Academic Editor for raising this point.

In the previous papers that we published on food additives and T2D risk in NutriNet-Santé (1–3), the objective was to investigate potential effects of one specific food additive (or food additive category), independently from the intake of other additives. Therefore, we adjusted the models (main or sensitivity) for the proportion of ultra-processed foods in the diet (as an overall indicator of multiple additive exposure), and/or for other additives than the one studied (e.g. intake of all emulsifiers except the one studied in the model). These analyses suggest potential effects of these additives per se (so far some artificial sweeteners, emulsifiers, nitrites), consistent with experimental/mechanistic data that initially conducted us to set up these epidemiological investigations (4–6). In these experimental *in vivo / in vitro* studies, the effect of each specific additive is isolated and tested, thus, results cannot be attributed to a potential mixture effect.

The present paper is very complementary. Our hypothesis is that beyond potential effects of individual additives (and not instead of), there may be effects of the mixtures themselves, resulting from interactions / synergies between food additives. In order to investigate this specific aspect, we have now conducted two additional analyses for mixtures 2 and 5 (for which we observed associations with T2D risk):

1) As proposed by Academic Editor Point 4, in order to investigate whether the associations between mixtures and T2D risk were in fact entirely driven by one or some specific additives (and not the mixture itself), we adjusted the main Cox model for each food additive characteristic of the mixture, using the residual method. To do so, we performed residual analyses: linear regression models of mixture 2 (resp. mixture 5) on each individual food additive characteristic of the mixture were used to extract residuals. These residuals were then included into the main Cox models to assess the association between mixtures and T2D risk, independent from the variability due to each main additive composing the mixture. Despite slight attenuations, the associations between mixtures 2 and 5 and T2D risk remained similar, suggesting that the associations were not strongly driven by a unique additive alone. This has been incorporated into the Methods section (page 11), the Results section (page 14) and in eTable10.
2) Interactions between the key food additives contributing to mixture 2 (resp. mixture 5) on T2D risk were formally tested (two by two interactions). Several interactions were statistically significant, supporting the hypothesis of a potential “mixture effect”. These analyses have been added in the Methods (page 11), the Results section (page 14) and in eTable11.

Overall, these findings support our hypothesis of a “mixture effect” that goes beyond the potential impact of individual food additives. Further experimental studies are needed to gain a deeper understanding of potential mixture effects. An increasing number of toxicological studies support the relevance of these effects of mixtures of food constituents or contaminants (7,8). Several in vitro and in vivo experiments are being conducted by teams of toxicologists, partners of the present ERC ADDITIVES project. A first paper, that has just been accepted for publication in *Food and Chemical Toxicology,* aligns with our findings: it tested *in vitro* food additives for cytotoxic and genotoxic effects, on the one hand one by one, and on the other hand, combined in mixtures, and observed mixtures effects beyond those of individual substances (9). We have added information in the discussion pages 18 to elaborate on this important aspect.

Last, in order to comply with the Editor’s request, we have replaced the term “cocktail” throughout the manuscript by “interaction/synergistic effects”.

1. The authors documented the limitation of residual confounding, but the effort to reduce it and the highlight seem insufficient. The authors collected the majority of the information through the Internet. Self-reports partly affected by health consciousness must cause differential measurement errors, such as those of body weight and height, as is often the case, particularly among women. Such uncharacterized errors could happen to many covariates, cause insufficient adjustment for those and cause further residual confounding.

When the authors described the study limitations, they argued, "Despite extensive adjustments for confounding variables, including dietary, lifestyle, anthropometric, and socio-demographic factors, ..."

This statement indicated that the authors had identified many covariates to adjust for in their analysis. However, socioeconomic status (SES) was not adjusted for well. It likely contributed to dietary behaviours, health consciousness, health-seeking behaviours, and others that influence health outcomes through numerous mechanisms. Despite the well-known confounding roles of SES, the authors included only a single three-level covariate of education history. Just three levels were unlikely to capture the SES. The authors must be aware of the diversity of the study population, including race/ethnic, regional, cultural, and economic diversity across France. The authors did not account for any of those, except for educational attainment. The authors should make a greater effort.

The authors' group may not have considered it in previous studies from the same cohort, but that would not matter. The authors should adjust for a reasonable amount of SES variables, such as household or individual incomes, occupations, race/ethnic status, and religion. (Otherwise, collider bias happens in the current analysis, causing unmeasured confounding.).

Health consciousness is also a potential confounder, as the authors noted. The authors should adjust for participation in clinical screening, access to dental care, and others.

Those covariates would matter in this study because the authors have identified weak positive associations. Weak positive confounders could elevate the likelihood of detecting false-positive associations.

🡪 The main model is now adjusted for detailed socio-professional categories and monthly household income per consumption unit (in addition to educational level already included). A new sensitivity analysis model (Model 6, eTable7 in the Supplementary material) was adjusted for an indicator of health-seeking behaviours (not included in the main model because of a higher proportion of missing values), i.e. the total number of screening tests or exams in the lifecourse (including CT colonography, colonoscopy or sigmoidoscopy, screening test for blood in stools, PAP smear, skin cancer screening exam, mammography, medical breast palpation, PSA blood testing and digital rectal examination). The results remained similar after these new adjustments. This has been added in the Methods page 10, and results have been modified in the text, tables and supplemental throughout the documents.

Race/ethnicity and religion are not available in the cohort due to a very restrictive ethical/legal regulation policy regarding the collection of these data in French epidemiological studies (specific authorizations needed). We have added this point in the limitation section of the Discussion page 19.

Regarding the use of self-reported data via the Internet: NutriNet-Santé was the first web-cohort of this scale internationally when it was launched in 2009. Thus, numerous methodological studies in e-epidemiology were conducted and published, to challenge the reliability of the information collected online. These included comparisons with traditional data collection methods (e.g., paper-and-pencil or interviews by trained dietitians) and validation studies against gold standards, such as blood and urine biomarkers of nutritional intakes and investigator-measured height and weight (10–15). High consistency was observed between online tool and standard methods, supporting the reliability of the data collected, which has been used in the framework of many previous epidemiological papers, e.g. (16–18). The methodological studies even highlighted the advantage of online web-questionnaires in increasing the quality of collected data and reducing input error and outliers thanks to integrated field controls and automated conditional skips. Several studies also showed that web-based data collection may even increase accuracy by lowering social desirability bias associated with face-to-face interviews (19–21). Indeed, people being less exposed to direct judgment by an interviewer tend to more and better declare sensitive information such as ‘at risk’ practices (consumption of “unhealthy” fatty and sweet foods, alcohol, tobacco or drug use) but also their social status (unemployment, income). It is merely impossible to guaranty a perfect measurement of exposure and covariates in any population-based epidemiological study in real-life setting. However, in etiological studies, the ability to rank participants with contrasted exposures between themselves is more important than reaching perfectly accurate absolute values. Besides, misclassification in exposure or covariates may have induced a non-differential measurement error (identically in future cases and non-cases), but although an overestimation cannot be excluded, it most probably led to an underestimation of the observed associations. The Discussion section has been extended on this point page 19.

2.

The authors investigated a linear combination of multiple food additives. However, this does not mean that the authors examined a synergistic or cocktail effect of the multiple food additives. Possibly, only a single food additive within a mixture was causal, whereas other additives had no effect. Taking the possibility, the authors should limit and tone down their conclusion as if there were a cocktail effect. Then, they should argue the need for experimental studies to identify whether only a single causal agent may play a role predominantly or a synergism may happen.

🡪 Please see our response above, to Academic Editor, Point 0.

3.

The authors identified type 2 diabetes cases with both subjective (self-reports) and objective information. The approach is great but could miss undiagnosed type 2 diabetes, which could be present to an unignorable degree. Individuals with unrecognized diabetes cannot be identified by self-reports or the national registry. The authors should discuss it.

🡪 Indeed, we agree with this point. Despite the multi-source case ascertainment strategy (which combined self-reported disease and medication use, linkage to a national health insurance database, and fasting blood glucose measurements in a subsample), under-detection of certain undiagnosed T2D cases could not be entirely ruled out. In France, the prevalence of undiagnosed diabetes cases is estimated around 1.7% (IC95% 1.1 to 2.4), higher in men (2.7% IC95% 1.4 to 4.0) than in women (0.9% IC95% 0.3 to 1.4) (Esteban nationally representative study 2016, among people aged 18 to 74) (22). However, such limitation would primarily result in reduced statistical power and non-differential misclassification. Consequently, it likely led to an underestimation of the observed associations and in turn, it is not likely to explain the statistically significant associations observed. This point has been added to the Discussion section page 20.

4.

For each of the "mixture" #2 and #5, the authors should fit a regression model including individual food additives that contribute to the mixture and the mixture itself. If the positive association of the mixture with TYPE 2 DIABETES remains, that should be considered as evidence that the combination matters beyond individual effects.

Similarly, the authors should demonstrate whether or not the positive association was driven by single factor or multiple factors.

Basically, the authors should not give up differentiating between the potential of individual effects and the potential of the effect of the combination or interaction.

These analyses are essential for this manuscript, in addition to the maximal effort to adjust for socioeconomic status and health-seeking behaviours. For the latter confounding, the authors should identify if the participants joined any screening activities, and I hope those are available in their registration data / linked medical records.

🡪 Thank you for these insights that led us to perform additional analyses and strengthen the manuscript. Please see our responses above, to Academic Editor, Points 0 and 1.

5.

In Discussion, the authors should discuss whether the previous studies on emulsifiers and artificial sweeteners should be interpreted. It seems that mutual confounding between food additives substantially matter. The authors should explicitly state that previous findings should be interpreted more than published, with regards to the issue of mutual confounding.

🡪 Please see our response above, to Academic Editor, Point 0.

**Minor comments:**

Risk and incidence should be distinguished.

🡪 As the proportional hazards assumption is met, the hazard ratio serves as an estimate of the relative risk. For this reason, we consistently use the term "risk" throughout the manuscript.

The aggregates of the food additives are unclear in terms of their clinical relevance. The analyses conducted for the current eTable 4 should be elaborated more to identify food sources and dietary patterns. SES may better be carefully considered, too.

🡪 The studied mixtures are clinically relevant because they represent the ones to which consumers are most frequently exposed daily. Indeed, the approach was to derive NMF components based on the observation of real-life consumption data in this large-scale population study. We have added this precision in the Discussion page 18. Of note: we are currently reproducing the NMF analysis in the nationally representative study Esteban and preliminary results show similarity in the identification of main mixtures to which French citizens are exposed.

As requested, we expanded the descriptive analyses presented in eTable4 (now eTable5) for mixtures 2 and 5: the detailed consumption of food groups is now provided by quintiles of NMF score for mixture 2, and respectively for mixture 5, in the newly added eTable6 and is mentioned in the manuscript Page 9 (Methods) et 12 (Results).

As explained in our response to Academic Editor Point 1, all models are now adjusted for detailed socio-professional categories and monthly household income per consumption unit (in addition to educational level already included).

On page 6 and the other pages, the authors should not use "validated" to describe the validity of their measurements. No variable must have been measured with perfection. The authors should document how valid each measure was as much as possible. Given the aim of this work, the authors must be mindful of whether or not each measured variable could identify a between-individual difference rather than its absolute levels of exposure. Thus, the authors should document the quantitative abilities of the exposures and covariates (such as BMI) to rank individuals.

As mentioned above, the measurement error of BMI and behavioural factors were more concerning than usual because the authors recruited individuals over the Internet and collected self-reports of many variables.

🡪 Please see our response to Academic Editor, Point 1.

Page 8:

The authors documented specifications in their dimension-reduction technique. Those selections are subject to sensitivity analyses.

🡪 Indeed, we chose to use the algorithm introduced by Lee and Sung (23) for non-negative matrix factorisation because it aligns with our goal of identifying the main food additive mixtures to which participants are exposed, it guarantees non-negativity (e.g., additive intakes cannot be negative), and it is particularly effective at handling the noise commonly found in dietary data (23). For these reasons, it is one of the most widely used decomposition algorithms in the literature (24) and has been applied in many large-scale food consumption surveys (25,26). The number of ranks (i.e. the number of NMF components to retain) was determined according to the method proposed by Brunet et al. (27), using the smallest number for which the cophenetic coefficient starts decreasing, as visualized on the consensus map (now provided in eTable3a). We have added this precision in the manuscript (Page 9). We conducted sensitivity analyses by testing different NMF algorithms, including Brunet (27) and non-smooth non-negative matrix factorisation (nsNMF) (28). The mixtures obtained with these methods have been added in eTables3b and 3c. These variations in methodological choices led to identify similar mixtures, supporting the robustness of the results.

-----------------------------------------------------------

Comments from the reviewers:

Reviewer #1: See attachment

Points of detail

1. Page 3 I think I would mention the average follow–up time here. It is important for casual readers to see whether this is a short– or long– term study.

🡪 We thank Reviewer #1 for his valuable suggestions to improve the manuscript. The average follow-up time has been added accordingly in the abstract page 3.

2. Page 7 I am sure the authors are aware that the NOVA classification has been criticized over the years. It only seems to get a brief mention later on page 10 and is not used in the models (unless I missed it) so probably not a serious issue.

🡪 Indeed, NOVA has only been used in this paper page 11 to provide an estimate of the proportion of daily energy intake by ultra-processed foods, as a descriptor of the study population, since this indicator has been used in many countries, facilitating comparisons (29).

3. Page 8 Typo incline should be inclined. Having said that liable or likely would seem more natural to me.

🡪 We have replaced by “likely” page 8.

4. Page 10 It is always tricky to translate between educational systems not helped here by the different meaning of ‘high school’ in different Anglophone countries. I do not think we Anglophones use ‘degree’ consistently either. Are these categories: did not complete secondary education (which I believe would mean they did not achieve the baccalaureat), completed but did not complete university, completed university?

🡪 The educational level category labeling has been modified as follows: did not complete secondary education / up to two years of university studies / bachelor degree or higher) throughout the manuscript.

5. Page 10 The phrase about the restricted cubic spline is repeated. Note that it was R 4.3.3 which was used, RStudio is the IDE which sits above it.

🡪 These two points have been corrected page 11, sorry for the typo.

6. Page 10 For some of the descriptives I wonder whether medians and other selected quantiles would be more useful? They are used elsewhere (for instance in Table 1).

🡪 Medians (25^th^ – 75^th^ percentiles) are now provided.

7. Page 11 and elsewhere I would understand broth to mean the same as stock (a flavoured liquid used to make other recipes) although there are some soups called broths (most famously Scotch Broth). Is soup meant here?

🡪 Indeed, “broth” refers to a food item called "bouillon" in French, which is a clarified soup or seasoned liquid typically made by simmering meat, vegetables, and seasonings in water. It can be consumed as a standalone dish. We have added this clarification in a footnote to eTable5.

8. Page 16 Typo singles should be single.

🡪 This has been corrected page 15.

9. Page 23 I am constantly being reminded on packages that adults need 2000 kcal/day (in the UK). I am therefore impressed that so few of this cohort exceed that. Does that throw any light on the representativeness of the cohort or its generalisability internationally?

🡪 The standard “2000 kcal/day” indicated by manufacturers on food packaging does not account for individual variations in energy needs, such as those based on physical activity levels, anthropometry or sex. Mean daily energy intake without alcohol in the French general population has been estimated at 2323 kcal/day for men and 1751 kcal/day for women in the INCA3 nationally representative survey (30). Given that our study population is predominantly female, the mean energy intake without alcohol observed in our cohort (1840 kcal/day) is fully consistent with national estimates. We have added the reference to this national estimate in the Discussion page 20.

10. Supplement page 6 Which analyses were adjusted for multiplicity? I would not have considered that the mixtures were examples of multiplicity since presumably the theory and underlying mechanisms were different for each. I would not have adjusted each sensitivity analysis for the others either for rather similar reasons. The authors have not gone on a fishing expedition. Perneger (1998); Cook and Farewell (1996); Schulz and Graimes (2005a,b) have some thoughts on the matter.

🡪 We agree that correction for multiple testing is not necessary with only 5 exposures tested (the 5 mixtures). Thus, the point on FDR has been removed.

11. In the models the authors have included as covariates a number of food groups. If those food groups contain one or more of the mixtures is there a danger that this is adjusting out the effect of the additive mixture (because it is, in part, in the model twice)? The authors do allude to this issue in their discussion (page 13) but I wonder whether there is anything to be gained by other approaches.

🡪 Food additives are ubiquitous in industrial food products and most of them are not specific to a defined food group but rather used in multiple ones. The identified mixtures of food additives result from their co-occurrence within foods but also within the overall diet, based on combinations of foods and beverages usually consumed together. As illustrated in eTable5, additive mixtures are correlated with some food groups but not at all colinear, limiting the risk for over-adjustment. However, in order to comply with this comment and with the request of Reviewer #2 Point 1, the main model now only includes adjustment for nutrient intakes but not food groups, and another model (sensitivity analysis, eTable7, Model 5) adjusts for food group intakes but not nutrients. Both models provide similar results. The Methods (page 10), Figure 1 and Supplemental tables have been modified accordingly.

Summary

No major issues but some points for clarification. Michael Dewey

References

R J Cook and V T Farewell. Multiplicity considerations in the design and analysis of clinical trials. Journal of the Royal Statistical Society — Series A, 159:93–110, 1996.

T V Perneger. What’s wrong with Bonferroni adjustments. British Medical Journal, 316:1236–1238, 1998.

K F Schulz and D A Graimes. Multiplicity in randomised trials I: endpoints and treatments. Lancet, 365:1591–1595, 2005a.

K F Schulz and D A Graimes. Multiplicity in randomised trials II: subgroups and interim analyses. Lancet, 365:1657–1661, 2005b.

Michael Dewey

Reviewer #2: The paper by de la Garanderie et al. Reports a very fancy prospective cohort analysis on the relationship between the consumption of 5 different food additive mixtures and type 2 diabetes incidence, conducted in the large NutriNetSanté cohort in France.

Investigators first characterized presence/absence and doses of several food additives by leveraging food records completed by participants, ingredient lists coupled with different databases on food additives. This method was previously described and published in Sci Rep.

They then used a data-driven approach (NMF) to identify 5 food additive mixtures. These mixtures represent "clusters" of food additive commonly consumed together due to concomitant presence in the same food for instance.

They then assessed using cox models the relationship between each mixture and TYPE 2 DIABETES incidence. Two mixtures were found to be positively associated with TYPE 2 DIABETES risk. The first was mostly composed of emulsifiers and the second was composed of food additives commonly present in SSB and ASB.

Analyses are comprehensive and the paper is well written.

I think this paper can become an important contribution to the public health discussion on the place of UPF in the diet.

🡪 We thank Reviewer #2 for this overall positive comment.

1. My main concern is related to the separation between the effects of the food additive mixtures per se from the one of the food matrix per se, or even the diet pattern. As mentioned by the authors, the first mixture included additives present in a variety of UPF, including broth dairy desserts, fats and sauces - all foods that have been associated with higher risk of TYPE 2 DIABETES in many cohorts. Likewise, the other group was SSB/ASB driven, which are also important dietary risk factors of TYPE 2 DIABETES (mostly SSB). In an attempt to control for diet quality, authors adjusted for intakes of energy without alcohol (continuous, kcal/d), total saturated fatty acids (continuous, g/d), sodium (continuous, mg/d), dietary fibre (continuous, g/d), alcohol (continuous, g/d), added sugars (continuous, g/d), fruits and vegetables (g/d), dairy products (continuous, ml/day), red and processed meats (continuous, g/d). I first question the concomitant presence of nutrients and foods in the model. Adjusting for saturated fat and dairy products and red meat seems inappropriate because these foods are the main sources of saturated fat. I would first suggest using two distinct models, one nutrient-based and the other food-based.

🡪 In order to comply with this comment, the main model now only includes adjustment for nutrient intakes but not food groups, and another model (sensitivity analysis, eTable7, Model 5) adjusts for food group intakes but not nutrients. Both models provide similar results. The Methods (page 10), Figure 1, Results (page 13), and Supplemental tables have been modified accordingly.

2. Even by doing so, the control for the food matrix will likely be imperfect because the food additive mixtures are found in a limited number of different foods. To address this issue, I would invite the authors to test whether the food additive mixtures mediate the relationship between the foods that are the main sources of these food additive and TYPE 2 DIABETES. Such model would provide key information on the potential contribution of the food additive mixture on the causal pathway between the foods that contain these additive and TYPE 2 DIABETES. These models should also include key nutrients know to drive the relationship between the foods and TYPE 2 DIABETES risk. For instance, relative to SSB/ASB consumption, food mixture #5, and TYPE 2 DIABETES: Is food mixture #5 mediating the relationship between SSB/ASB and TYPE 2 DIABETES risk? If yes, how strong is the effect vs with the one of added sugar? If food mixture #5 is not found to be a mediator of the relationship between SSB/ASB and TYPE 2 DIABETES, what are the implications relative to the paper conclusions? The same should be tested with key foods associated with food additive mixture #1.

🡪 We thank Reviewer #2 for this insightful comment.

An important point to keep in mind is that the identified mixtures of food additives result from their co-occurrence within foods directly, but also largely within the overall diet, based on combinations of foods and beverages usually consumed together. Thus, investigating these additive mixtures in association with T2D risk is different from and complementary with previous studies linking intakes of specific food group with T2D risk.

Following Reviewer #2’s suggestion, we have computed several analyses to test whether the mixtures found associated with T2D risk (i.e. mixtures 2 and 5) contribute to mediate the associations between the food groups most associated with these mixtures and T2D risk:

First, we tested the associations of each food group most correlated with mixture 2 (fats and sauces, broths, dairy desserts) and mixture 5 (artificially sweetened beverages, sugary drinks) with T2D risk, using Cox proportional hazards models. As expected, fats and sauces (HR_100g/d_=1.06 [1.00-1.13], p=0.04), artificially sweetened beverages (HR_100ml/d_=1.11 [1.07-1.16], p<0.001) and sugary drinks (HR_100ml/d_=1.12 [1.05-1.20], p=0.001) were associated with increased T2D risk, while no association was detected for broths or dairy desserts (which are broad food groups with very contrasted nutritional and food additive composition – it was not surprising to detect no association with T2D for these food groups overall).

Then, for the detected associations between food groups and T2D, we performed analyses to calculate the proportion of these associations mediated by the corresponding food additive mixtures, using the CMAVERSE R package(31). The mediation of the association between fats and sauces and T2D by mixture 2 was modest (Proportion of the association mediated=18%, p-value=0.09). Mixture 5 mediated 42% of the association between sugary drinks and type 2 diabetes (p < 0.001) and 52% of the association between artificially sweetened beverages and type 2 diabetes (p = 0.03).

These results have been added in the Methods (page 11), in the Results (page 14), and are displayed in Supplemental eTable12.

3. Also, I would strongly recommend adding analyses stratified by diet quality. The two food additive mixtures associated with TYPE 2 DIABETES are associated with UPF, and UPF consumption correlate with lower diet quality, it appears important to evaluate whether the detrimental relationship between the food additive mixtures and TYPE 2 DIABETES remains valid at any level of diet quality. Such data are key for public health policies.

I acknowledge that my recommendations require significant statistical work, but my concerns echo to key questions in the public health debate on the place of UPF in the diet. Addressing these questions would allow significant advances in the UPF field.

Merci

🡪 We thank Reviewer #2 for this interesting comment. Of note: all models are already adjusted for nutritional quality of the diet, in particular, intakes of energy, sodium, saturated fats, added sugar, dietary fiber and alcohol. Indeed, we fully agree on the fact that here, we wanted to isolate the potential “effect” of food additive mixtures from the well-known effect of nutritional factors. As suggested, we conducted additional sensitivity analyses for mixtures 2 and 5 computing the main model, stratified by the Programme National Nutrition Santé – Guidelines Score 2 (PNNS-G2, ≤ / > the sex-specific median). The PNNS-GS2 is a predefined food-based dietary index measuring compliance with the French dietary guidelines (32). Very similar associations were observed in both PNNS-GS2 strata (although borderline non-significant for mixture 2, likely due to halved statistical power) and no interaction was detected between the PNNS-GS2 score and the additive mixtures (p for interaction = 0.8 for mixture 2 and 0.9 for mixture 5), supporting a potential effect of these mixtures on T2D risk independent from the nutritional quality of the diet. This has been added in the Methods page 10, in eTable9, and in the Results section page 13.

Reviewer #3: The paper is quite interesting, and the authors have conducted a remarkable research. However, I have two minor points regarding the study's limitations:

🡪 We thank Reviewer #3 for this overall positive comment. Please find our responses below.

1) The authors stated: "Next, the generalizability of our findings may be influenced by the cohort's demographic characteristics, such as a higher proportion of women and a more health-conscious population. Therefore, caution is warranted when extrapolating our results to broader populations." As the study aims to evaluate an association, the generalizability is not necessarily compromised by demographic characteristics (as in a prevalence st. If the authors believe there are specific biases affecting the associations, they should clearly identify these issues and explain how they might impact the results.

🡪 We fully agree with this remark: a population study may not be representative of the target population but this does not automatically result in biased estimates of associations between exposures and outcomes. This is why in etiological studies, the diversity of the population is more important than the representativeness itself. In NutriNet-Santé, we aimed at having enough contrast between different categories of dietary exposures to conduct etiological analyses, while accounting for a wide diversity of lifestyle profiles. Participants had in average healthier dietary habits than the French population (reporting higher intakes of fruit, vegetables, and fish, and lower intakes of red meat and added fats) (33). However, daily energy intake as well as proportion of energy by ultra-processed foods were similar in our population study compared to estimates from French nationally representative surveys, supporting the generalizability of our findings (30,34). We have added these elements in the Discussion page 20. Of note: we are currently reproducing the NMF analysis in the nationally representative study Esteban and preliminary results show similarity in the identification of main mixtures to which French citizens are exposed.

2) Regarding the final limitation: "Last, this study did not allow us to specifically investigate mechanistic synergies and/or antagonisms between the food additive chemicals characterizing the mixtures per se.". While the authors suggest experimental studies to address this limitation, it's worth noting that certain epidemiological statistical analyses can account for synergistic effects among variables. It would be beneficial if the authors could: a) Explain why this limitation is relevant to the current study. b) Suggest alternative epidemiological approaches that could provide deeper insights into these associations.

🡪 We agree with Reviewer #2 that this important point deserved to be further investigated and discussed. Please see our response to the Academic Editor, Point 0 for all additional analyses performed and discussion added.

Any attachments provided with reviews can be seen via the following link: <https://www.editorialmanager.com/pmedicine/l.asp?i=952446&l=0GS7IOQK>

--------------------------------------------------------- ---

General editorial requests:

(Note: not all will apply to your paper, but please check each item carefully)

All guidelines below have been checked and the documents have been amended accordingly.

*	We ask every co-author listed on the manuscript to fill in a contributing author statement, making sure to declare all competing interests. If any of the co-authors have not filled in the statement, we will remind them to do so when the paper is revised. If all statements are not completed in a timely fashion this could hold up the re-review process. If new competing interests are declared later in the revision process, this may also hold up the submission. Should there be a problem getting one of your co-authors to fill in a statement we will be in contact. Please do not add or remove authors without first discussing this with the handling editor. You can see our competing interests policy here: <http://journals.plos.org/plosmedicine/s/competing-interests>.

*	Please upload any figures associated with your paper as individual TIF or EPS files with 300dpi resolution at resubmission; please read our figure guidelines for more information on our requirements: <http://journals.plos.org/plosmedicine/s/figures>. While revising your submission, please upload your figure files to the PACE digital diagnostic tool, <https://pacev2.apexcovantage.com/>. PACE helps ensure that figures meet PLOS requirements. To use PACE, you must first register as a user. Then, login and navigate to the UPLOAD tab, where you will find detailed instructions on how to use the tool. If you encounter any issues or have any questions when using PACE, please email us at [PLOSMedicine@plos.org](mailto:PLOSMedicine@plos.org).

*	Please ensure that the paper adheres to the PLOS Data Availability Policy (see <http://journals.plos.org/plosmedicine/s/data-availability>), which requires that all data underlying the study's findings be provided in a repository or as Supporting Information. For data residing with a third party, authors are required to provide instructions with contact information (web or email address) for obtaining the data. Please note that a study author cannot be the contact person for the data. PLOS journals do not allow statements supported by "data not shown" or "unpublished results." For such statements, authors must provide supporting data or cite public sources that include it.

*	We expect all researchers with submissions to PLOS in which author-generated code underpins the findings in the manuscript to make all author-generated code available without restrictions upon publication of the work. In cases where code is central to the manuscript, we may require the code to be made available as a condition of publication. Authors are responsible for ensuring that the code is reusable and well documented. Please make any custom code available, either as part of your data deposition or as a supplementary file. Please add a sentence to your data availability statement regarding any code used in the study, e.g. "The code used in the analysis is available from Github [URL] and archived in Zenodo [DOI link]" Please review our guidelines at <https://journals.plos.org/plosmedicine/s/materials-software-and-code-sharing> and ensure that your code is shared in a way that follows best practice and facilitates reproducibility and reuse. Because Github depositions can

be readily changed or deleted, we encourage you to make a permanent DOI'd copy (e.g. in Zenodo) and provide the URL.

*	FINANCIAL DISCLOSURES: The funding statement should include: specific grant numbers, initials of authors who received each award, URLs to sponsors’ websites. Also, please state whether any sponsors or funders (other than the named authors) played any role in study design, data collection and analysis, the decision to publish, or preparation of the manuscript. If they had no role in the research, include this sentence: “The funders had no role in study design, data collection and analysis, decision to publish, or preparation of the manuscript.”

*	COMPETING INTEREST: All authors must declare their relevant competing interests per the PLOS policy, which can be seen here: <https://journals.plos.org/plosmedicine/s/competing-interests>

For authors with ties to industry, please indicate whether any of the interests has a financial stake in the results of the current study.

*	DATA AVAILABILITY: Please include the details provided under "Data and Resource Availability" in the Data Availability statement in the online submission.

FORMATTING - GENERAL

*	Abstract: Please structure your abstract using the PLOS Medicine headings (Background, Methods and Findings, Conclusions). Please combine the Methods and Findings sections into one section.

*	At this stage, we ask that you include a short, non-technical Author Summary of your research to make findings accessible to a wide audience that includes both scientists and non-scientists. The Author Summary should immediately follow the Abstract in your revised manuscript. This text is subject to editorial change and should be distinct from the scientific abstract. Ideally each sub-heading should contain 2-3 single sentence, concise bullet points containing the most salient points from your study. In the final bullet point of 'What Do These Findings Mean?', please include the main limitations of the study in non-technical language. Please see our author guidelines for more information: <https://journals.plos.org/plosmedicine/s/revising-your-manuscript#loc-author-summary>.

*	Please express the main results with 95% CIs as well as p values. When reporting p values please report as p<0.001 and where higher as the exact p value p=0.002, for example. Throughout, suggest reporting statistical information as follows to improve clarity for the reader "22% (95% CI [13%,28%]; p</=)". Please be sure to define all numerical values at first use.

*	Please include page numbers and line numbers in the manuscript file. Use continuous line numbers (do not restart the numbering on each page).

*	Please cite the reference numbers in square brackets. Citations should precede punctuation.

FIGURES AND TABLES

*	Please provide titles and legends for all figures and tables (including those in Supporting Information files).

*	Please define all abbreviations used in each figure/table (including those in Supporting Information files).

*	Please consider avoiding the use of red and green in order to make your figure more accessible to those with color blindness.

SUPPLEMENTARY MATERIAL

*	Please note that supplementary material will be posted as supplied by the authors. Therefore, please amend it according to the relevant comments outlined here.

*	Please cite your Supporting Information as outlined here: <https://journals.plos.org/plosmedicine/s/supporting-information>

REFERENCES

*	PLOS uses the numbered citation (citation-sequence) method and first six authors, et al.

*	Please ensure that journal name abbreviations match those found in the National Center for Biotechnology Information (NCBI) databases (<http://www.ncbi.nlm.nih.gov/nlmcatalog/journals>), and are appropriately formatted and capitalised.

*	Where website addresses are cited, please include the complete URL and specify the date of access (e.g. [accessed: 12/06/2024]).

*	Please also see <https://journals.plos.org/plosmedicine/s/submission-guidelines#loc-references> for further details on reference formatting.

STUDY TYPE-SPECIFIC REQUESTS

*	Abstract: Please include the study design, population and setting, number of participants, years during which the study took place (enrollment and follow up), length of follow up, and main outcome measures.

*	Please ensure that the study is reported according to the STROBE (or appropriate STOBE extension) guideline (available from: <https://www.equator-network.org/reporting-guidelines/strobe>) and include the completed STROBE (or STROBE extension) checklist as Supporting Information. Please add the following statement, or similar, to the Methods: "This study is reported as per the Strengthening the Reporting of Observational Studies in Epidemiology (STROBE) guideline (S1 Checklist)." When completing the checklist, please use section and paragraph numbers, rather than page numbers.

*	For all observational studies, in the manuscript text, please indicate: (1) the specific hypotheses you intended to test, (2) the analytical methods by which you planned to test them, (3) the analyses you actually performed, and (4) when reported analyses differ from those that were planned, transparent explanations for differences that affect the reliability of the study's results. If a reported analysis was performed based on an interesting but unanticipated pattern in the data, please be clear that the analysis was data driven.

*	Please state in the Methods section whether the study had a prospective protocol or analysis plan. If a prospective analysis plan (from your funding proposal, IRB or other ethics committee submission, study protocol, or other planning document written before analyzing the data) was used in designing the study, please include the relevant document(s) with your revised manuscript as a Supporting Information file to be published alongside your study and cite it in the Methods section. A legend for this file should be included at the end of your manuscript. If no such document exists, please make sure that the Methods section transparently describes when analyses were planned, and when/why any data-driven changes to analyses took place. Changes in the analysis, including those made in response to peer review comments, should be identified as such in the Methods section of the paper, with rationale.

__________________________________________________

In compliance with data protection regulations, you may request that we remove your personal registration details at any time. (Use the following URL: <https://www.editorialmanager.com/pmedicine/login.asp?a=r>). Please contact the publication office if you have any questions.

**References**

1.	Srour B, Chazelas E, Druesne-Pecollo N, Esseddik Y, de Edelenyi FS, Agaësse C, et al. Dietary exposure to nitrites and nitrates in association with type 2 diabetes risk: Results from the NutriNet-Santé population-based cohort study. PLOS Med. 2023 Jan 17;20(1):e1004149.

2.	Debras C, Deschasaux-Tanguy M, Chazelas E, Sellem L, Druesne-Pecollo N, Esseddik Y, et al. Artificial Sweeteners and Risk of Type 2 Diabetes in the Prospective NutriNet-Santé Cohort. Diabetes Care. 2023 Jul 25;46(9):1681–90.

3.	Salame C, Srour B, Viennois E, Huybrechts I, Hercberg S, Deschasaux-Tanguy M, et al. Food emulsifiers and risk of type 2 diabetes: results from the prospective NutriNet-Santé cohort. Eur J Public Health. 2023 Oct 1;33(Supplement_2):ckad160.1248.

4.	Suez J, Cohen Y, Valdés-Mas R, Mor U, Dori-Bachash M, Federici S, et al. Personalized microbiome-driven effects of non-nutritive sweeteners on human glucose tolerance. Cell. 2022 Sep 1;185(18):3307-3328.e19.

5.	Chassaing B, Van de Wiele T, De Bodt J, Marzorati M, Gewirtz AT. Dietary emulsifiers directly alter human microbiota composition and gene expression ex vivo potentiating intestinal inflammation. Gut. 2017 Aug;66(8):1414–27.

6.	den Biggelaar LJCJ, Sep SJS, Mari A, Ferrannini E, van Dongen MCJM, Wijckmans NEG, et al. Association of artificially sweetened and sugar-sweetened soft drinks with β-cell function, insulin sensitivity, and type 2 diabetes: the Maastricht Study. Eur J Nutr. 2020 Jun 1;59(4):1717–27.

7.	Kopp B, Vignard J, Mirey G, Fessard V, Zalko D, Hgarat LL, et al. Environmental and Molecular Mutagenesis | EMGS Journal. Wiley Online Libr [Internet]. [cited 2024 Dec 11]; Available from: https://onlinelibrary.wiley.com/action/showCitFormats?doi=10.1002%2Fem.22214

8.	Graillot V, Takakura N, Hegarat LL, Fessard V, Audebert M, Cravedi JP. Genotoxicity of pesticide mixtures present in the diet of the French population. Environ Mol Mutagen. 2012 Apr;53(3):173–84.

9.	Recoules C, Touvier M, Pierre F, Audebert M. Evaluation of the toxic effects of food additives, alone or in mixture, in four human cell models. Food and Chemical Toxicology. In Press;

10.	Touvier M, Méjean C, Kesse-Guyot E, Pollet C, Malon A, Castetbon K, et al. Comparison between web-based and paper versions of a self-administered anthropometric questionnaire. Eur J Epidemiol. 2010 May 1;25(5):287–96.

11.	Lassale C, Castetbon K, Laporte F, Camilleri GM, Deschamps V, Vernay M, et al. Validation of a Web-based, self-administered, non-consecutive-day dietary record tool against urinary biomarkers. Br J Nutr. 2015 Mar 28;113(6):953–62.

12.	Lassale C, Castetbon K, Laporte F, Deschamps V, Vernay M, Camilleri GM, et al. Correlations between Fruit, Vegetables, Fish, Vitamins, and Fatty Acids Estimated by Web-Based Nonconsecutive Dietary Records and Respective Biomarkers of Nutritional Status. J Acad Nutr Diet. 2016 Mar;116(3):427-438.e5.

13.	Touvier M, Kesse-Guyot E, Méjean C, Pollet C, Malon A, Castetbon K, et al. Comparison between an interactive web-based self-administered 24 h dietary record and an interview by a dietitian for large-scale epidemiological studies. Br J Nutr. 2010/11/17 ed. 2011;105(7):1055–64.

14.	Lassale C, Péneau S, Touvier M, Julia C, Galan P, Hercberg S, et al. Validity of Web-Based Self-Reported Weight and Height: Results of the Nutrinet-Santé Study. J Med Internet Res. 2013 Aug 8;15(8):e152.

15.	Vergnaud AC, Touvier M, Méjean C, Kesse-Guyot E, Pollet C, Malon A, et al. Agreement between web-based and paper versions of a socio-demographic questionnaire in the NutriNet-Santé study. Int J Public Health. 2011 Aug 1;56(4):407–17.

16.	Beslay M, Srour B, Méjean C, Allès B, Fiolet T, Debras C, et al. Ultra-processed food intake in association with BMI change and risk of overweight and obesity: A prospective analysis of the French NutriNet-Santé cohort. PLoS Med. 2020 Aug 27;17(8):e1003256.

17.	Debras C, Chazelas E, Srour B, Druesne-Pecollo N, Esseddik Y, Szabo de Edelenyi F, et al. Artificial sweeteners and cancer risk: Results from the NutriNet-Santé population-based cohort study. PLOS Med. 2022 Mar 24;19(3):e1003950.

18.	Chaltiel D, Julia C, Adjibade M, Touvier M, Hercberg S, Kesse-Guyot E. Adherence to the 2017 French dietary guidelines and adult weight gain: A cohort study. PLOS Med. 2020 Dec 30;16(12):e1003007.

19.	van Gelder MMHJ, Bretveld RW, Roeleveld N. Web-based Questionnaires: The Future in Epidemiology? Am J Epidemiol. 2010 Dec 1;172(11):1292–8.

20.	Joinson A. Social desirability, anonymity, and internet-based questionnaires. Behav Res Methods Instrum Comput. 1999 Sep 1;31(3):433–8.

21.	Wang YC, Lee CM, Lew-Ting CY, Hsiao CK, Chen DR, Chen WJ. Survey of substance use among high school students in Taipei: web-based questionnaire versus paper-and-pencil questionnaire. JAdolescHealth. 2005 Oct;37(1879-1972 (Electronic)):289–95.

22.	Lailler G, Piffaretti C, Fuentes S, Nabe HD, Oleko A, Cosson E, et al. Prevalence of prediabetes and undiagnosed type 2 diabetes in France: Results from the national survey ESTEBAN, 2014–2016. Diabetes Res Clin Pract. 2020 Jul 1;165:108252.

23.	Lee D, Seung H. Algorithms for Non-negative Matrix Factorization. Adv Neural Inf Process Syst. 2001 Feb 10;13.

24.	Guo YT, Li QQ, Liang CS. The rise of nonnegative matrix factorization: Algorithms and applications. Inf Syst. 2024 Jul 1;123:102379.

25.	Zetlaoui M, Feinberg M, Verger P, Clémençon S. Extraction of food consumption systems by nonnegative matrix factorization (NMF) for the assessment of food choices. Biometrics. 2011 Dec;67(4):1647–58.

26.	Gazan R, Béchaux C, Crépet A, Sirot V, Drouillet-Pinard P, Dubuisson C, et al. Dietary patterns in the French adult population: a study from the second French national cross-sectional dietary survey (INCA2) (2006–2007). Br J Nutr. 2016/05/18 ed. 2016;116(2):300–15.

27.	Brunet JP, Tamayo P, Golub TR, Mesirov JP. Metagenes and molecular pattern discovery using matrix factorization. Proc Natl Acad Sci. 2004 Mar 23;101(12):4164–9.

28.	Pascual-Montano A, Carazo JM, Kochi K, Lehmann D, Pascual-Marqui RD. Nonsmooth nonnegative matrix factorization (nsNMF). IEEE Trans Pattern Anal Mach Intell. 2006 Mar;28(3):403–15.

29.	Touvier M, da Costa Louzada ML, Mozaffarian D, Baker P, Juul F, Srour B. Ultra-processed foods and cardiometabolic health: public health policies to reduce consumption cannot wait. The BMJ. 2023 Oct 10;383:e075294.

30.	ANSES. Etude Individuelle Nationale des Consommations Alimentaires 3 (INCA 3). 2017.

31.	Shi B, Choirat C, Coull BA, VanderWeele TJ, Valeri L. CMAverse: A Suite of Functions for Reproducible Causal Mediation Analyses. Epidemiology. 2021 Sep;32(5):e20.

32.	Chaltiel D, Adjibade M, Deschamps V, Touvier M, Hercberg S, Julia C, et al. Programme National Nutrition Santé – guidelines score 2 (PNNS-GS2): development and validation of a diet quality score reflecting the 2017 French dietary guidelines. Br J Nutr. 2019/07/25 ed. 2019;122(3):331–42.

33.	Andreeva VA, Deschamps V, Salanave B, Castetbon K, Verdot C, Kesse-Guyot E, et al. Comparison of Dietary Intakes Between a Large Online Cohort Study (Etude NutriNet-Santé) and a Nationally Representative Cross-Sectional Study (Etude Nationale Nutrition Santé) in France: Addressing the Issue of Generalizability in E-Epidemiology. Am J Epidemiol. 2016 Nov 1;184(9):660–9.

34.	Calixto Andrade G, Julia C, Deschamps V, Srour B, Hercberg S, Kesse-Guyot E, et al. Consumption of Ultra-Processed Food and Its Association with Sociodemographic Characteristics and Diet Quality in a Representative Sample of French Adults. Nutrients. 2021 Feb 20;13(2).
