# Supplementary material for: Food additive mixtures and type 2 diabetes incidence: Results from the NutriNet-Santé prospective cohort
Source: PLoS Med. 2025 Apr 8;22(4):e1004570. doi: 10.1371/journal.pmed.1004570 (PMC11977966; doi:10.1371/journal.pmed.1004570)
Supplement: Attachment — Submitted filename: PMEDICINE-D-24-03030_R2 - Point-by-point responses.docx [file pmed.1004570.s007.docx]

Review "Food additive mixtures and risk of type 2 diabetes: results from the NutriNet-Santé cohort" (PMEDICINE-D-24-03030R2) – Point-by-point responses to Editors and Reviewers’ comments

-----------------------------------------------------------

Comments from the academic editor: The authors addressed reviewers' comments provided previously, but some concerns remain.

- We thank the Academic Editor for his/her thorough analysis and insightful comments. All of them have been addressed below.

Two major comments are supplied hereafter, followed by minor comments.

1.
The authors failed to demonstrate the interactions/synergisms well. The authors tested statistical interactions, showing p-values in eTable 11. Significant interactions do not mean synergism because antagonism between two particular additives could happen. The authors should display the estimates of regression coefficients (+ 95% CI) of two single terms and an interaction term, three in total, for each test for presenting eTable 11. Each food additive may be studentized to mean=0 and SD=1 for interpretability, after the authors preset means and SDs of the food additives.

The tests for interaction miss the directionality. That is one of the issues in the current presentation. Second, the authors found a lot of null findings for interactions. It is unclear if the interaction effects explain the large fraction of the observed associations. Given the exploratory nature of the interaction following the significant testing of each cluster, the authors should tone down their inferences about interactions/synergisms, at least in the abstract.

In the abstract, the authors should not use the words "interaction/synergistic effects", as this study did not primarily evaluate the effects but evaluated the potential effect of a combination or cluster of multiple correlated additives and then explored the interaction.

- We thank the Academic Editor for this insightful comment. We now display in Table K (S1 Supporting Information, page 54) the estimates of regression coefficients (+ 95% CI) for each two single terms + interaction term. Each food additive has been standardized to mean=0 and SD=1 for interpretability. This has been added in the Methods line 285, page 12, in the Results lines 371-375, page 16, and the Discussion has been amended accordingly lines 431-435, page 18. These analyses revealed bi-directional interactions, suggesting potential synergies (with >0 beta coefficients) and antagonisms (<0 beta coefficients) between food additives emblematic of mixture 2 and mixture 5. Experimental research is needed to better understand these interactions and their role in type 2 diabetes aetiology. Following your advice, we have toned down inferences about interactions, in the abstract lines 64-65, page 2 (in particular, the words "interaction/synergistic effects" have been removed) and throughout the manuscript.

The following revisions should be made in the abstract:

Background:
The following sentence should be revised: "These findings suggest that potential interaction/synergistic effects of food additives should be considered in safety assessments..."
A revised sentence may be said, "These findings suggest that potential effects of multiple food additives should be considered in safety assessments..."

- This has been changed accordingly in the Abstract section (lines 72-74, page 3).

Results:
The authors should state that several interactions were indicated in their exploratory analyses for one of the clusters, with clear information on the directionality.

- Due to word number constraints, it was not possible to provide detailed numerical results here in the abstract, but the following sentence has been added (lines 64-65, page 2), mentioning the bi-directional nature of the interactions: “Several synergistic and antagonist interactions between food additives were detected in exploratory analyses”.

Discussion:
The authors stated, "This study revealed positive associations between exposure to two widely consumed food additive mixtures and increased type 2 diabetes risk. Further experimental research is needed to depict underlying mechanisms. These findings suggest that potential interaction/synergistic effects of food additives should be considered in safety assessments, and they support public health recommendations to limit non-essential additives."

Grammer is not right, partly. This sub-section should be revised, "This study revealed positive associations between exposure to two widely consumed food additive mixtures and higher type 2 diabetes incidence. Further experimental research is needed to depict underlying mechanisms, including potential interactions/synergistic effects. These findings suggest that a combination of food additives should be considered in safety assessments, and they support public health recommendations to limit non-essential additives."

- This section of the abstract has been changed accordingly (lines 69-74, page 3).

2.
This cohort seemed to be based on participants across France. The authors should clarify the geographical diversity and how they addressed the potential confounding due to geosocial factors.
Some cities are by the Mediterranean Sea, while other cities are mountainous, by the Atlantic Ocean or by the Strait of Dover. Cultural diversity was present, as well as racial and geosocial diversity.
Despite that, the authors adjusted only for education levels and professional occupation as socioeconomic factors. The degree of residual confounding owing to the nationwide diversity may be substantial.

The limitation section appears to include the limited opportunity of collecting race/ethnicity/religion. The authors are encouraged to document any such restriction in the method section, so that readers can digest the results with such an important limitation kept in mind.

- We added the repartition of the population study across 10 main geographical areas in Table 1 (pages 31-32) and we further adjusted our models for geographical location (eTable 7, now Table G, Model 7). This did not change the findings. The following sections have been modified accordingly: Methods line 274, page 12 and S1 Supporting Information Table G page 46.

We added precisions regarding the process of race/ethnicity/religion data in the Methods section lines 170-172, page 8: « Ethnicity and religion were not recorded since these variables are considered sensitive data by the French law, which strictly regulate their collection in population-based studies. »

Minor comments:

Hazard ratios presented in the abstract are not interpretable because of no information on the unit of the exposure. Please clarify.

- HRs are per an increment of 1SD of the NMF mixture score. We clarified this point in the abstract, lines 58-59, page 2.

Line 116-117: The authors stated, "These associations were not driven by a specific food additive, and several interactions between additives of the same mixture were detected." This interpretation is misleading. There may be single independent effects of several food additives. The effect of an interaction could be minor. Also, the effect of an interaction could be antagonistic, being outweighed by positive independent single effects. As implied above, the authors should give a sentence that implies multiple interpretations.

- We agree with this comment and acknowledge that the previous phrasing was too simplistic. We have rephrased this section to reflect the bi-directional nature of detected interactions, the exploratory nature of these interaction analyses, as well as the need for experimental research to further depict the potential synergisms or antagonisms between food additives: “Exploratory analyses suggested both synergistic and antagonist interactions between several food additives emblematic of these mixtures.” (lines 103-104, page 4).

Line 127-128: It sounds too early to say "should be", given the presentation of eTable 11 with many negative results and without any replication or biologically plausible explanations. The authors may rephrase, "the potential interactions/synergism may be of interest in future mechanistic investigations and considerations in safety assessment".

The authors also have an issue with measurement errors that are common in any observational research of this kind, owing to the dietary assessment undertaken and food-additive databases used. As the authors noted in the discussion section, those limitations must have mattered. Accounting for multiple epidemiological issues, the authors should follow the suggestions given above.

- We have rephrased this sentence to be more careful (lines 112-114, page 5) and followed all suggestions above.

Line 134: "a major hypothesis" should be rephrased to "one of the hypotheses". There is no evidence that the authors' argument is right.

- This has been changed accordingly (line 122, page 6).

Line 160-163: After this statement of the primary aim, the authors may want to state their exploratory, secondary aim to examine interactions between food additives in a cluster associated with type 2 diabetes incidence.

- This exploratory secondary aim has been added at the end of the introduction, lines 151-153, page 7.

Line 170: If the recruitment was ongoing, there should be a calender time at which the authors formed a cohort for this specific study. For example, it is unlikely that the authors included participants recruited when this manuscript was being written up. The authors should clarify the calendar time when the participants in this specific study were recruited.

- All participants enrolled between the start of the cohort on May 11^th^, 2009 and December 31^st^, 2023, were eligible to be included in this study. We clarified this point in the Methods section, line 163, page 7.

Line 188: "are" should be "were".

- Sorry for the typo, this has been changed accordingly line 182, page 8.

Line 189: "validated" should be taken out. There is no possibility that any measures of a dietary assessment tool are perfectly valid. What is appropriate is to present how valid main dietary variables would be for the specific hypothesis to test. In this study, the authors failed to provide such information, so "validated" should be taken out, and some degree of validity should not be indicated in this manuscript.

- « Validated » has been taken out accordingly throughout the paper.

Line 228: "is" should be "was".

- This has been corrected line 223, page 10.

Line 341: "risk" should be incidence. The authors should review their text not to misuse risk and incidence. The authors evaluated time-to-event data and analyzed incidence, not risk. It may be acceptable sometimes to use "risk" when inferring a risk. However, when the authors document results derived from their analyses of incidence (N cases / person-time), they should use properly the term, "incidence". It would be shocking if the authors do not understand the difference between risk and incidence.

- The term “risk” has been replaced by “incidence" everywhere in the manuscript and supplemental for results derived from analyses of incidence.

Line 358-359: This description should be elaborated more in this text. The authors are interested in interactions/synergisms, but the descriptions of the resulst are overly simple, and no one can judge the strength of evidence or identifiy the numbers of null results and directionality of the potential interactions.

- We expanded the description of the interactions in the Results section (lines 371-375, page 16): “Out of the 28 two-by-two interactions tested between the 8 additives most emblematic of mixture 2, 3 were detected (i.e. p for interaction <0.1) with beta coefficient >0 (suggesting synergism) and 4 were detected with beta coefficient <0 (suggesting antagonism). For mixture 5, 91 interactions were tested between the 14 most emblematic additives: 6 were detected with beta coefficient >0 and 4 with beta coefficient <0 (Table K in S1 Supporting Information).”

Line 387: "supporting potential synergistic effects". As mentioned above, the directions of associations were not presented; it is unclear if synergisms were reasonably indicated. The authors should present results sufficiently so that the authors' arguments make sense.

- We removed “supporting potential synergistic effects” and replaced by “Exploratory analyses suggested both synergistic and antagonist interactions between several food additives emblematic of these mixtures.” lines 396-398, page 17.

Line 413-415: These hypotheses were not given in the introductory section. At the very least, the brief intention should be documented as suggested above. It is odd to see the authors clarify their hypothesis in the discussion section.

- As also requested above, we have added this exploratory aim in the Introduction section, lines 151-153, page 7.

Line 415-419: Confirm those with the directions of the interaction estimates.

- We have rephrased this section (lines 431-435, page 18) as such: “Besides, several interactions have been detected between emblematic additives of mixtures 2 and 5. However, the number of detected interactions was limited compared to the overall number tested. Moreover, both synergistic, but also antagonist interactions were observed. The relative influence of individual additives versus their interactions should be explored in future mechanistic investigations.”

Line 449: "highlighting" should be rephrased to "indicating". As repeatedly implicated, the presence of an effect of a specific mixture does not necessarily mean an interaction/synergism.

- We deleted this part of the sentence accordingly to avoid suggesting interaction/synergism (line 463, page 19).

Line 511: Misclassification of diabetes cases as non-cases, considering undiagnosed diabetes as non-cases, is likely to depend on socioeconomic status and health consciousness. Also, if the size of cohort is big, non-differential misclassification can be overwhelming. The authors should eliminate the argument as if the impact was negligible. (Basically, the authors misunderstood the nature of outcome misclassification. Some outcome misclassifications, such as low positive predictive value, may cause just a power issue, but not necessarily the case).

- We eliminated the argument as if the impact was negligible in the Discussion section, line 518, page 22.

Line 517-518: Too weak argument. Take it out. Consistency with national averages should not happen when the assessment was valid, as this study recruited more women than men, for example. The authors do not need to supply weak arguments as no study is perfect anyway.

- We removed this argument in the Discussion section line 524, page 22.

----------------------------------------------------------
Comments from the reviewers:

Reviewer #1: The authors have addressed my points.

Michael Dewey

Reviewer #2: Thank you for addressing carefuly the comments raised previously. I have no further comments.

Any attachments provided with reviews can be seen via the following link: ********

-----------------------------------------------------------
Requests from Editors:

TITLE

Please check the formatting of your title (Is there a line break following the colon?). Please check that the title is according to PLOS Medicine's style. Your title must be nondeclarative and not a question. It should begin with main concept if possible. "Effect of" should be used only if causality can be inferred, i.e., for an RCT. Please place the study design ("A randomized controlled trial," "A retrospective study," "A modelling study," etc.) in the subtitle (i.e., after a colon).

- The study design has been added to the subtitle, and the title format has been revised accordingly.

ABSTRACT

1) l.59ff: Please replace ‘y’ with ‘years’ or define ‘y’ at first use. We would prefer the first option.

- This has been changed accordingly, lines 48-49, page 2.

2) l.59ff: Please define ‘SD’ at first use. Throughout the abstract and main text, please ensure that abbreviations, including statistical abbreviations, are defined the first time they are used.

- The standard deviation has been added in the abstract the first time it is mentioned. We checked that abbreviations were defined the first time they were used.

3) ll.59-60: For clarity, we suggest changing the sentence to: “Participants (n=108,643, mean follow-up = 7.7 years (standard deviation (SD) = 4.6), age = 42.5 years (SD = 14.6), 79.2% women) were adults from the French NutriNet-Santé cohort (2009-2023).”

- This has been changed accordingly, lines 48-50, page 2.

4) l.68: When reporting 95% CIs please separate upper and lower bounds with commas instead of hyphens as the latter can be confused with reporting of negative values. Please revise throughout the manuscript. Also, please remember to introduce abbreviations, such as ‘HR’.

- This has been updated accordingly and checked throughout the manuscript and supplemental.

5) We feel that for full transparency you should also report the results, i.e. HR values, for the three food mixtures that did not show an association with increased diabetes risk.

- These results have been added accordingly, lines 62-65, page 2.

6) In the last sentence of the Abstract Methods and Findings section, please describe the main limitation(s) of the study's methodology.

- A description of the main limitations of the study’s methodology has been added at the end of the Methods and Findings section: « Residual confounding as well as exposure or outcome misclassifications cannot be entirely ruled out and causality cannot be established based on this single observational study. », lines 65-67, pages 2-3.

7) Please ensure that all numbers presented in the abstract are present and identical to numbers presented in the main manuscript text.

- This has been thoroughly verified.

9) Please include the important dependent variables that are adjusted for in the analyses.

- We have added information regarding potential confounders accounted for in the analyses, lines 54-55, page 2: “…associations with type 2 diabetes incidence were assessed using Cox models adjusted for potential socio-demographic, anthropometric, lifestyle and dietary confounders.”.

AUTHOR SUMMARY

1) Please remove any numerical results from the Author Summary.

2) Please temper claims of primacy of results by stating, "to our knowledge" (or something similar) or remove ‘is the first’.

3) We suggest changing the order of the bullet points under ‘What do these findings mean?’:
• The study results suggest that food additives found in a wide variety of products and frequently consumed together may potentially represent a modifiable risk factor for type 2 diabetes prevention. They support public health recommendations to limit non-essential additives.
• Potential interaction/synergistic effects of food additives should be considered in future safety assessments.
• Confirmation by other epidemiological and experimental studies will be necessary to support causality of the observed associations.

4) Please note that in the final bullet point of ‘What Do These Findings Mean?’ the main limitations of the study should be included in non-technical language (i.e. the above might require further changes).

- The author summary has been modified accordingly (and following the recommendations of the Academic Editor).

INTRODUCTION

1) If there has been a systematic review of the evidence related to your study (or you have conducted one), please refer to and reference that review and indicate whether it supports the need for your study.

- No systematic review published per se since this study is the first to quantity exposure to food additive mixtures and study their associations with diabetes risk in a population-based study (verified by literature search in Pubmed, but not published).

2) l.136ff: Please ensure to provide references.

- A reference has been added (line 123, page 6).

3) ll.153-155: We suggest changing to: “Another important gap to date has been that previous evaluations have not been able to account for potential interaction/synergistic effects when assessing the safety of additives due to a lack of data.”

- Done (lines 141-143, pages 6-7).

4) l.155: Please use the abbreviation 'UPF' instead of 'ultra-processed foods' as you introduced the abbreviation early in the Introduction. Please revise throughout.

- Done (line 122, page 6, and throughout).

5) ll.158-160: Please revise for grammar/clarity.

- Done (line 143-146, page 7).

6) l.161: Please temper claims of primacy of results by stating, "to our knowledge" (or something similar).

- “To our knowledge” has been added (line 149, page 7).

7) l.163: We suggest writing '...using the large prospective NutriNet-Santé cohort'.

- This sentence has been rephrased following the advice of the Academic Editor (line 150, page 7).

METHODS AND RESULTS

1) ll.223-224: We suggest providing a brief explanation of ‘under-energy reporters’ (e.g. in parenthesis).

- We have added the precision: “(i.e. participants who systematically reported implausibly low energy intakes)” (line 186, page 8).

2) l.251: Please spell out ‘T1D’ as you have not introduced the abbreviation.

- Done (type 1 diabetes) (line 249, page 11).

3) l.295: The terms gender and sex are not interchangeable (as discussed in https://www.who.int/health-topics/gender#tab=tab_1 ); please use the appropriate term (also in the Abstract).

- Biological sex (not gender) is used as adjustment factor in Cox models.

4) l.295, we suggest changing this to "At baseline, the median age of the cohort was ..." to avoid that "their" refers only to females.

- Done (lines 299-300, page 13).

5) ll.298-299: “Participants completed a median of 5 dietary records (25th – 75th percentiles: 3-9).” – does this refer to the entire NutriNet-Santé cohort or to the participants specifically included in this study? Please revise the characteristics paragraph for clarity.

- Participants included in this study -> we have clarified this point line 303, page 13.

6) ll.324-317: We suggest splitting the sentence into two for clarity.

- Done (lines 331-334, page 14).

7) l.332: Please remove the word ‘famous’.

- Done (line 339, page 14).

8) l.345: If you agree, we feel it is worth repeating here (briefly) the types of sensitivity analyses that have been carried out.

- Done (lines 354-358, page 15): “These models tested several further or modified adjustments (for prevalent metabolic disorders; for other mixtures - mutual adjustment; for Healthy and Western dietary patterns; for food groups instead of nutrients; for health-seeking behaviours; and for geographical region), and exclusion of cases diagnosed in the first two years of follow-up to challenge reverse causality.”

9) l.353ff: Your study is observational and therefore causality cannot be inferred. Please remove language that implies causality, such as effect. Please refer to associations instead. Please revise throughout (including the discussion).

- This has been checked throughout.

10) l.354: Please note that you have not yet introduced the abbreviation 'T2D' and we would suggest that you continue to write 'type 2 diabetes'. Please revise throughout.

- Type 2 diabetes is not spelled out throughout.

11) Table 1: Please ensure that all abbreviations below the table, such as BMI and SD, are defined. Please also refer to comment 3) and revise accordingly (sex versus gender).

- All abbreviations are defined below tables.

As stated above, biological sex (not gender) is used in this study.

12) Table 2: Please indicate the meaning of the values following the food additive in the table heading.

- Done.

13) Figure 1: Please include a definition of the five mixtures in the figure description. Please be sure to define all abbreviations (SD, BMI, CI, etc.). Please add a title for the x-axis. Please replace the hyphens with commas.

- We included a definition of the 5 mixtures in the figure’s legend.

Abbreviations were defined, and the plot was modified accordingly.

14) Figure 2: The image quality is very poor and, for example, the y-axis title is barely readable. Please revise the axes titles. Please also add a definition of mixture 2 and 5.

- We improved the resolution of Figure 2, revised the axes titles and included a definition of mixtures 2 and 5 in the legend.

15) Figures 1 and 2 appear rather small at the moment. Please revise, also with regard to the comment about image quality.

- We improved image quality for Figures 1 and 2.

DISCUSSION

General guidance: Please present and organize the Discussion as follows: a short, clear summary of the article's findings; what the study adds to existing research and where and why the results may differ from previous research; strengths and limitations of the study; implications and next steps for research, clinical practice, and/or public policy; one-paragraph conclusion.

- We checked that the discussion now follows this general guidance.

1) ll.431-434: Please ensure to indicate that these research findings result from experiments in mice. Please revise throughout.

- We have added this precision for ref xx (lines 448 and 456, page 19) and checked that the type of experimental model is specified for all cited studies.

2) l.443: “type 2 diabetes patients” - PLOS Medicine prefers the use of patient-centered language, e.g. patients with type 2 diabetes. Please revise throughout.

- The phrasing “patients with type 2 diabetes” is now used (one occurrence only, line 457, page19).

3) l.522: Please temper claims of primacy of results by stating, "to our knowledge" (or something similar).

- Done (throughout).

4) Please remove any subheadings.

- Done.

REFERENCES

1) Where website addresses are cited, please use the word ‘accessed’ when specifying the date of access (e.g. [accessed: 12/06/2024]).

- Done.

2) Please make sure to check and update the references where necessary (e.g. [9]).

- We checked all references, and added number of pages etc. for this refence (now reference n°10) that has been published in the meantime.

SUPPLEMENTARY MATERIAL

1) Please ensure that all supplementary files are referenced in the main text.

- This has been checked throughout.

2) In the published article, supporting information files are accessed only through a hyperlink attached to the captions. For this reason, you must list captions at the end of your manuscript file. You may include a caption within the supporting information file itself, as long as that caption is also provided in the manuscript file. Do not submit a separate caption file.

- The table of content of the document “S1 Supporting Information” has been provided at the end of the manuscript file.

When SI files are contained with a single file:

Please label the file as ‘S1 Supporting Information’.

Please apply alphabetical labelling to each table and figure contained within the S1 file. For example, ‘Fig A’ to ‘Fig Z’ and ‘Table A’ to ‘Table Z’.

Plain text does not need to be labelled and can just be given a title as necessary. For example, ‘Statistical Analysis Plan’.

Please cite tables/figures as ‘Fig A in S1 Supporting Information’ and/or ‘Table A in S1 Supporting Information’, for example.

Please cite plain text as, ‘Statistical Analysis Plan in S1 Supporting Information’, for example.

When SI files are uploaded as separate files:

Please label tables as ‘S1 Table’ (so on) and figures as ‘S1 Fig’ (and so on).

Any additional documents (protocols/analysis plans etc.) can be labelled as ‘S1 Protocol’, for example. Please cite items as exactly as labelled.

- These modifications have been made accordingly.

-----------------------------------------------------------
General Editorial Requests

1) We ask every co-author listed on the manuscript to fill in a contributing author statement. If any of the co-authors have not filled in the statement, we will remind them to do so when the paper is revised. If all statements are not completed in a timely fashion this could hold up the re-review process. Should there be a problem getting one of your co-authors to fill in a statement we will be in contact. YOU MUST NOT ADD OR REMOVE AUTHORS UNLESS YOU HAVE ALERTED THE EDITOR HANDLING THE MANUSCRIPT TO THE CHANGE AND THEY SPECIFICALLY HAVE AGREED TO IT.

- Contributorship statement and guarantor is provided page 23.

2) Please ensure that the paper adheres to the PLOS Data Availability Policy (see http://journals.plos.org/plosmedicine/s/data-availability), which requires that all data underlying the study's findings be provided in a repository or as Supporting Information. For data residing with a third party, authors are required to provide instructions with contact information for obtaining the data. PLOS journals do not allow statements supported by "data not shown" or "unpublished results." For such statements, authors must provide supporting data or cite public sources that include it.

- Information regarding data access is provided in the Data and Resource Availability paragraph lines 590-598, page 24.

3) Please review your reference list to ensure that it is complete and correct. If you have cited papers that have been retracted, please include the rationale for doing so in the manuscript text, or remove these references and replace them with relevant current references. Any changes to the reference list should be mentioned in the rebuttal letter that accompanies your revised manuscript.

- The reference list has been reviewed.

*In compliance with data protection regulations, you may request that we remove your personal registration details at any time. (Use the following URL: https://www.editorialmanager.com/pmedicine/login.asp?a=r). Please contact the publication office if you have any questions.*
